# Exercise-based cardiac rehabilitation for coronary heart disease: a meta-analysis

**DOI:** 10.1093/eurheartj/ehac747

**Published:** 2023-01-02

**Authors:** Grace O Dibben, James Faulkner, Neil Oldridge, Karen Rees, David R Thompson, Ann-Dorthe Zwisler, Rod S Taylor

**Affiliations:** MRC/CSO Social and Public Health Sciences Unit, Institute of Health and Well Being, University of Glasgow, Glasgow, UK; School of Sport, Health and Community, Faculty Health and Wellbeing, University of Winchester, Winchester, UK; College of Health Sciences, University of Wisconsin-Milwaukee, Milwaukee, WI, USA; Division of Health Sciences, Warwick Medical School, University of Warwick, Coventry, UK; School of Nursing and Midwifery, Queen’s University Belfast, Belfast, UK; REHPA, The Danish Knowledge Centre for Rehabilitation and Palliative Care, Odense University Hospital, Nyborg, Denmark; Department of Clinical Research, University of Southern Denmark, Odense, Denmark; Department of Cardiology, Odense University Hospital, Odense, Denmark; MRC/CSO Social and Public Health Sciences Unit, Institute of Health and Well Being, University of Glasgow, Glasgow, UK; Robertson Centre for Biostatistics, Institute of Health and Well Being, University of Glasgow, Glasgow, UK

**Keywords:** Coronary heart disease, Cardiac rehabilitation, Exercise training, Physical activity, Prevention

## Abstract

**Aims:**

Coronary heart disease is the most common reason for referral to exercise-based cardiac rehabilitation (CR) globally. However, the generalizability of previous meta-analyses of randomized controlled trials (RCTs) is questioned. Therefore, a contemporary updated meta-analysis was undertaken.

**Methods and results:**

Database and trial registry searches were conducted to September 2020, seeking RCTs of exercise-based interventions with ≥6-month follow-up, compared with no-exercise control for adults with myocardial infarction, angina pectoris, or following coronary artery bypass graft, or percutaneous coronary intervention. The outcomes of mortality, recurrent clinical events, and health-related quality of life (HRQoL) were pooled using random-effects meta-analysis, and cost-effectiveness data were narratively synthesized. Meta-regression was used to examine effect modification. Study quality was assessed using the Cochrane risk of bias tool. A total of 85 RCTs involving 23 430 participants with a median 12-month follow-up were included. Overall, exercise-based CR was associated with significant risk reductions in cardiovascular mortality [risk ratio (RR): 0.74, 95% confidence interval (CI): 0.64–0.86, number needed to treat (NNT): 37], hospitalizations (RR: 0.77, 95% CI: 0.67–0.89, NNT: 37), and myocardial infarction (RR: 0.82, 95% CI: 0.70–0.96, NNT: 100). There was some evidence of significantly improved HRQoL with CR participation, and CR is cost-effective. There was no significant impact on overall mortality (RR: 0.96, 95% CI: 0.89–1.04), coronary artery bypass graft (RR: 0.96, 95% CI: 0.80–1.15), or percutaneous coronary intervention (RR: 0.84, 95% CI: 0.69–1.02). No significant difference in effects was found across different patient groups, CR delivery models, doses, follow-up, or risk of bias.

**Conclusion:**

This review confirms that participation in exercise-based CR by patients with coronary heart disease receiving contemporary medical management reduces cardiovascular mortality, recurrent cardiac events, and hospitalizations and provides additional evidence supporting the improvement in HRQoL and the cost-effectiveness of CR.

## Structured Graphical Abstract

**Structured graphical abstract:**
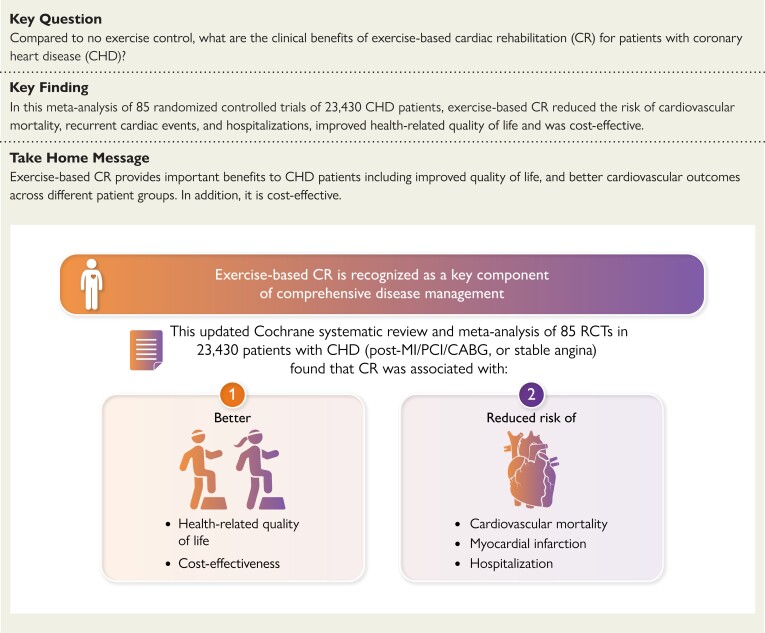
Exercise-based CR is recognized as a key component of comprehensive disease management. CABG, coronary artery bypass graft; CHD, coronary heart disease; MI, myocardial infarction; PCI, percutaneous coronary intervention; RCTs, randomized controlled trials.

**See the editorial comment for this article ‘Evidence is indisputable that cardiac rehabilitation provides health benefits and event reduction: time for policy action’, by S. L. Grace, https://doi.org/10.1093/eurheartj/ehac690.**

## Introduction

Coronary heart disease (CHD) is the most common cause of death globally.^1,2^ With increasing numbers of people living longer with CHD, accessible and effective health services for the management of CHD are crucial. Exercise-based cardiac rehabilitation (CR) is recognized as a key component of comprehensive CHD management and is a Class I Grade A recommendation in international guidelines.^3,4^

Although meta-analyses of randomized controlled trials (RCTs) have shown the beneficial effect of CR in patients with CHD,^5–7^ this evidence base has been questioned on the grounds of: (i) uncertainty in the impact on mortality; (ii) lack of data on health-related quality of life (HRQoL); (iii) inclusion of RCTs limited to low-risk patients and conducted in high-income country settings, and (iv) lack of trials conducted during the era of modern CHD therapy.^7–9^

To address these uncertainties, we undertook a contemporary update of the Cochrane systematic review and meta-analyses of RCTs to assess the effects of exercise-based CR in patients with CHD on mortality, clinical events, HRQoL, and cost-effectiveness. We also sought to explore whether intervention effects varied with patient case mix, study and intervention characteristics, and CR delivery settings.

## Methods

We conducted and reported this meta-analysis in accordance with the Cochrane Handbook for Interventional Reviews and the Preferred Reporting Items for Systematic Reviews and Meta-Analyses and the synthesis without meta-analysis statements, respectively.^10–12^

### Search strategy and study selection

We undertook update literature searches of Cochrane Central Register of Controlled Trials (CENTRAL), MEDLINE, Embase, CINAHL, and Science Citation Index Expanded from June 2014 (the search end date of the Cochrane 2016 review^5^) to September 2020 (strategy provided in Supplementary material online, *File S1*). We also searched two clinical trials registers (World Health Organization’s International Clinical Trials Registry Platform and ClinicalTrials.gov), and hand-searched reference lists of retrieved articles and recent systematic reviews. Records collected from trial registry searches were used to identify trials not picked up in database searches, as well as ongoing studies. We sought RCTs of exercise-based CR (exercise training alone or in combination with psychosocial or educational interventions) compared with no-exercise or usual care control, with at least 6-month post-baseline follow-up outcome measures. All patients in both the intervention and control groups were generally reported to receive (local or national) guideline recommended medical treatment.

Two reviewers (G.O.D. and J.F.) independently confirmed trial eligibility. Disagreements were resolved by discussion or by a third reviewer (R.S.T.), if necessary.

### Patient population

We included adults (≥18 years), in either hospital-or community-based settings, who had a myocardial infarction (MI), who had undergone revascularization [coronary artery bypass grafting (CABG) or percutaneous coronary intervention (PCI)], or who had angina pectoris or coronary artery disease defined by angiography.

### Data abstraction and quality appraisal

Two reviewers (G.O.D. and J.F.) independently completed data extraction and assessed study quality using the Cochrane Risk of Bias (ROB) tool,^13^ which was checked by a third reviewer (R.S.T.). Trials were assessed based on random sequence generation, allocation concealment, blinding of outcome assessment, incomplete outcome data, and selective reporting. Information regarding study methods (country, design, follow-up, and setting), participant characteristics (numbers randomized, age, sex, diagnosis, and inclusion/exclusion criteria), intervention (exercise mode, duration, frequency, intensity), and control (description, i.e. usual care, no exercise), outcomes, funding sources, and notable author conflicts of interest were obtained.

### Outcomes and certainty of evidence

Clinical event outcomes included overall and cardiovascular (CV) mortality, fatal and/or non-fatal MI (as reported by studies), CABG, PCI, overall hospitalization, and CV hospitalization. Other outcomes included HRQoL and CR costs, and cost-effectiveness per quality-adjusted life year (QALY). One reviewer (G.O.D.) assessed the certainty of the evidence using Grading of Recommendations Assessment, Development, and Evaluation (GRADE),^14,15^ and had it checked by a second reviewer (R.S.T.). GRADE assessment was applied to clinical event outcomes (overall and CV mortality, fatal and/or non-fatal MI, CABG, PCI, overall hospitalization, and CV hospitalization) at 6–12 months follow-up, the most frequently reported follow-up time point across trials. Evidence was downgraded from high certainty by one level based on the following domains: limitations in study design or execution (ROB), inconsistency of results, indirectness of evidence, imprecision, and publication bias.

### Statistical analysis

Outcome data were pooled at the longest reported follow-up and at three separate time periods: ‘short-term’ (6–12 months), ‘medium-term’ (13–36 months), and ‘long-term’ (>36 months) follow-up. Given the level of clinical heterogeneity (variation in CR interventions and populations), we purposively undertook random-effects meta-analyses, using the DerSimonian and Laird random-effects meta-analysis method, assuming that each study estimates a different underlying intervention effect. Dichotomous outcomes (overall and CV mortality, MI, CABG, PCI, and all-cause hospitalization, and CV hospitalization) are expressed as risk ratios (RRs) with 95% confidence intervals (CIs). For those clinical event outcomes with significant risk reductions, we calculated the number needed to treat for an additional beneficial outcome (NNT).^16^ Where ≥2 trials reported the same validated HRQoL measures and domains [i.e. Short-Form-36 (SF-36), EuroQol-5D (EQ-5D)], continuous outcomes were pooled separately by each scale and reported as the mean difference (MD) and 95% CI. Given the heterogeneity in HRQoL outcome measures and reporting, for comprehensiveness, we used a vote-counting approach to synthesis in addition to meta-analyses, where the number of positive, negative, and non-significant results was summed. Cost-effectiveness data were synthesized narratively. Statistical heterogeneity was considered substantial where *I*^2^ statistic >50%. For outcomes with ≥10 trials included in the meta-analysis, we used the funnel plot and Egger’s test to examine small study bias.^17^ The two-sided *P*-values <0.05 were considered statistically significant. A univariate random-effects meta-regression was used to explore heterogeneity and examine the following pre-defined treatment effect modifiers across clinical event outcomes only: (i) case mix (patients percentage presenting with MI), (ii) ‘dose’ of exercise [dose (units) = number of weeks of exercise training × average sessions per week × average duration of each session in min], (iii) type of CR (exercise only vs. comprehensive CR), (iv) length of follow-up (longest follow-up used where multiple time points are assessed), (v) publication year, (vi) sample size, (vii) CR setting (home or centre based), (viii) ROB (low in <3 of 5 domains), (ix) study continent (Europe, North America, Australia/Asia, or other), and (x) study country status [low-middle-income countries (LMICs) or high-income countries] according to the World Bank Group^18^. Given the number of statistical comparisons performed in this review, the results interpretation was primarily based on 95% CIs rather than *P*-values. Statistical analyses were performed in RevMan Web version 3.12.1 and STATA version 16.1.

## Results

### Search and selection of studies

**Figure 1:**
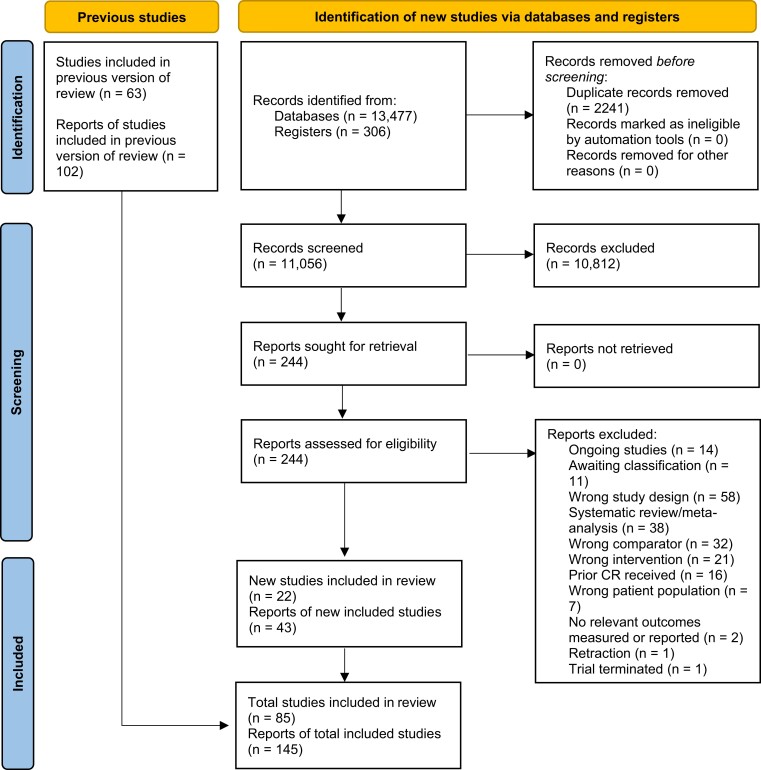
Preferred Reporting Items for Systematic Reviews and Meta-Analyses flow diagram of study selection process.

The search selection process is summarized in *Figure 1*. Updated database and trial registry searches resulted in a total of 13 783 hits, of which 11 056 unique records were identified, and 244 were selected for full-text review. The main reasons for exclusion were study design (e.g. non-RCT, <6-month follow-up), or use of exercise comparators. The 22 new RCTs (7795 participants; 43 publications),^19–40^ identified in this update, provide a total evidence base of 85 RCTs (145 publications, 23 430 participants) comparing exercise-based CR with a no-exercise control group in patients with CHD.^19–103^ The participants in the newly included trials represent about one-third of all participants included in this study (33%). A complete list of primary and associated supplementary references for included studies is provided in Supplementary material online, *File S2.*

A summary of the study, participant, intervention, and comparator characteristics of the 85 included studies is presented in *Table 1*. Seventy-nine (93%) of the 85 studies were two-arm parallel RCTs, with four studies comparing more than two arms, (two types of CR vs. control),^21,24,32,89^ one study using quasi randomization methods,^38^ and one cluster RCT.^62^ Sixteen of the 22 new trials identified were undertaken in LMICs,^19–21,24–26,28,30–34,37–40^ resulting in a total of 21 RCTs in LMICs. Three large multicentre trials contributed a total of 8956 participants (∼40% overall).^34,98,99^ The median age of participants across studies was 56 years, and over the last decade, the percentage of female patients included in trials increased from 11% to 17%. The median CR intervention duration and trial follow-up were 6 and 12 months, respectively. Thirty-eight of the 85 (45%) interventions were exercise only,^20–24,28,31–33,35,39–44,48,49,52,54,59,60,65,69,73,76,77,82–84,88–92,94,100^ with 47 (55%) involving multiple components including education (20 trials),^25,26,29,34,37,38,51,53,55,57,61,62,70,78,85–87,97,101,102^ psychosocial (seven trials),^36,46,58,72,74,80,95^ or a combination of both (16 trials),^19,30,45,50,63,64,66–68,71,75,93,96,98,99,103^ or other components (i.e. controlled diet, risk factor management, smoking cessation, relaxation; four trials).^27,47,79,81^ Exercise was typically aerobic, with the inclusion of resistance training reported in 27% trials (23 out of 85).^22,27,28,30,35,39,41,43,44,46,47,50,54,65,69,77,83,86,89,90,100–102^ The dose of exercise interventions varied widely, with frequency ranging between 1 and 7 sessions per week, length of sessions ranging between 20 and 90 min, and intensity ranging between 50% and 90% of maximal or peak heart rate, 50%–95% of aerobic capacity, or at a rating of perceived exertion between 11 and 16. Of the 21 home-based exercise programmes,^25,29,30,36,38,43–45,53,57,61–63,66,71,72,76,78,79,82,97^ four were delivered electronically via mobile phones or the internet.^25,29,72,82^

**Table 1:** Summary of study, population, intervention, and comparator characteristics

Study characteristics	Number of studies (%) or median of study means (range)
Publication year	
ȃ1970–9	2 (2%)
ȃ1980–9	12 (14%)
ȃ1990–9	20 (24%)
ȃ2000–9	21 (25%)
ȃ2010–9	23 (27%)
ȃ2020 onwards	7 (8%)
Study continent	
ȃEurope	48 (56%)
ȃNorth America	13 (15%)
ȃAsia	16 (19%)
ȃAustralia	5 (6%)
ȃOther	3 (4%)
LMIC	21 (25%)
Single centre	61 (72%)
Sample size	137 (25–3959)
Duration of follow-up, months	12 (6–228)
Population Characteristics	
Sex	
ȃMales only	21 (25%)
ȃFemales only	1 (1%)
ȃBoth males and females	61 (72%)
ȃNot reported	2 (2%)
Age, years	56 (44–77)
Diagnosis	
ȃPost-MI only	40 (47%)
ȃRevascularization only	14 (16%)
ȃAngina only	5 (6%)
ȃMixed CHD population	25 (29%)
ȃOther^a^	1 (1%)
*Intervention characteristics*	
Intervention type	
ȃExercise only programme	38 (45%)
ȃComprehensive programme	47 (56%)
Dose of intervention	
ȃDuration	6 months (0.75–42)
ȃFrequency	1–7 sessions/week
ȃLength	20 to 90 min/session
ȃIntensity	50%–90% maximal/peak HR or HRR 50%–95% VO_2_ max Borg RPE 11–16
Setting	
ȃCentre-based only	40 (47%)
ȃCombination of centre and home	21 (25%)
ȃHome-based only	21 (25%)
ȃNot reported	3 (3%)
Comparator	
ȃUsual/standard care	50 (59%)
ȃUsual care plus^b^	24 (28%)
ȃ‘No exercise’	8 (9%)
ȃOther	3 (4%)

CHD, coronary heart disease; HR, heart rate; HRR, heart rate reserve; LMIC, low-middle-income country; RPE, ratings of perceived exertion; VO_2_max, maximal oxygen uptake.

He 2020 recruited patients with MI in the absence of obstructive coronary artery disease.

Usual care plus education, guidance or advice about diet and exercise, but no formal exercise training.

### Risk of bias and GRADE assessment

The overall ROB of included trials was judged to be low or unclear (see Supplementary material online, *Figure S1*), and the quality of reporting improved since 2010 (80% of studies had <3 low-ROB domains pre-2010 vs. 55% post-2010). The 30 (35%) trials reported sufficient and appropriate details of random sequence generation,^21–25,28–32,34–37,41,45,48,50,56,60,61,65,66,72,77,79,82,97,100,103^ and 23 (27%) reported appropriate allocation concealment,^21–25,29–31,34,36,45,50,61,65,68,72,77,79,82,85,96,98,103^ with 24 (28%) reporting sufficient details of outcome assessment blinding.^23–25,28,29,34–36,57,59,60,65,71–74,77,81,82,84,85,98,103^ The 38 (44%) trials were assessed to have low-ROB for incomplete outcome data,^19,25,26,28,29,32–37,40,42,45,49,50,54,59,60,67,69,70,72,73,75,77,79,83,84,86,95,97,98,101,103^ and 62 (73%) had low-ROB for selective reporting.^19,23–25,29,34–36,40–68,70–72,74–78,80,82–89,91,92,94–99,101–103^ GRADE assessments for the clinical event outcomes at short-term follow-up ranged from low to high (*Table 2*), downgrading for imprecision (wide CIs), evidence of publication bias, or substantial statistical heterogeneity.

**Table 2:** Summary of meta-analysis effects of exercise-based cardiac rehabilitation on clinical event outcomes at longest follow-up, short-term follow-up (6–12 months), medium-term follow-up (13–36 months), and long-term follow-up (>36 months)

Outcome follow-up time point	*n* participants	*n* studies	*n* events/participants		RR (95% CI)	Statistical heterogeneity *I*^2^ statistic χ^2^test	GRADE assessment of certainty
			Intervention	Comparator			
*Overall mortality*							
Longest follow-up	16 829	47	919/8608	950/8221	0.96 (0.89–1.04)	0%	
ȃ6–12 months	8823	25	228/4590	242/4233	0.87 (0.73–1.04)	35%	⊕⊕⊕⊝ Moderate^a^
ȃ13–36 months	11 073	16	467/5611	498/5462	0.90 (0.80–1.02)	0%	
ȃ>36 months	3828	11	476/1902	493/1926	0.91 (0.75–1.10)	35%	
*CV mortality*							
Longest follow-up	7762	26	296/3997	382/3765	0.74 (0.64–0.86)***	0%	
ȃ6–12 months	5360	15	109/2799	114/2561	0.88 (0.68–1.14)	0%	⊕⊕⊕⊝ Moderate^a^
ȃ13–36 months	3614	5	199/1861	39/1753	0.77 (0.63 to 0.93)**	5%	
ȃ> 36 months	1392	8	56/690	100/702	0.58 (0.43–0.78)***	0%	
*Fatal and/or non-fatal MI*							
Longest follow-up	14 151	39	383/7181	437/6970	0.82 (0.70–0.96)*	9%	
ȃ6–12 months	7423	22	140/3820	174/3603	0.72 (0.55–0.93)*	7%	⊕⊕⊕⊝ Moderate^b^
ȃ13–36 months	9565	12	264/4830	237/4735	1.07 (0.91–1.27)	0%	
ȃ>36 months	1560	10	65/776	102/784	0.67 (0.50–0.90)**	0%	
*CABG*							
Longest follow-up	5872	29	211/3028	215/2844	0.96 (0.80–1.15)	0%	
ȃ6–12 months	4473	20	125/2324	232/2149	0.99 (0.78–1.27)	0%	⊕⊕⊕⊕ High
ȃ13–36 months	2826	9	123/1413	126/1413	0.97 (0.77–1.23)	0%	
ȃ>36 months	675	4	19/333	29/342	0.66 (0.34–1.27)	18%	
*PCI*							
Longest follow-up	3878	17	171/1960	201/1918	0.84 (0.69–1.02)	0%	
ȃ6–12 months	3465	13	91/1743	104/1722	0.86 (0.63–1.19)	7%	⊕⊕⊕⊝ Moderate^a^
ȃ13–36 months	1983	6	114/996	116/987	0.96 (0.69–1.35)	26%	
ȃ>36 months	567	3	28/281	37/286	0.76 (0.48–1.20)	0%	
*All-cause hospitalization*							
Longest follow-up	7802	21	504/3958	593/3844	0.77 (0.67–0.89)**	32%	
ȃ6–12 months	2030	14	130/1054	209/976	0.58 (0.43–0.77)***	42%*	⊕⊕⊕⊝ Moderate^b^
ȃ13–36 months	5995	9	392/3017	417/2978	0.92 (0.82–1.03)	0%	
*CV hospitalization*							
Longest follow-up	1730	8	152/871	174/859	0.85 (0.67–1.08)	12%	
ȃ6–12 months	1087	6	40/546	42/541	0.8 (0.41–1.59)	53%	⊕⊕⊝⊝ Low^a,c^
ȃ13–36 months	943	3	129/470	141/473	0.92 (0.76–1.12)	0%	

CABG, coronary artery bypass graft; CI, confidence interval; CR, cardiac rehabilitation; CV, cardiovascular; MI, myocardial infarction; PCI, percutaneous coronary intervention; RR, risk ratio.

Downgraded by one level due to imprecision with a wide confidence interval.

Downgraded by one level due to evidence of publication bias.

Downgraded by one level due to substantial heterogeneity.

*P < 0.05.

**P < 0.01.

***P < 0.001.

### Outcomes

A summary of pooled clinical events across all four follow-up time points [longest reported follow-up, short-term (6–12 months), medium-term (13–36 months), and long-term (>36 months)] is presented in *Table 2*. GRADE assessments for certainty of evidence at short-term (6–12 months) follow-up across clinical event outcomes ranged from low-to-high certainty. We downgraded overall mortality, CV mortality, PCI, and CV hospitalization by one level for imprecision, due to wide CIs that overlapped the boundary with no effect. We downgraded MI and all-cause hospitalization by one level due to evidence of publication bias. We downgraded CV hospitalization by an additional level due to evidence of substantial heterogeneity.

#### Mortality

**Figure 2:**
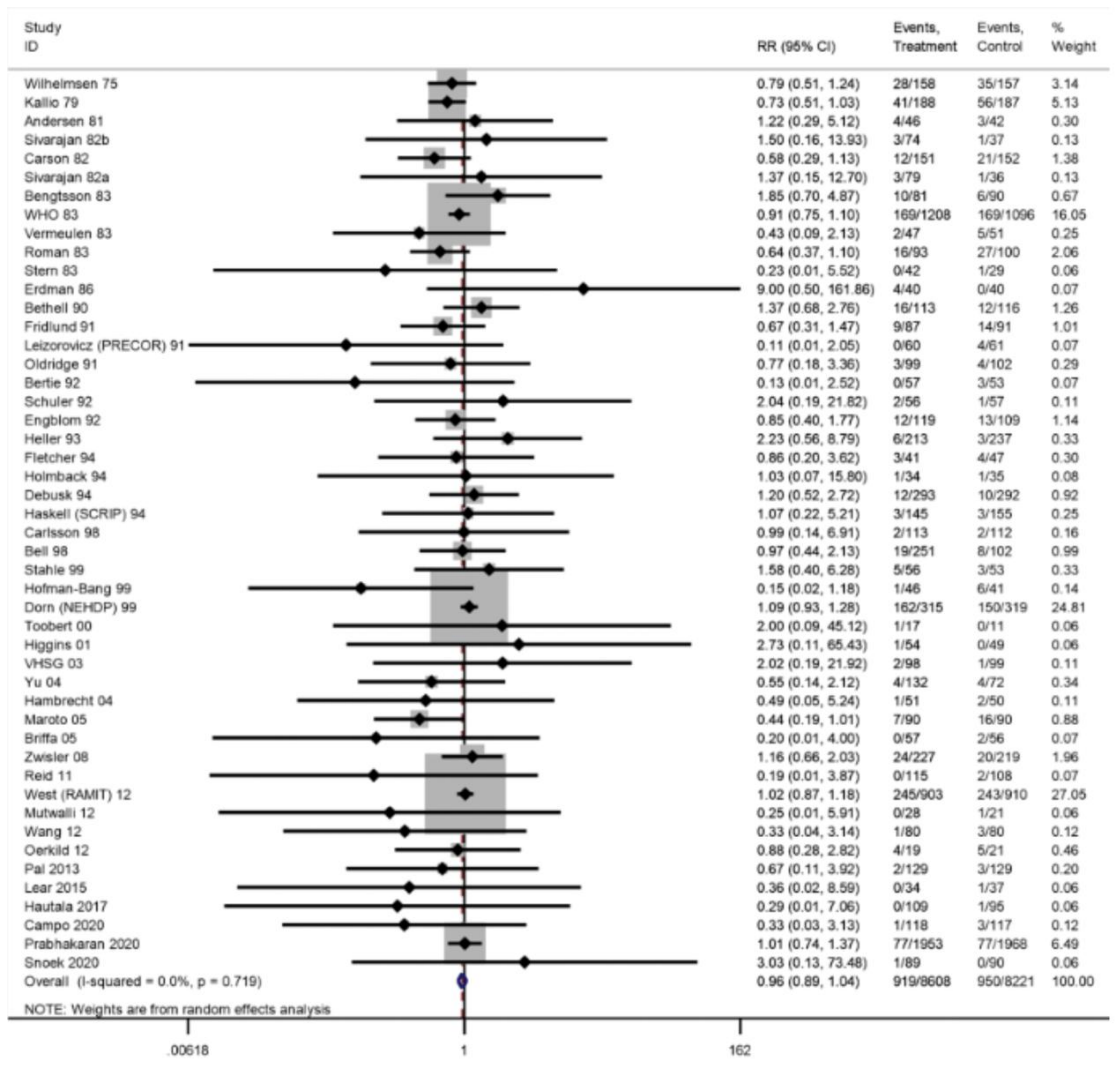
Forest plot: exercise-based cardiac rehabilitation vs. control for overall mortality.

Of the 60 trials (61 comparisons) that reported overall mortality, 13 trials reported zero events in both arms. There was no difference in risk of overall mortality at short-term follow-up (6–12 months; RR: 0.87, 95% CI: 0.73–1.04, *I*^2^ = 0%; moderate certainty evidence) or longest follow-up (47 trials, RR: 0.96, 95% CI: 0.89–1.04, *I*^2^ = 0%; *Figure 2*).

**Figure 3:**
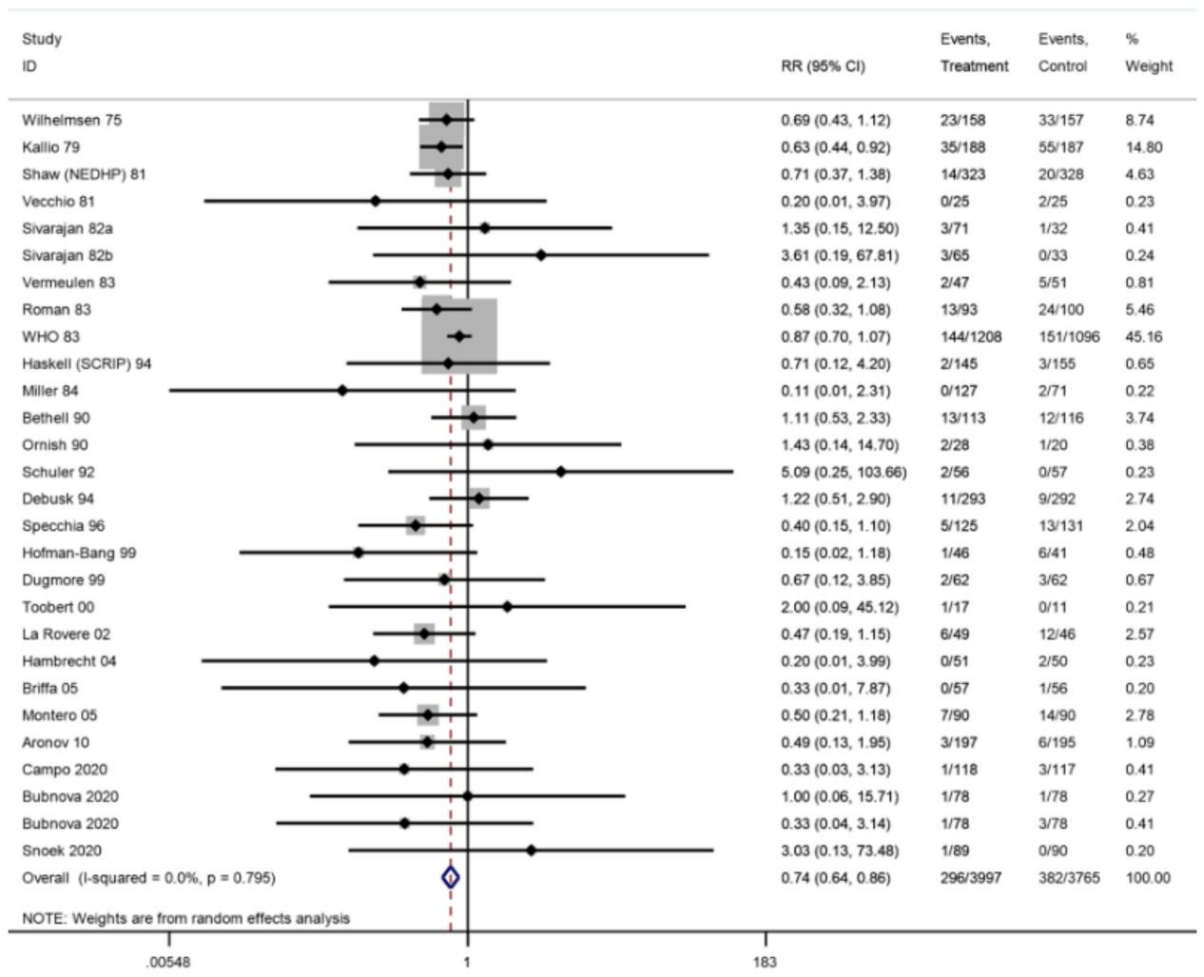
Forest plot: exercise-based cardiac rehabilitation vs. control for cardiovascular mortality.

Across 33 trials (35 comparisons) reporting CV mortality, seven trials reported zero events in both arms. A 26% reduction in risk of CV mortality was seen at longest reported follow-up (26 trials, RR: 0.74, 95% CI: 0.64–0.86, *I*^2^ = 0%; *Figure 3*) with an NNT of 37. At short-term (6–12 months) follow-up, there was no significant difference in CV mortality (RR: 0.88, 95% CI: 0.68–1.14, *I*^2^ = 0%, moderate certainty).

#### Fatal and/or non-fatal MI

**Figure 4:**
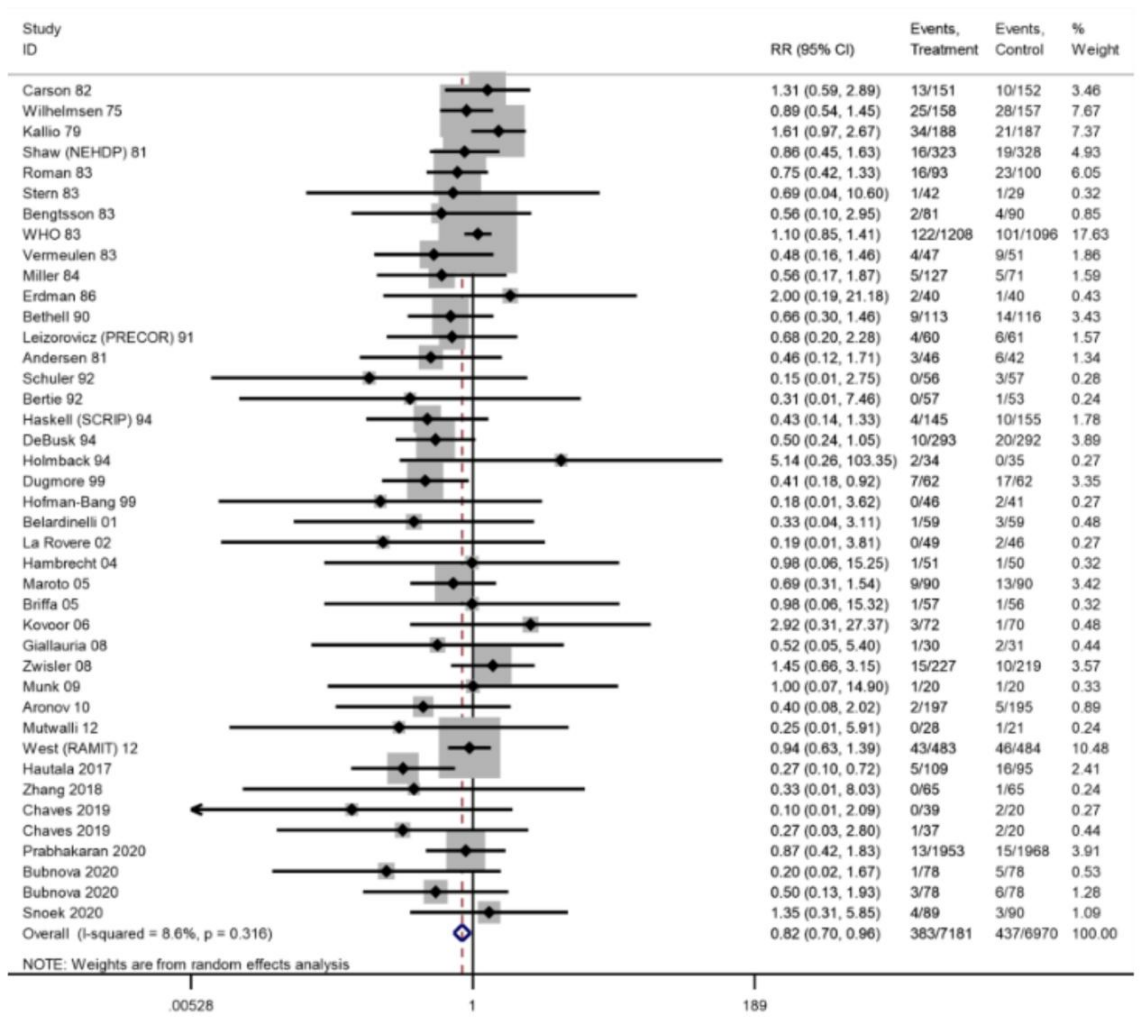
Forest plot: exercise-based cardiac rehabilitation vs. control for myocardial infarction.

Across 42 trials (44 comparisons) reporting fatal and non-fatal MI, three trials reported zero events in both arms. An 18% reduction in risk was shown at longest follow-up (39 trials, RR: 0.82, 95% CI: 0.70–0.96, *I*^2^ = 9%; *Figure 4*) with an NNT of 100. The overall risk was driven by significant reductions in the short-term (6–12 months; RR: 0.72, 95% CI: 0.55– 0.93, *I*^2^ = 7%, high certainty evidence) and long-term (>36 months; RR: 0.67, 95% CI: 0.50–0.90, *I*^2^ = 0%) with no difference in the medium-term follow-up (13–36 months; RR: 1.07, 95% CI: 0.91–1.27, *I*^2^ = 0%).

#### Revascularization events

**Figure 5:**
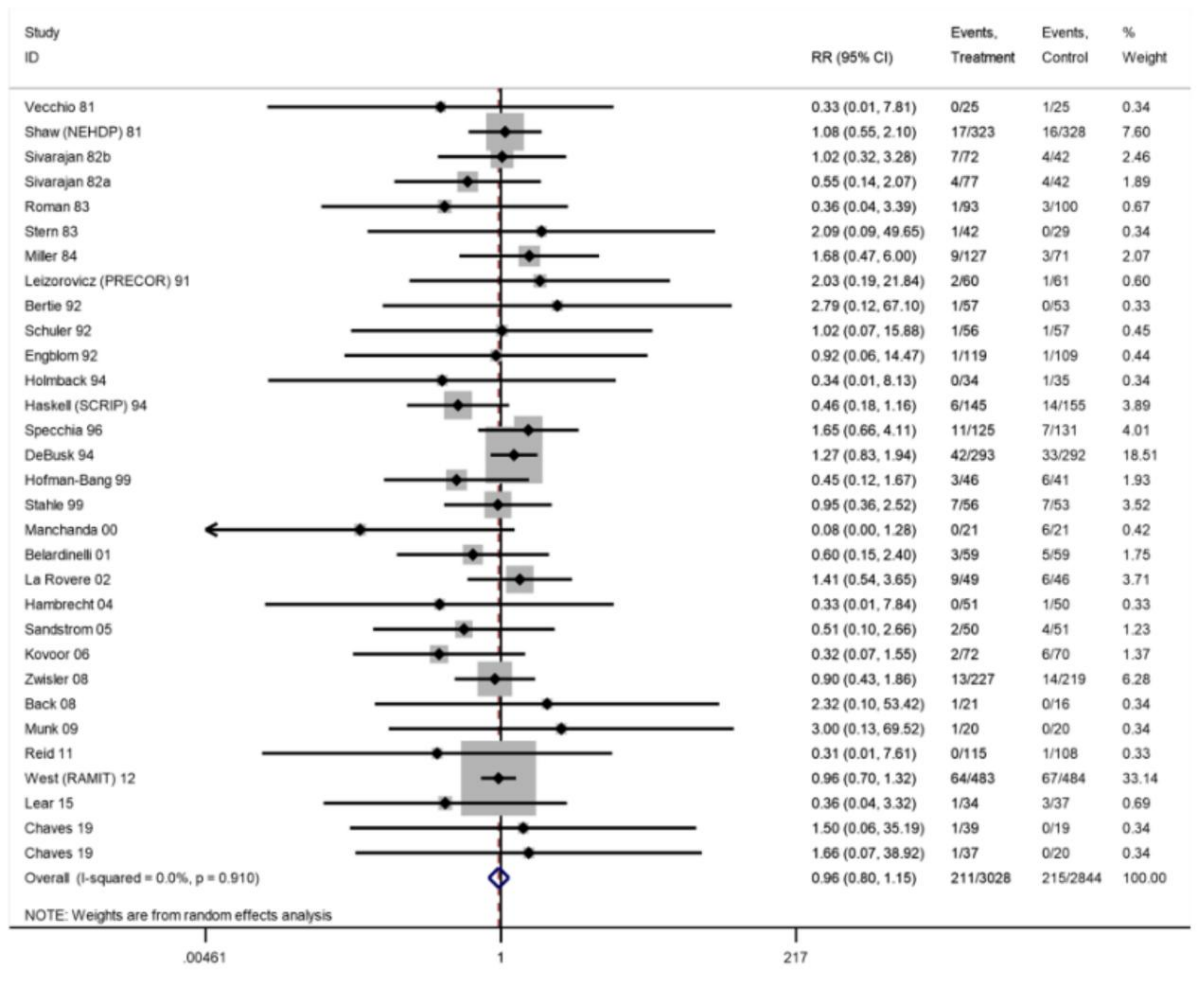
Forest plot: exercise-based cardiac rehabilitation vs. control for coronary artery bypass graft.

**Figure 6:**
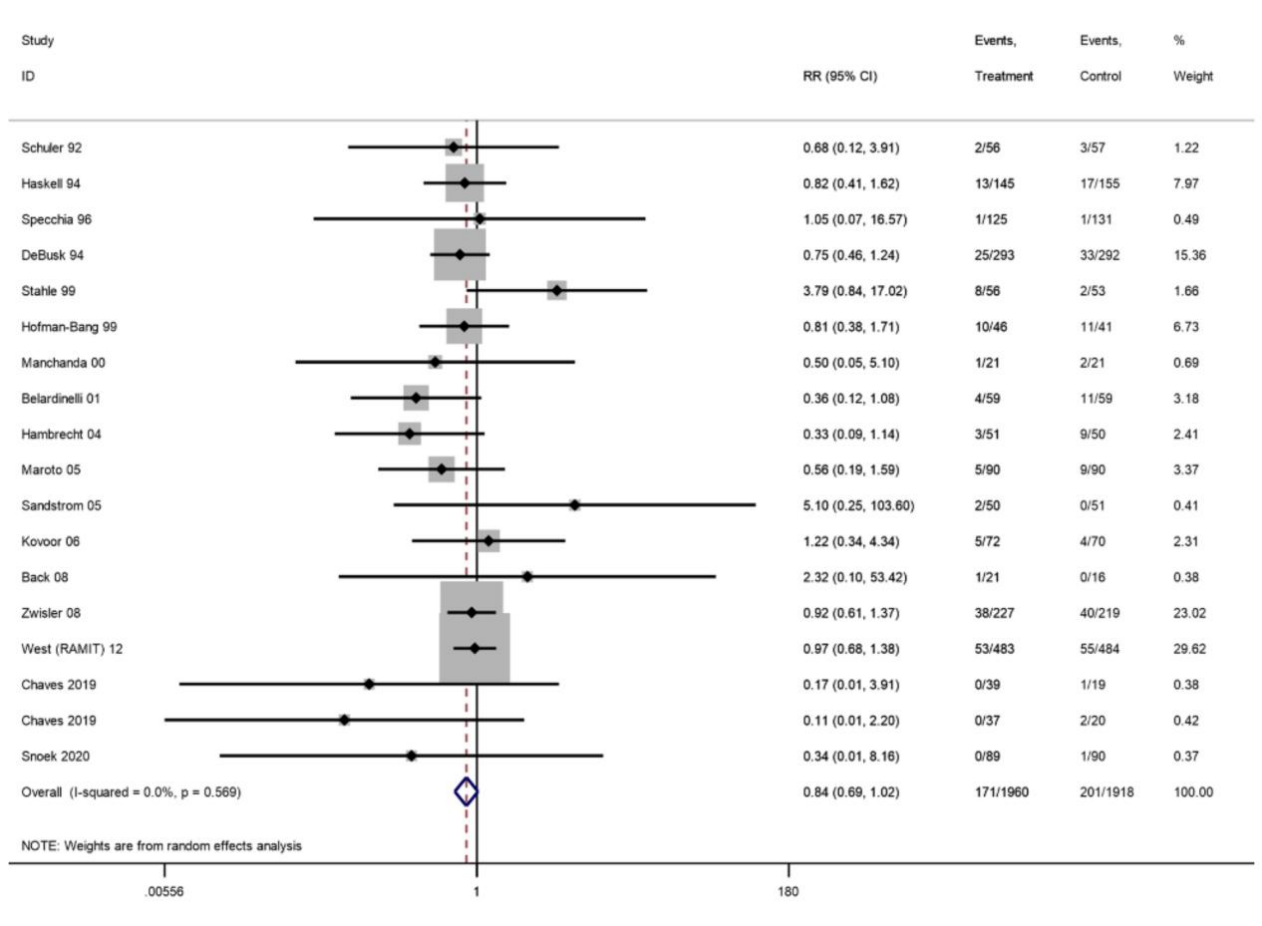
Forest plot: exercise-based cardiac rehabilitation vs. control for percutaneous coronary intervention.

Of 31 trials (33 comparisons) reporting CABG, two trials reported zero events in both arms. There was no difference in risk of CABG at longest follow-up (29 trials, RR: 0.96, 95% CI: 0.80–1.15, *I*^2^ = 0%; *Figure 5*). Of the 20 trials (21 comparisons) reporting PCI, three trials reported zero events in both arms. There was no significant difference in risk of PCI (17 trials, RR: 0.84, 95% CI: 0.69–1.02, *I*^2^ = 0%; *Figure 6*).

#### Hospitalization

**Figure 7:**
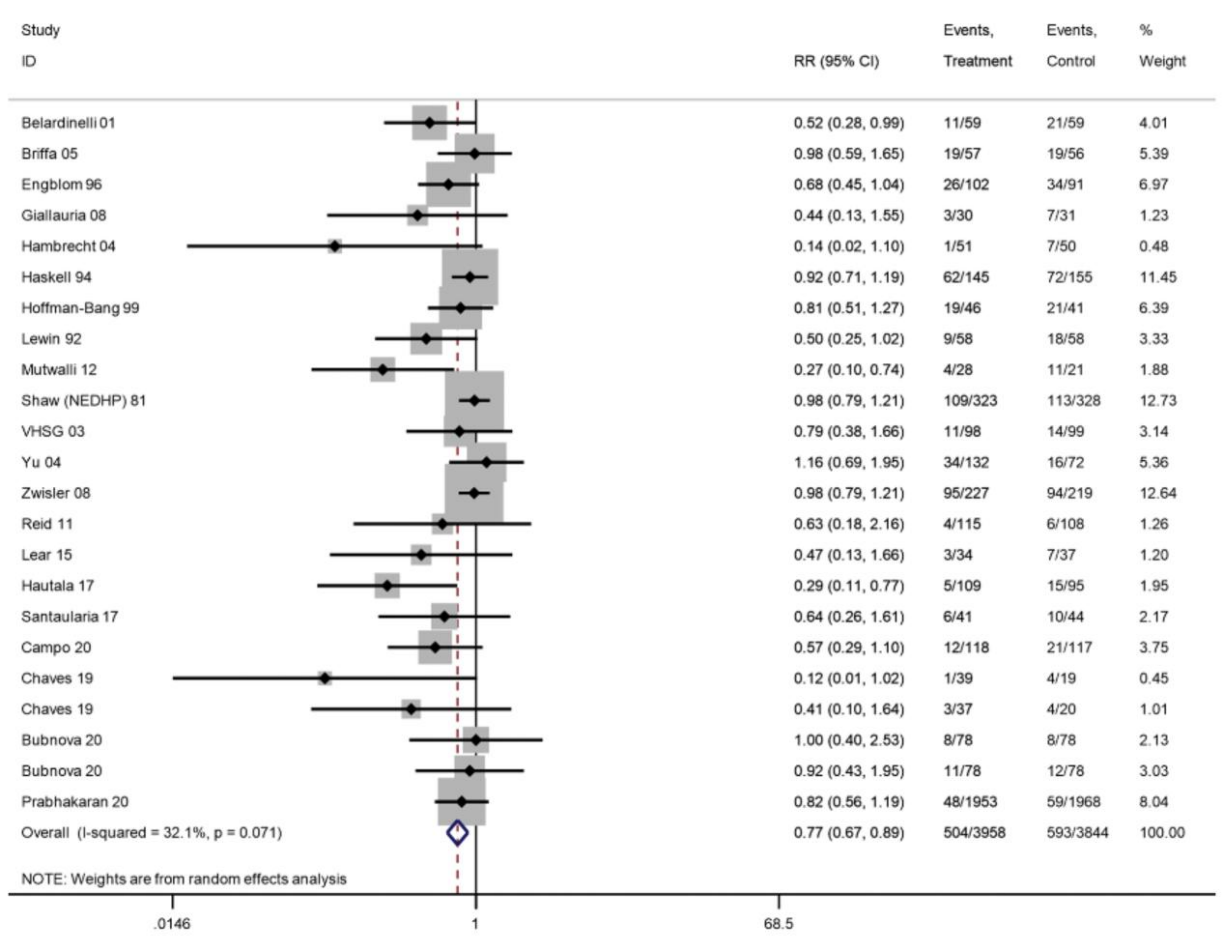
Forest plot: exercise-based cardiac rehabilitation vs. control for overall hospitalization.

**Figure 8:**
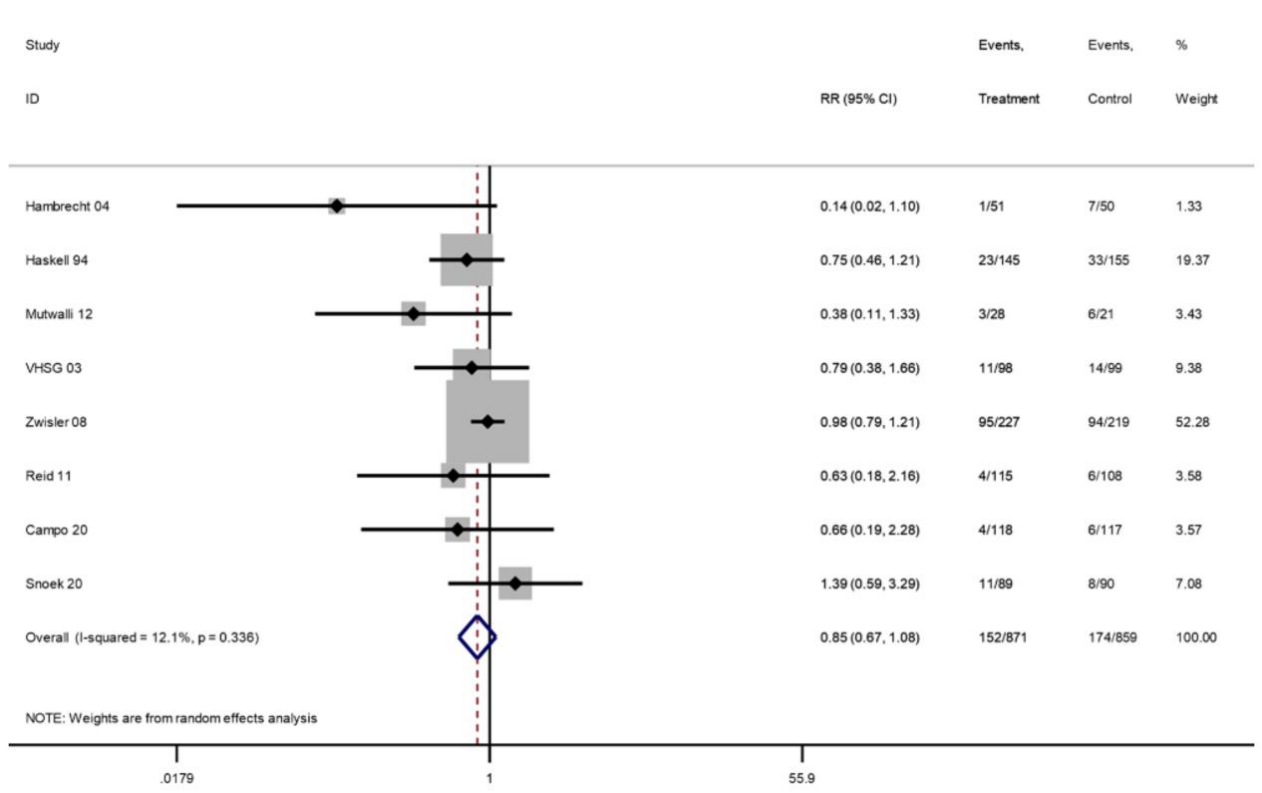
Forest plot: exercise-based cardiac rehabilitation vs. control for cardiovascular hospitalization.

Across 22 trials (24 comparisons) that reported overall hospitalization, one trial reported zero events in both arms. A 23% reduction in overall hospitalization risk with participation in exercise-based CR was shown at the longest follow-up (21 trials, RR: 0.77, 95% CI: 0.67–0.89, *I*^2^ = 32%; *Figure 7*) with an NNT of 37. Nine trials reported CV hospitalizations and one trial reported zero events in both arms. There was no significant difference in CV hospitalization at longest follow-up (eight trials, RR: 0.85, 95% CI: 0.67–1.08, *I*^2^ = 12%; *Figure 8*).

#### Health-related quality of life

**Figure 9:**
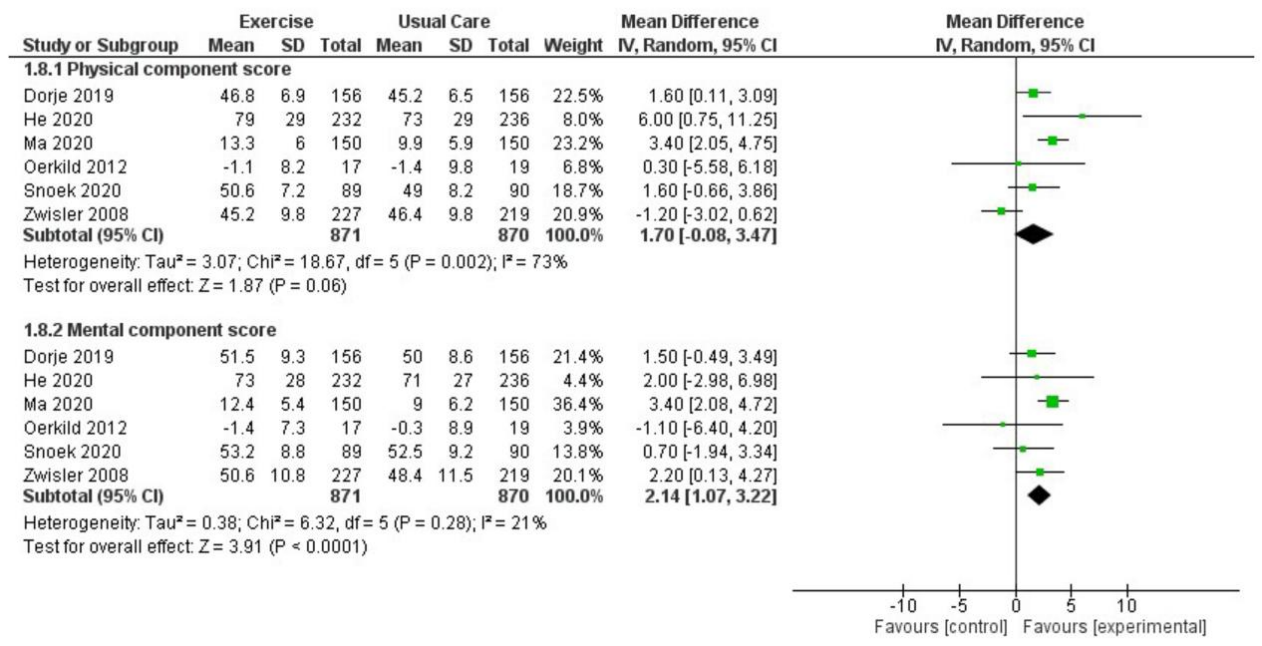
Forest plot: exercise-based cardiac rehabilitation vs. control for health-related quality of life (short-form-36 summary component scores).

**Figure 10:**
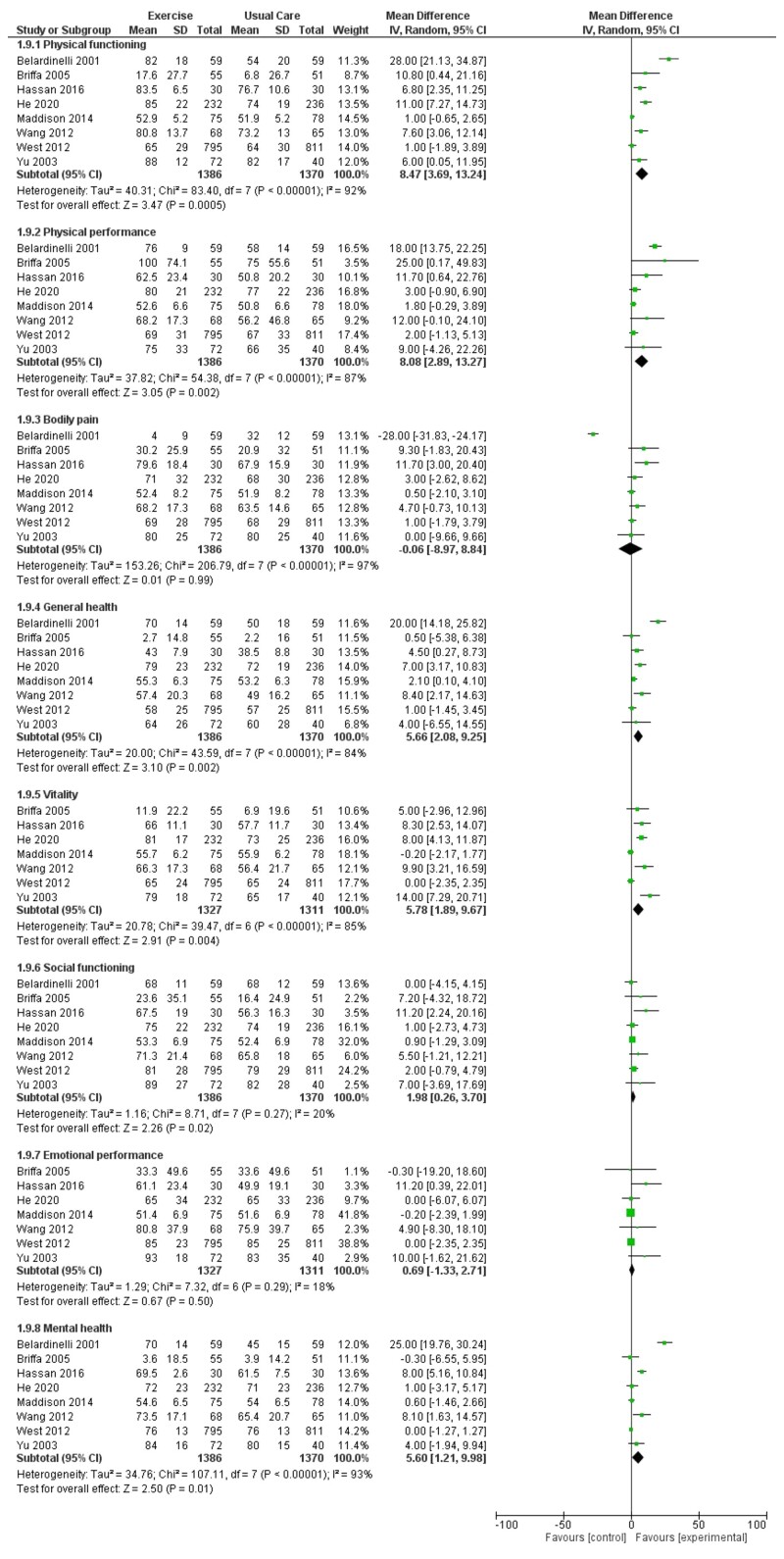
Forest plot: exercise-based cardiac rehabilitation vs. control for health-related quality of life (short-form-36 individual domain scores).

**Figure 11:**
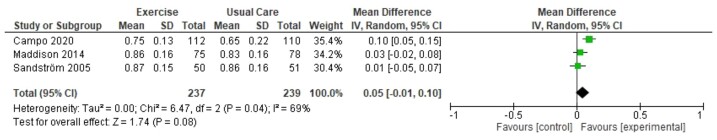
Forest plot: exercise-based cardiac rehabilitation vs. control for health-related quality of life (EQ-5D).

Six trials reported SF-36 summary component scores with up to 12-month follow-up (*Figure 9*). There was evidence of increases in both the mental component score (MD: 2.14, 95% CI: 1.07–3.22, *I*^2^ = 21%) and the physical component score (MD: 1.70, 95% CI: −0.08–3.47, *I*^2^ = 73%) with exercise-based CR. These findings were supported by improvements in selected SF-36 individual domain scores (*Figure 10*) that included physical functioning, physical performance, general health, vitality, social functioning, and mental health. There was no evidence of an improvement in pooled EQ-5D visual analogue scores (VASs; MD 0.05, 95% CI −0.01–0.10, *I*^2^ = 69%; *Figure 11*).

Vote-counting across the 32 trials that assessed HRQoL using a range of validated generic or disease-specific outcome measures confirmed the benefit of CR, with 20 (63%) trials reporting higher levels of HRQoL with exercise-based CR compared with control in one or more subscales and 12 (38%) trials reporting higher levels of HRQoL in >50% of the subscales (see Supplementary material online, *Table S1*).

#### Costs and cost-effectiveness

Only 8 of the 85 studies reported data on healthcare costs of CR with 5 studies reporting overall healthcare costs in both groups (*Table 3*). Total healthcare costs were lower with exercise-based CR than usual care in three studies (mean US$2378,^60^ €1083,^27^ and US$415^102^ less per patient), higher healthcare costs were reported for exercise-based CR than usual care in three studies (mean US$395,^50^ US$4,839,^72^ and US$480^80^ more per patient), and no difference was reported in one study. However, the difference was significant in only one (mean US$2378/patient; *P* < 0.001). Acceptable cost-effectiveness ratios per QALY in favour of exercise-based CR were reported in three trials (US$42,535,^50^ €15,247,^72^ and US$9,200^80^).

**Table 3:** Summary of costs of exercise-based rehabilitation and usual care

	Briffa (2005)	Hambrecht (2004)	Hautala (2017)	Kovoor (2006)/Hall (2002)	Maddison (2014)	Marchionni (2003)	Oldridge (1991/93)	Yu (2004)
Follow-up (months)	12	12	12	12	6	14	12	24
Year of costs (currency)	1998 ($AUD)	NR	NR (€; Euros)	1999 ($AUD)	NR (€; Euros)	2000 ($USD)	1991 ($USD)	2003 ($USD)
*Cost of rehabilitation*								
Mean cost/patient	$694	NR	€299	$394	€127	$5246	$670	NR
Costs considered	Details of costed elements not provided	NR	Estimated according to the average monthly fees in Finnish gyms where individual guidance in exercise training is led by a healthcare professional	staff, assessments, counselling, education, patient travel	NR	NR	space, equipment, staff, literature resources, operating costs, parking, patients costs	NR
*Total healthcare costs*								
Rehabilitation mean cost/patient	$4937	$3708 ± 156	€1944	NR	NR	$17 272	NR	$15 292
Usual care mean cost/patient	$4541	$6086 ± 370	€3027	NR	NR	$12 433	NR	$15 707
Absolute difference in mean cost/patient	$395	−$2378*	−€1083	NR	NR	$4839	$480	−$415
*P*-value for cost difference	0.74	*P* < 0.001	NR	*P* > 0.05 (see below)	NR	NR	NR	*P* > 0.05
Additional healthcare costs considered	Hospitalizations, pharamaceuticals, tests, consultations, rehabilitation, patient expenses, ambulance	Rehospitalizations, revascularization, cycle ergometers, training facilities, and supervising staff	Primary healthcare costs, secondary healthcare costs, occupational healthcare service costs	Phone calls (*P* = 0.10); hospital admissions (*P* = 0.11); gated heart pool scan (*P* = 0.50); exercise stress test (*P* = 0.72); other diagnostics (*P* = 0.37); visits to general practitioner (*P* = 0.61), specialist doctor (*P* = 0.35), or healthcare professional (*P* = 0.31)	NR	NR	Service utilization, physician costs, emergency costs, in-patient days, allied health, other rehabilitation visits	Hospitalizations; revascularizations; private clinic visit; cardiac clinic visits; public non-cardiac visits; casualty visits; drugs
*Cost-effectiveness*								
Rehabilitation mean healthcare benefits	Utility-Based Quality of life–Heart questionnaire: 0.026 (95% CI, 0.013–0.039)	NR	Average change in 15D utility: 0.013	NR	NR	NR	NR	NR
Usual care mean healthcare benefit	Utility 0.010 (95% CI, −0.001 to 0.022)	NR	Average change in 15D utility: −0.012	NR	NR	NR	NR	NR
Incremental mean healthcare benefit	Utility 0.013 (95% CI, NR), *P* = 0.38;+0.009 QALY	NR	0.045 QALY (0.023–0.077)	NR	NR	NR	0.052 QALY (95% CI, 0.007–0.1)	0.06 QALY
Incremental cost-effectiveness ratio/patient	+$42 535 per QALY. Extensive sensitivity analyses reported	NR	−€24 511/QALY	NR	+€15 247 per QALY	NR	+$9200 per QALY	−$650 per QALY

*The healthcare costs within Hambrecht 2004 are reported with $, but currency not specified. NR, not reported; QALY, quality-adjusted life year.

### Small study bias

Egger’s tests and visual inspection of funnel plots indicated there was no evidence of small study bias for overall mortality (Egger’s test: *P* = 0.05; Supplementary material online, *Figure S2*), CV mortality (Egger’s test: *P* = 0.20; Supplementary material online, *Figure S3*), CABG (Egger’s test: *P* = 0.12; Supplementary material online, *Figure S4*), and PCI (Egger’s test: *P* = 0.39; Supplementary material online, *Figure S5*). However, there was evidence of small study bias with funnel plot asymmetry and significant Egger’s tests for MI (Egger’s test: *P* = 0.001; Supplementary material online, *Figure S6*) and all-cause hospitalization (Egger’s test: *P* < 0.001; Supplementary material online, *Figure S7*).

### Meta-regression

There was no evidence of significant differences in treatment effects across patient, intervention, and study characteristics for all clinical event outcomes (see Supplementary material online, *Table S2*).

## Discussion

This updated Cochrane review and meta-analysis of RCTs incorporated data from >23 000 CHD patients and confirmed the benefits of participation in exercise-based CR that include reductions in risk of CV mortality, MI, and all-cause hospitalization at a median follow-up of 12 months (Structured graphical abstract). No significant differences in effect were found across patient case mix, the type or set of CR programme, the dose of exercise prescribed, study sample size, location, length of follow-up, year of publication, and ROB. Reduced hospitalizations are likely to have benefits for both healthcare services as well as for patients in terms of health resource usage and associated costs, and early return home to families and community support networks. Importantly, this updated review demonstrates that the benefits of CR extend across recent trials that are more representative of the modern therapeutic approach in CHD, the expanded CHD population, and low- and middle-income settings (21 trials undertaken in LMICs with 7851 participants), where the prevalence of CHD continues to rise.^104^

Additionally, we found gains in HRQoL with increased scores across six of the eight SF-36 domains, mental component scores, EQ-5D VAS, and synthesis without meta-analysis across 32 trials reporting HRQoL data. Based on the minimally important clinical differences, the increases in the individual domain scores were not clinically important,^105^ but increases in EQ-5D VAS scores could be clinically meaningful.^106^ Minimally important clinical differences for the summary component scores are yet to be published for CHD patients. Although HRQoL is important to patients and improvements have been demonstrated in generic measures, this finding might have been more convincing if a generic measure had been accompanied by the additional use of a CHD disease-specific HRQoL measure. To provide more persuasive evidence, we recommend that future trials consider routinely incorporating both types of HRQoL outcome measures for at least 12 months to delineate which, if any, aspects of HRQoL may yield an improvement. Trial-based economic evaluations showed that CR is a cost-effective use of healthcare resources compared with usual care.

Coronary heart disease is clinically changing from a life-threatening disease to a chronic disease trajectory, as reflected in the terminology of current clinical guidelines on chronic coronary syndromes.^4^ This crucial shift strongly calls for interventions that contribute to improvements in the rehospitalization rate and the well-being and HRQoL of people living with chronic diseases. Thus, this latest Cochrane review of RCTs still reinforces the importance of exercise-based CR as part of integrated CHD care alongside modern invasive and pharmacological therapy.

### Limitations

Our review has a number of potential limitations. First, although we found that the methodological quality and reporting of studies have improved over the last decade and that poor reporting did not appear to alter the review findings, several ROB assessments across trials were judged to be unclear, with many studies inadequately reporting methodologies. Second, this update sought to combine evidence across a range of CHD indications and studies that employed exercise-based CR interventions with varying doses of exercise, delivery settings, and durations of follow-up. However, we applied random-effect meta-analysis to take account of this potential clinical heterogeneity across studies. Furthermore, the GRADE assessment framework also considers heterogeneity in the evidence. For example, the outcomes all-cause mortality, CV mortality, PCI, and CV hospitalization were downgraded in GRADE due to wide CIs that crossed the boundary with no effect. Cardiovascular hospitalization was downgraded due to evidence of statistical heterogeneity (*I*^2^ statistic >50%). Thirdly, while studies reported a prescribed dose of exercise, few, if any, reported the actual level of exercise undertaken by participants. So, we were not able to assess the impact of intervention adherence. Fourth, the number of trials reporting follow-up data beyond 12 months has decreased over the last decade, from 48% (between 2000 and 2009) to 23% (between 2010 and 2020). Consequently, the number of deaths and clinical events reported in several trials were low or zero, and these data were often reported within descriptions of trial loss to follow-up rather than as primary or secondary outcomes, which also means that trials would not have been powered for these outcomes. Additionally, hazard ratios were inconsistently reported across trials; therefore, no analyses using these data were possible. Finally, we also found evidence of reporting bias. For example, although 60 trials reported all-cause mortality, only 33 of these same trials reported CV mortality. Sensitivity analysis of the subgroup group of 16 trials that reported both mortality outcomes (see Supplementary material online, *Figures S8* and *S9*) showed improvements in both pooled overall (RR 0.85, 95% CI: 0.74–0.98) and CV mortality (RR 0.79, 95% CI: 0.68–0.92). This sensitivity analysis is in contrast with our main analysis, showing different effects of exercise-based CR on overall mortality and CV mortality.

## Conclusions

The findings of this latest Cochrane review of 85 RCTs in 23 430 CHD patients confirm the clinical outcome benefits of reduced CV mortality, MI, and hospitalization with participation in exercise-based CR and also provide timely evidence that supports the generalizability of these benefits across patients, in the context of contemporary medical management, and across healthcare settings, including LMICs. This updated review also provides meta-analytic evidence that CR participation improves patient quality of life-based on validated HRQoL data. Our findings reinforce the need to improve access to CR for patients with CHD across the globe.

## Supplementary Material

ehac747_Supplementary_Data

## Data Availability

The data underlying this article will be shared on reasonable request to the corresponding author.

## References

[1] World Health Organization . The top 10 causes of death. https://www.who.int/news-room/fact-sheets/detail/the-top-10-causes-of-death (20 December 2022, date last accessed).

[2] Timmis A , Vardas P, Townsend N, Torbica A, Katus H, De Smedt D, et al European Society of Cardiology: cardiovascular disease statistics 2021. Eur Heart J 2022;43:716–799. 35016208 10.1093/eurheartj/ehab892

[3] Smith SC Jr , Benjamin EJ, Bonow RO, Braun LT, Creager MA, Franklin BA, et al AHA/ACCF secondary prevention and risk reduction therapy for patients with coronary and other atherosclerotic vascular disease: 2011 update: a guideline from the American Heart Association and American College of Cardiology Foundation. J Am Coll Cardiol 2011;58:2432–2446. 22055990 10.1016/j.jacc.2011.10.824

[4] Knuuti J , Wijns W, Saraste A, Capodanno D, Barbato E, Funck-Brenatno C, et al 2019 ESC guidelines for the diagnosis and management of chronic coronary syndromes: the task force for the diagnosis and management of chronic coronary syndromes of the European Society of Cardiology (ESC). Eur Heart J 2020;41:407–477. 31504439 10.1093/eurheartj/ehz425

[5] Anderson L , Thompson DR, Oldridge N, Zwisler AD, Rees K, Martin N, et al Exercise-based cardiac rehabilitation for coronary heart disease. Cochrane Database Syst Rev 2016;2016:CD001800. 10.1002/14651858.CD001800.pub3 PMC6491180 26730878

[6] Xia TL , Huang FY, Peng Y, Huang BT, Pu XB, Yang Y, et al Efficacy of different types of exercise-based cardiac rehabilitation on coronary heart disease: a network meta-analysis. J Gen Intern Med 2018;33:2201–2209. 30215179 10.1007/s11606-018-4636-y PMC6258639

[7] Salzwedel A , Jensen K, Rauch B, Doherty P, Metzendorf MI, Hackbusch M, et al Effectiveness of comprehensive cardiac rehabilitation in coronary artery disease patients treated according to contemporary evidence based medicine: update of the cardiac rehabilitation outcome study (CROS-II). Eur J Prev Cardiol 2020;27:1756–1774. 32089005 10.1177/2047487320905719 PMC7564293

[8] Powell R , McGregor G, Ennis S, Kimani PK, Underwood M. Is exercise-based cardiac rehabilitation effective? A systematic review and meta-analysis to re-examine the evidence. BMJ Open 2018;8:e019656. 10.1136/bmjopen-2017-019656 PMC5857699 29540415

[9] McGregor G , Powell R, Kimani P, Underwood M. Does contemporary exercise-based cardiac rehabilitation improve quality of life for people with coronary artery disease? A systematic review and meta-analysis. BMJ Open 2020;10:e036089. 10.1136/bmjopen-2019-036089 PMC7282413 32513887

[10] Higgins JPT , Thomas J, Chandler J, Cumpston M, Li T, Page MJ, et al *Cochrane handbook for systematic reviews of intervention* [Version 6.2] Updated February 2021. Cochrane, 2021. www.training.cochrane.org/handbook

[11] Page MJ , McKenzie JE, Bossuyt PM, Boutron I, Hoffmann TC, Mulrow CD, et al The PRISMA 2020 statement: an updated guideline for reporting systematic reviews. BMJ 2021;372:n71. 33782057 10.1136/bmj.n71 PMC8005924

[12] Campbell M , McKenzie JE, Sowden A, Katikreddi SV, Brennan SE, Ellis S, et al Synthesis without meta-analysis (SWiM) in systematic reviews: reporting guideline. BMJ 2020;368:I6890. 10.1136/bmj.l6890 PMC7190266 31948937

[13] Higgins JPT , Altman DG, Stene JAC. Assessing risk of bias in included studies. In: Higgins JPT, Green S (eds.), *Cochrane Handbook for Systematic Reviews of Interventions Version 5.1.0* Updated March 2011. The Cochrane Collaboration, 2011. www.handbook.cochrane.org

[14] *GRADEpro GTD: GRADEpro Guideline Development Tool*. McMaster University and Evidence Prime, 2021. www.gradepro.org

[15] Schünemann H , Brożek J, Guyatt G, Oxman A. GRADE Handbook for Grading Quality of Evidence and Strength of Recommendations. The GRADE Working Group; 2013. http://www.guidelinedevelopment.org/handbook (20 December 2022, date last accessed).

[16] Wen L , Badgett R, Cornell J. Number needed to treat: a descriptor for weighing therapeutic options. Am J Health Syst Pharm 2005;62:2031–2036. 16174840 10.2146/ajhp040558

[17] Egger M , Davey Smith G, Schneider M, Minder C. Bias in meta-analysis detected by a simple graphical test. BMJ 1997;315:629–634. 9310563 10.1136/bmj.315.7109.629 PMC2127453

[18] The World Bank . *World Bank Country and Lending Groups. Country Classification*. https://datahelpdesk.worldbank.org/knowledgebase/articles/906519-world-bank-country-and-lending-groups (20 December 2022, date last accessed).

[19] Aronov D , Bubnova M, Iosseliani D, Orekhov A. Clinical efficacy of а medical centre- and home-based cardiac rehabilitation program for patients with coronary heart disease after coronary bypass graft surgery. Arch Med Res 2019;50:122–32. 10.1016/j.arcmed.2019.07.007 31495389

[20] Bubnova MG , Aronov DM. Clinical effects of a one-year cardiac rehabilitation program using physical training after myocardial infarction in patients of working age with different rehabilitation potentials. Cardiovasc Ther Prev 2019;18:27–37.

[21] Bubnova MG , Aronov DM. Physical rehabilitation after acute myocardial infarction: focus on body weight. Russ J Cardiol 2020;25:3867.

[22] Byrkjeland R , Njerve IU, Anderssen S, Arnesen H, Seljeflot I, Solheim S. Effects of exercise training on HbA _1c_ and VO _2peak_ in patients with type 2 diabetes and coronary artery disease: A randomised clinical trial. Diab Vasc Dis Res 2015;12:325–33. 26092822 10.1177/1479164115590552

[23] Campo G , Tonet E, Chiaranda G, et al Exercise intervention improves quality of life in older adults after myocardial infarction: randomised clinical trial. Heart 2020;106:1658–64. 32144189 10.1136/heartjnl-2019-316349

[24] Chaves GSS , Lima de Melo Ghisi G, Britto RR, Grace SL. Maintenance of gains, morbidity, and mortality at 1 year following cardiac rehabilitation in a middle-income country: a wait-list control crossover trial. J Am Heart Assoc 2019;8. 10.1161/JAHA.118.011228 PMC6405675 30764702

[25] Dorje T , Zhao G, Tso K, et al Smartphone and social media-based cardiac rehabilitation and secondary prevention in China (SMART-CR/SP): a parallel-group, single-blind, randomised controlled trial. Lancet Digit Health 2019;1:e363–74. 33323210 10.1016/S2589-7500(19)30151-7

[26] Hassan AM , Nahas NG. Efficacy of cardiac rehabilitation after percutaneous coronary intervention. Int J Pharmtech Res 2016;9:134–41.

[27] Hautala AJ , Kiviniemi AM, Mäkikallio T, et al Economic evaluation of exercise-based cardiac rehabilitation in patients with a recent acute coronary syndrome. Scand J Med Sci Sports 2017;27:1395–403. 27541076 10.1111/sms.12738

[28] He C-J , Zhu C-Y, Zhu Y-J, et al Effect of exercise-based cardiac rehabilitation on clinical outcomes in patients with myocardial infarction in the absence of obstructive coronary artery disease (MINOCA). Int J Cardiol 2020;315:9–14. 32416201 10.1016/j.ijcard.2020.05.019

[29] Lear SA , Singer J, Banner-Lukaris D. Improving access to cardiac rehabilitation using the internet: a randomized trial. Stud Health Technol Inform 2015;209:58–66. 25980706

[30] Ma L , Deng L, Yu H. The effects of a comprehensive rehabilitation and intensive education program on anxiety, depression, quality of life, and major adverse cardiac and cerebrovascular events in unprotected left main coronary artery disease patients who underwent coronary artery bypass grafting. Ir J Med Sci (1971 -) 2020;189:477–88. 10.1007/s11845-019-02129-x 31758523

[31] Pal A , Srivastava N, Narain VS, Agrawal GG. Effect of yogic intervention on the autonomic nervous system in the patients with coronary artery disease: a randomized controlled trial. East Mediterr Health J 2013;19:453–8. 24617124

[32] Pomeshkina SA , Loktionova EB, Bezzubova VA, Arkhipova NV, Borovik IV, Barbarash OL. The comparative analysis of the influence of the supervised exercise training and home-based exercise training on the psychological status of the following coronary artery bypass grafting. Voprosy kurortologii, fizioterapii i lechebnoi fizicheskoi kul'tury 2017;94:10. 10.17116/kurort201794610-17 29388927

[33] Pomeshkina SA , Barbarash OL, Pomeshkin EV. Exercise training and erectile dysfunction in patients after coronary artery bypass grafting. Terapevticheskii arkhiv 2019;91:16–20. 10.26442/00403660.2019.09.000149 32598809

[34] Prabhakaran D , Chandrasekaran AM, Singh Ka, et al Yoga-based cardiac rehabilitation after acute myocardial infarction. J Am Coll Cardiol 2020;75:1551–61. 32241371 10.1016/j.jacc.2020.01.050 PMC7132532

[35] Santaularia N , Caminal J, Arnau A, et al The efficacy of a supervised exercise training programme on readmission rates in patients with myocardial ischemia: results from a randomised controlled trial. Eur J Cardiovasc Nurs 2016;16:201–12. 27162127 10.1177/1474515116648801

[36] Snoek JA , Prescott EI, van der Velde AE, et al Effectiveness of home-based mobile guided cardiac rehabilitation as alternative strategy for nonparticipation in clinic-based cardiac rehabilitation among elderly patients in Europe. JAMA Cardiol 2021;6:463. 33112363 10.1001/jamacardio.2020.5218 PMC7593879

[37] Sun P , Li Y, Song C. Long-term effects of exercise rehabilitation on risk factors in elderly patients with stable coronary artery disease. Chin J Geriatric Heart Brain Vessel Dis 2016;5:491–5.

[38] Uddin J , Joshi VL, Moniruzzaman M, et al Effect of home-based cardiac rehabilitation in a lower-middle income country. J Cardiopulm Rehabil Prev 2020;40:29–34. 31714393 10.1097/HCR.0000000000000471

[39] Xu Y , Feng Y, Su P, Li Y, Li C, Qiao J. Impact of exercise rehabilitation on cardiac function in coronary artery disease patients after percutaneous coronary intervention. Chin Circ J 2017;32:326–30.

[40] Zhang Y , Cao H, Jiang P, Tang H. Cardiac rehabilitation in acute myocardial infarction patients after percutaneous coronary intervention. Medicine 2018;97:e9785. 29465559 10.1097/MD.0000000000009785 PMC5841979

[41] Andersen GS , Christiansen P, Madsen S, Schmidt G. The value of regular, supervised physical training after acute myocardial infarction [Vaerdien af regelmaessig og overvåget fysisk traening efter akut myokardieinfarkt. Ugeskrift for Laeger 1981;143:2952–5. 7330986

[42] Aronov DM , Krasnitskij VB, Bubnova MG. Efficacy of physical training and analysis of lipid-lowering therapy in patients with ischemic heart disease after acute coronary incidents. Ration Pharmacother Cardiol 2010;6:9–19.

[43] Bäck M , Wennerblom B, Wittboldt S, Cider Å. Effects of high frequency exercise in patients before and after elective percutaneous coronary intervention. Eur J Cardiovasc Nurs 2008;7:307–13. 18372218 10.1016/j.ejcnurse.2008.02.001

[44] Belardinelli R , Paolini I, Cianci G, Piva R, Georgiou D, Purcaro A. Exercise training intervention after coronary angioplasty: the ETICA trial. J Am Coll Cardiol 2001;37:1891–900. 11401128 10.1016/s0735-1097(01)01236-0

[45] Bell JM . *A Comparison of a Multi-Disciplinary Home Based Cardiac Rehabilitation Programme with Comprehensive Conventional Rehabilitation in Post-Myocardial Infarction Patients*. UK: University of London, 1998.

[46] Bengtsson K . Rehabilitation after myocardial infarction. Scand J Rehabil Med 1983;15:1–9. 6828827

[47] Bertie J , King A, Reed N, Marshall AJ, Ricketts C. Benefits and weaknesses of a cardiac rehabilitation programme. J R Coll Physicians Lond 1992;26:147–51. 1588521 PMC5375519

[48] Bethell HJ , Mullee MA. A controlled trial of community based coronary rehabilitation. Heart 1990;64:370–5. 10.1136/hrt.64.6.370 PMC1224812 2271343

[49] Bettencourt N , Dias C, Mateus P. Impact of cardiac rehabilitation on quality of life and depression after acute coronary syndrome [Impacto da reabilitacao cardiaca na qualidade-de-vida e sintomatologia depressiva apos sindroma coronaria aguda. Rev Port Cardiol 2005;24:687–96. 16041965

[50] Briffa TG , Eckermann SD, Griffiths AD, et al Cost-effectiveness of rehabilitation after an acute coronary event: a randomised controlled trial. Med J Aust 2005;183:450–5. 16274344 10.5694/j.1326-5377.2005.tb07121.x

[51] Carlsson R . Serum cholesterol, lifestyle, working capacity and quality of life in patients with coronary artery disease. Experiences from a hospital-based secondary prevention programme. Scand Cardiovasc J 2009;32:1–20. 10.1080/140174398427956-1 9802147

[52] Carson P , Phillips R, Lloyd M. Exercise after myocardial infarction: a controlled trial. J R Coll Physicians Lond 1982;16:147–51. 7050369 PMC5377720

[53] DeBusk RF . A case-management system for coronary risk factor modification after acute myocardial infarction. Ann Intern Med 1994;120:721. 8147544 10.7326/0003-4819-120-9-199405010-00001

[54] Dugmore LD , Tipson RJ, Phillips MH, et al Changes in cardiorespiratory fitness, psychological wellbeing, quality of life, and vocational status following a 12 month cardiac exercise rehabilitation programme. Heart 1999;81:359–66. 10092561 10.1136/hrt.81.4.359 PMC1729018

[55] Engblom E , Korpilahti K, Hämäläinen H, Puukka P, Rönnemaa T. Effects of five years of cardiac rehabilitation after coronary artery bypass grafting on coronary risk factors. Am J Cardiol 1996;78:1428–31. 8970420 10.1016/s0002-9149(96)00629-7

[56] Erdman RAM , Duivenvoorden HJ, Verhage F, Kazemier M, Hugenholtz PG. Predictability of beneficial effects in cardiac rehabilitation. J Cardiopulm Rehabil 1986;6:206–13.

[57] Fletcher BJ , Dunbar SB, Felner JM, et al Exercise testing and training in physically disabled men with clinical evidence of coronary artery disease. Am J Cardiol 1994;73:170–4. 8296738 10.1016/0002-9149(94)90209-7

[58] Fridlund B , Högstedt B, Lidell E, Larsson PA. Recovery after myocardial infarction. Scand J Caring Sci 1991;5:23–32. 2011669 10.1111/j.1471-6712.1991.tb00078.x

[59] Giallauria F , Cirillo P, Lucci R, et al Left ventricular remodelling in patients with moderate systolic dysfunction after myocardial infarction: favourable effects of exercise training and predictive role of *N* -terminal pro-brain natriuretic peptide. Eur J Prev Cardiol Prev R 2008;15:113–18. 10.1097/HJR.0b013e3282f00990 18277196

[60] Hambrecht R , Walther C, Möbius-Winkler S, et al Percutaneous coronary angioplasty compared with exercise training in patients with stable coronary artery disease. Circulation 2004;109:1371–8. 15007010 10.1161/01.CIR.0000121360.31954.1F

[61] Haskell WL , Alderman EL, Fair JM, et al Effects of intensive multiple risk factor reduction on coronary atherosclerosis and clinical cardiac events in men and women with coronary artery disease. The Stanford Coronary Risk Intervention Project (SCRIP). Circulation 1994;89:975–90. 8124838 10.1161/01.cir.89.3.975

[62] Heller RF , Knapp JC, Valenti LA, Dobson AJ. Secondary prevention after acute myocardial infarction. Am J Cardiol 1993;72:759–62. 8105673 10.1016/0002-9149(93)91058-p

[63] Higgins HC , Hayes RL, McKenna KT. Rehabilitation outcomes following percutaneous coronary interventions (PCI). Patient Educ Couns 2001;43:219–30. 11384820 10.1016/s0738-3991(00)00164-6

[64] Hofman-Bang C . Two-year results of a controlled study of residential rehabilitation for patients treated with percutaneous transluminal coronary angioplasty. A randomized study of a multifactorial programme. Eur Heart J 1999;20:1465–74. 10493845 10.1053/euhj.1999.1544

[65] Holmbäck AM , Säwe U, Fagher B. Training after myocardial infarction: Lack of long-term effects on physical capacity and psychological variables. Arch Phys Med Rehabil 1994;75:551–4. 8185448

[66] Houle J , Doyon O, Vadeboncoeur N, Turbide G, Diaz A, Poirier P. Effectiveness of a pedometer-based program using a socio-cognitive intervention on physical activity and quality of life in a setting of cardiac rehabilitation. Can J Cardiol 2012;28:27–32. 22177854 10.1016/j.cjca.2011.09.020

[67] Kallio V , Hämäläinen H, Hakkila J, Luurila OJ. Reduction in sudden deaths by a multifactorial intervention programme after acute myocardial infarction. Lancet 1979;314:1091–4. 10.1016/s0140-6736(79)92502-9 91836

[68] Kovoor P , Lee AKY, Carrozzi F, et al Return to full normal activities including work at two weeks after acute myocardial infarction. Am J Cardiol 2006;97:952–8. 16563893 10.1016/j.amjcard.2005.10.040

[69] La Rovere MT , Bersano C, Gnemmi M, Specchia G, Schwartz PJ. Exercise-induced increase in baroreflex sensitivity predicts improved prognosis after myocardial infarction. Circulation 2002;106:945–9. 12186798 10.1161/01.cir.0000027565.12764.e1

[70] Leizoroviez A , Saint-Pierre A, Vasselon C, Boissel JP. Comparison of a rehabilitation programme, a counselling programme and usual care after an acute myocardial infarction: results of a long-term randomized trial. Eur Heart J 1991;12:612–6. 1874262 10.1093/oxfordjournals.eurheartj.a059948

[71] Lewin B , Robertson IR, Cay EL, Irving JB, Campbell M. Effects of self-help post-myocardial-infarction rehabilitation on psychological adjustment and use of health services. Lancet 1992;339:1036–40. 1349062 10.1016/0140-6736(92)90547-g

[72] Maddison R , Pfaeffli L, Whittaker R et al A mobile phone intervention increases physical activity in people with cardiovascular disease: Results from the HEART randomized controlled trial. Eur J Prev Cardiol 2014;22:701–9. 24817694 10.1177/2047487314535076

[73] Manchanda SC , Narang R, Reddy KS. Retardation of coronary atherosclerosis with yoga lifestyle intervention. J Assoc Physicians India 2000;48:687–94. 11273502

[74] Marchionni N , Fattirolli F, Fumagalli S, et al Improved exercise tolerance and quality of life with cardiac rehabilitation of older patients after myocardial infarction. Circulation 2003;107:2201–6. 12707240 10.1161/01.CIR.0000066322.21016.4A

[75] Maroto MJ , Artigao Ramirez R, Morales Duran MD, de Pablo Zarzosa C, Abraira V. Cardiac rehabilitation in patients with myocardial infarction: a 10-year follow-up study. Rev Esp Cardiol 2005;58:1181–7. 16238986

[76] Miller NH , Haskell WL, Berra K, DeBusk RF. Home versus group exercise training for increasing functional capacity after myocardial infarction. Circulation 1984;70:645–9. 6478567 10.1161/01.cir.70.4.645

[77] Munk PS , Staal EM, Butt N, Isaksen K, Larsen AI. High-intensity interval training may reduce in-stent restenosis following percutaneous coronary intervention with stent implantation. Am Heart J 2009;158:734–41. 19853690 10.1016/j.ahj.2009.08.021

[78] Mutwalli HA , Fallows SJ, Arnous AA, Zamzami MS. Randomized controlled evaluation shows the effectiveness of a home-based cardiac rehabilitation program. Saudi Med J 2012;33:152–9. 22327755

[79] Oerkild B , Frederiksen M, Hansen JF, Prescott E. Home-based cardiac rehabilitation is an attractive alternative to no cardiac rehabilitation for elderly patients with coronary heart disease: results from a randomised clinical trial. BMJ Open 2012;2:e001820. 10.1136/bmjopen-2012-001820 PMC3533030 23253876

[80] Oldridge N , Guyatt G, Jones N, et al Effects on quality of life with comprehensive rehabilitation after acute myocardial infarction. Am J Cardiol 1991;67:1084–9. 2024598 10.1016/0002-9149(91)90870-q

[81] Ornish D , Brown SE, Billings JH, et al Can lifestyle changes reverse coronary heart disease? Lancet 1990;336:129–33. 1973470 10.1016/0140-6736(90)91656-u

[82] Reid RD , Morrin LI, Beaton LJ, et al Randomized trial of an internet-based computer-tailored expert system for physical activity in patients with heart disease. Eur J Prev Cardiol 2011;19:1357–64. 21903744 10.1177/1741826711422988

[83] Román O , Gutierrez M, Luksic I, et al Cardiac rehabilitation after acute myocardial infarction. Cardiology 1983;70:223–31. 6640562 10.1159/000173598

[84] Sandström L , Ståhle A. Rehabilitation of elderly with coronary heart disease – improvement in quality of life at a low cost. Adv Physiother 2009;7:60–6.

[85] Schuler G , Hambrecht R, Schlierf G, et al Regular physical exercise and low-fat diet. Effects on progression of coronary artery disease. Circulation 1992;86:1–11. 1617762 10.1161/01.cir.86.1.1

[86] Seki E , Watanabe Y, Sunayama S, et al Effects of phase III cardiac rehabilitation programs on health-related quality of life in elderly patients with coronary artery disease. Juntendo Cardiac Rehabilitation Program (J-CARP). Circ J 2003;67:73–7. 12520156 10.1253/circj.67.73

[87] Seki E , Watanabe Y, Shimada K, et al Effects of a phase III cardiac rehabilitation program on physical status and lipid profiles in elderly patients with coronary artery disease Juntendo Cardiac Rehabilitation Program (J-CARP). Circ J 2008;72:1230–4. 18654005 10.1253/circj.72.1230

[88] Shaw LW , Oberman A, Barnes G, et al Effects of a prescribed supervised exercise program on mortality and cardiovascular morbidity in patients after a myocardial infarction. Am J Cardiol 1981;48:39–46. 6972693 10.1016/0002-9149(81)90570-1

[89] Sivarajan ES , Bruce RA, Lindskog BD, Almes MJ, Belanger L, Green B. Treadmill test responses to an early exercise program after myocardial infarction: a randomized study. Circulation 1982;65:1420–8. 7074797 10.1161/01.cir.65.7.1420

[90] Specchia G , De Servi S, Scirè A, et al Interaction between exercise training and ejection fraction in predicting prognosis after a first myocardial infarction. Circulation 1996;94:978–82. 8790035 10.1161/01.cir.94.5.978

[91] Ståhle A . Improved physical fitness and quality of life following training of elderly patients after acute coronary events. A 1 year follow-up randomized controlled study. Eur Heart J 1999;20:1475–84. 10493846 10.1053/euhj.1999.1581

[92] Stern MJ , Gorman PA, Kaslow L. The group counseling v exercise therapy study. A controlled intervention with subjects following myocardial infarction. Arch Intern Med 1983;143:1719–25. 6615094

[93] Toobert DJ , Glasgow RE, Radcliffe JL. Physiologic and related behavioral outcomes from the women’s lifestyle heart trial. Ann Behav Med 2000;22:1–9. 10892523 10.1007/BF02895162

[94] Vecchio C , Cobelli F, Opasich C, Assandri J, Poggi G, Griffo R. Early functional evaluation and physical rehabilitation in patients with wide myocardial infarction [Valutazione funzionale precoce e riabilitazione fisica nei pazienti con infarto miocardico esteso. G Ital Cardiol 1981;11:419–29. 6456960

[95] Vermeulen A , Lie KI, Durrer D. Effects of cardiac rehabilitation after myocardial infarction: changes in coronary risk factors and long-term prognosis. Am Heart J 1983;105:798–801. 6846123 10.1016/0002-8703(83)90243-0

[96] Otterstad JE . Influence on lifestyle measures and five-year coronary risk by a comprehensive lifestyle intervention programme in patients with coronary heart disease. Eur J Cardiov Prev R 2016;10:429–37. 10.1097/01.hjr.0000107024.38316.6a 14671465

[97] Wang W , Chair SY, Thompson DR, Twinn SF. Effects of home-based rehabilitation on health-related quality of life and psychological status in Chinese patients recovering from acute myocardial infarction. Heart Lung 2012;41:15–25. 21974926 10.1016/j.hrtlng.2011.05.005

[98] West RR , Jones DA, Henderson AH. Rehabilitation after myocardial infarction trial (RAMIT): multi-centre randomised controlled trial of comprehensive cardiac rehabilitation in patients following acute myocardial infarction. Heart 2012;98:637–44. 22194152 10.1136/heartjnl-2011-300302

[99] World Health Organization. *Rehabilitation and Comprehensive Secondary Prevention after Acute Myocardial Infarction*. EURO Reports and Studies, 1983.

[100] Wilhelmsen L , Sanne H, Elmfeldt D, Grimby G, Tibblin G, Wedel H. A controlled trial of physical training after myocardial infarction. Prev Med 1975;4:491–508. 1208362 10.1016/0091-7435(75)90035-3

[101] Yu C-M , Li LS-W, Ho HH, Lau C-P. Long-term changes in exercise capacity, quality of life, body anthropometry, and lipid profiles after a cardiac rehabilitation program in obese patients with coronary heart disease. Am J Cardiol 2003;91:321–5. 12565088 10.1016/s0002-9149(02)03159-4

[102] Yu C-M , Lau C-P, Chau J, et al A short course of cardiac rehabilitation program is highly cost effective in improving long-term quality of life in patients with recent myocardial infarction or percutaneous coronary intervention. Arch Phys Med Rehabil 2004;85:1915–22. 15605326 10.1016/j.apmr.2004.05.010

[103] Zwisler A-DO , Soja AMB, Rasmussen S, et al Hospital-based comprehensive cardiac rehabilitation versus usual care among patients with congestive heart failure, ischemic heart disease, or high risk of ischemic heart disease: 12-Month results of a randomized clinical trial. Am Heart J 2008;155:1106–13. 18513526 10.1016/j.ahj.2007.12.033

[104] Prabhakaran D , Anand S, Watkins D, Gaziano T, Wi Y, Mbanya JC, et al Cardiovascular, respiratory, and related disorders: key messages from Disease Control Priorities, third edition. Lancet 2018;391:1224–1236. 29108723 10.1016/S0140-6736(17)32471-6 PMC5996970

[105] Wyrwich KW , Spertus JA, Kroenke K, Tierney WM, Babu AN, Wolinsky FD. Clinically important differences in health status for patients with heart disease: an expert consensus panel report. Am Heart J 2004;147:615–622. 15077075 10.1016/j.ahj.2003.10.039

[106] Briggs A , LLoyd A, Pickard SS. Minimal clinically important difference in Eq-5D: we can calculate it, but does that mean we should? www.ispor.org/docs/default-source/presentations/1066.pdf?%20;sfvrsn=25feffd6_1 (20 December 2022, date last accessed).

