# Supplementary material for: Exercise-based cardiac rehabilitation for coronary heart disease: a meta-analysis
Source: Eur Heart J. 2023 Jan 2;44(6):452–69. doi: 10.1093/eurheartj/ehac747 (PMC9902155; doi:10.1093/eurheartj/ehac747)
Supplement: ehac747_Supplementary_Data [file ehac747_supplementary_data.pdf]

Supplementary files

1) Search strategy..2

2) Complete reference list for included studies...12

3) Supplementary Figure 1: Risk of bias assessment..25

4) Supplementary Table 1: Summary of health-related quality of life (HRQoL) scores at follow-up...26

5) Supplementary Figure 2: Funnel plot – exercise-based CR vs control for all-cause mortality..33

6) Supplementary Figure 3: Funnel plot – exercise-based CR vs control for cardiovascular mortality..34

7) Supplementary Figure 4: Funnel plot – exercise-based CR vs control for CABG.............35

8) Supplementary Figure 5: Funnel plot – exercise-based CR vs control for PCI..................36

9) Supplementary Figure 6: Funnel plot – exercise-based CR vs control for MI....................37

10) Supplementary Figure 7: Funnel plot – exercise-based CR vs control for all-cause hospitalisation...38

11) Supplementary Table 2: Results for univariate meta-regression for primary clinical outcomes RR (95% CI) 39

12) Supplementary Figure 8: Forest plot – exercise-based CR vs control for all-cause mortality in trials that report both all-cause mortality and cardiovascular mortality data........40

13) Supplementary Figure 9: Forest plot – exercise-based CR vs control for CV mortality in trials that report both all-cause mortality and cardiovascular mortality data..........................41

Supplementary file 1: Search strategy

CENTRAL

#1 MeSH descriptor: [Myocardial Ischemia] explode all trees

#2 (myocard* near isch*mi*):ti,ab,kw

#3 isch*mi* near heart:ti,ab,kw

#4 MeSH descriptor: [Coronary Artery Bypass] explode all trees

#5 myocard* near infarct*:ti,ab,kw

#6 heart near infarct*:ti,ab,kw

#7 angina:ti,ab,kw

#8 coronary near (disease* or bypass or thrombo* or angioplast*):ti,ab,kw

#9 MeSH descriptor: [Percutaneous Coronary Intervention] explode all trees

#10 (percutaneous next coronary near/2 (interven* or revascular*))

#11 MeSH descriptor: [Angioplasty] explode all trees

#12 angioplast*

#13 ((coronary or arterial) near/4 dilat*)

#14 endoluminal next repair*

#15 MeSH descriptor: [Stents] explode all trees

#16 stent*

#17 pci or ptca

#18 MeSH descriptor: [Atherectomy] explode all trees

#19 atherectom*

#20 acute next coronary next syndrom*

#21 (NSTEMI or STEMI)

#22 ACS

#23 #1 or #2 or #3 or #4 or #5 or #6 or #7 or #8 or #9 or #10 or #11 or #12 or #13 or #14 or #15 or #16 or #17 or #18 or #19 or #20

#24 #1 or #2 or #3 or #4 or #5 or #6 or #7 or #8 or #9 or #10 or #11 or #12 or #13 or #14 or #15 or #16 or #17 or #18 or #19 or #20 or #21 or #22

#25 MeSH descriptor: [Exercise Therapy] explode all trees

#26 MeSH descriptor: [Sports] explode all trees

#27 MeSH descriptor: [Physical Exertion] explode all trees

#28 rehabilitat*:ti,ab,kw

#29 (physical* near (fit* or train* or therap* or activit*)):ti,ab,kw

#30 MeSH descriptor: [Exercise] explode all trees

#31 (train*) near (strength* or aerobic* or exercise*):ti,ab,kw

#32 ((exercise* or fitness) near/3 (treatment or intervent* or program*)):ti,ab,kw

#33 MeSH descriptor: [Rehabilitation] explode all trees

#34 kinesiotherap*:ti,ab,kw

#35 MeSH descriptor: [Physical Education and Training] explode all trees

#36 MeSH descriptor: [Patient Education as Topic] this term only

#37 (patient* near/5 educat*)

#38 ((lifestyle or life-style) near/5 (interven* or program* or treatment*))

#39 MeSH descriptor: [Self Care] this term only

#40 (self near/5 (manag* or care or motivate*))

#41 MeSH descriptor: [Psychotherapy] explode all trees

#42 psychotherap*

#43 (psycholog* near/5 intervent*)

#44 MeSH descriptor: [Counseling] this term only

#45 (counselling or counseling)

#46 ((behavior* or behaviour*) near/5 (modify or modificat* or therap* or change))

#47 (psycho-educat* or psychoeducat*)

#48 (motivat* near/5 (intervention or interv*))

#49 MeSH descriptor: [Health Education] this term only

#50 (health near/5 educat*)

#51 (psychosocial or psycho-social)

#52 (cognitive near/2 behav*)

#53 #25 or #26 or #27 or #28 or #29 or #30 or #31 or #32 or #33 or #34 or #35 or #36 or #37 or #38 or #39 or #40 or #41 or #42 or #43 or #44 or #45 or #46 or #47 or #48 or #49 or #50 or #51 or #52

#54 #23 and #53 in Trials

#55 #24 and #53 in Trials

#56 #55 not #54 in Trials

#57 #23 and #53 with Publication Year from 2014 to 2020, in Trials

#58 #56 or #57

MEDLINE OVID

1 exp Myocardial Ischemia/

2 (myocard* adj5 (ischaemia or ischemia)).tw.

3 (isch?emi* adj5 heart).tw.

4 exp Coronary Artery Bypass/

5 (myocard* adj5 infarct*).tw.

6 (heart adj5 infarct*).tw.

7 angina.tw.

8 (coronary adj5 (disease* or bypass or thrombo* or angioplast*)).tw.

9 exp Percutaneous Coronary Intervention/

10 (percutaneous coronary adj2 (interven* or revascular*)).tw.

11 exp Angioplasty/

12 angioplast*.tw.

13 ((coronary or arterial) adj4 dilat*).tw.

14 endoluminal repair*.tw.

15 exp Stents/

16 stent*.tw.

17 (pci or ptca).tw.

18 exp Atherectomy/

19 atherectom*.tw.

20 acute coronary syndrom*.tw.

21 (NSTEMI or STEMI).tw.

22 ACS.tw.

23 1 or 2 or 3 or 4 or 5 or 6 or 7 or 8 or 9 or 10 or 11 or 12 or 13 or 14 or 15 or 16 or 17 or 18 or 19 or 20

24 1 or 2 or 3 or 4 or 5 or 6 or 7 or 8 or 9 or 10 or 11 or 12 or 13 or 14 or 15 or 16 or 17 or 18 or 19 or 20 or 21 or 22

25 exp Exercise Therapy/

26 Sports/

27 Physical Exertion/

28 rehabilitat*.mp.

29 (physical* adj5 (fit* or train* or therap* or activit*)).mp.

30 exp Exercise/

31 (train* adj5 (strength* or aerobic* or exercise*)).tw.

32 ((exercise* or fitness) adj3 (treatment or intervent* or program*)).tw.

33 exp Rehabilitation/

34 kinesiotherap*.tw.

35 "Physical Education and Training"/

36 Patient Education as Topic/

37 (patient* adj5 educat*).tw.

38 ((lifestyle or life-style) adj5 (interven* or program* or treatment*)).tw.

39 Self Care/

40 (self adj5 (manag* or care or motivate*)).tw.

41 exp Psychotherapy/

42 psychotherap*.tw.

43 (psycholog* adj5 intervent*).tw.

44 Counseling/

45 (counselling or counseling).tw.

46 ((behavior* or behaviour*) adj5 (modify or modificat* or therap* or change)).tw.

47 (psycho-educat* or psychoeducat*).tw.

48 (motivat* adj5 (intervention or interv*)).tw.

49 Health Education/

50 (health adj5 educat*).tw.

51 (psychosocial or psycho-social).tw.

52 (cognitive adj2 behav*).tw.

53 25 or 26 or 27 or 28 or 29 or 30 or 31 or 32 or 33 or 34 or 35 or 36 or 37 or 38 or 39 or 40 or 41 or 42 or 43 or 44 or 45 or 46 or 47 or 48 or 49 or 50 or 51 or 52

54 randomized controlled trial.pt.

55 controlled clinical trial.pt.

56 randomized.ab.

57 placebo.ab.

58 drug therapy.fs.

59 randomly.ab.

60 trial.ab.

61 groups.ab.

62 54 or 55 or 56 or 57 or 58 or 59 or 60 or 61

63 exp animals/ not humans.sh.

64 62 not 63

65 23 and 53 and 64

66 24 and 53 and 64

67 limit 65 to ed=20140702-20200901

68 66 not 65

69 67 or 68

Embase OVID

1 exp Heart Muscle Ischemia/

2 (myocard* adj5 (ischaemia or ischemia)).tw.

3 (isch?emi* adj5 heart).tw.

4 exp Coronary Artery Bypass Graft/

5 (myocard* adj5 infarct*).tw.

6 (heart adj5 infarct*).tw.

7 angina.tw.

8 (coronary adj5 (disease* or bypass or thrombo* or angioplast*)).tw.

9 exp percutaneous coronary intervention/

10 (percutaneous coronary adj2 (interven* or revascular*)).tw.

11 exp angioplasty/

12 angioplast*.tw.

13 ((coronary or arterial) adj4 dilat*).tw.

14 endoluminal repair*.tw.

15 exp stent/

16 stent*.tw.

17 (pci or ptca).tw.

18 exp atherectomy/

19 atherectom*.tw.

20 acute coronary syndrom*.tw.

21 (NSTEMI or STEMI).tw.

22 ACS.tw.

23 1 or 2 or 3 or 4 or 5 or 6 or 7 or 8 or 9 or 10 or 11 or 12 or 13 or 14 or 15 or 16 or 17 or 18 or 19 or 20

24 1 or 2 or 3 or 4 or 5 or 6 or 7 or 8 or 9 or 10 or 11 or 12 or 13 or 14 or 15 or 16 or 17 or 18 or 19 or 20 or 21 or 22

25 exp Kinesiotherapy/

26 Sport/

27 rehabilitat*.mp.

28 (physical* adj5 (fit* or train* or therap* or activit*)).mp.

29 exp Exercise/

30 (train* adj5 (strength* or aerobic* or exercise*)).tw.

31 ((exercise* or fitness) adj3 (treatment or intervent* or program*)).tw.

32 exp Rehabilitation/

33 kinesiotherap*.tw.

34 Physical Education/

35 patient education/

36 (patient* adj5 educat*).tw.

37 ((lifestyle or life-style) adj5 (interven* or program* or treatment*)).tw.

38 self care/

39 (self adj5 (manag* or care or motivate*)).tw.

40 exp psychotherapy/

41 psychotherap*.tw.

42 (psycholog* adj5 intervent*).tw.

43 counseling/

44 (counselling or counseling).tw.

45 ((behavior* or behaviour*) adj5 (modify or modificat* or therap* or change)).tw.

46 (psycho-educat* or psychoeducat*).tw.

47 (motivat* adj5 (intervention or interv*)).tw.

48 health education/

49 (health adj5 educat*).tw.

50 (psychosocial or psycho-social).tw.

51 (cognitive adj2 behav*).tw.

52 25 or 26 or 27 or 28 or 29 or 30 or 31 or 32 or 33 or 34 or 35 or 36 or 37 or 38 or 39 or 40 or 41 or 42 or 43 or 44 or 45 or 46 or 47 or 48 or 49 or 50 or 51

53 random$.tw.

54 factorial$.tw.

55 crossover$.tw.

56 cross over$.tw.

57 cross-over$.tw.

58 placebo$.tw.

59 (doubl$ adj blind$).tw.

60 (singl$ adj blind$).tw.

61 assign$.tw.

62 allocat$.tw.

63 volunteer$.tw.

64 crossover procedure/

65 double blind procedure/

66 randomized controlled trial/

67 single blind procedure/

68 53 or 54 or 55 or 56 or 57 or 58 or 59 or 60 or 61 or 62 or 63 or 64 or 65 or 66 or 67

69 (animal/ or nonhuman/) not human/

70 68 not 69

71 23 and 52 and 70

72 24 and 52 and 70

73 limit 71 to dd=20140702-20200901

74 72 not 71

75 73 or 74

76 limit 75 to embase

CINAHL

S75 S73 OR S74

S74 S22 AND S52 AND S70 Limiters - Published Date: 20140701-20200831

S73 S72 NOT S71

S72 S23 AND S52 AND S70

S71 S22 AND S52 AND S70

S70 S53 OR S54 OR S55 OR S56 OR S57 OR S58 OR S59 OR S60 OR S61 OR S62 OR S63 OR S64 OR S65 OR S66 OR S67 OR S68 OR S69

S69 TX cross-over*

S68 TX crossover*

S67 TX volunteer*

S66 (MH "Crossover Design")

S65 TX allocat*

S64 TX control*

S63 TX assign*

S62 TX placebo*

S61 (MH "Placebos")

S60 TX random*

S59 TX (doubl* N1 mask*)

S58 TX (singl* N1 mask*)

S57 TX (doubl* N1 blind*)

S56 TX (singl* N1 blind*)

S55 TX (clinic* N1 trial?)

S54 PT clinical trial

S53 (MH "Clinical Trials+")

S52 S24 OR S25 OR S26 OR S27 OR S28 OR S29 OR S30 OR S31 OR S32 OR S33 OR S34 OR S35 OR S36 OR S37 OR S38 OR S39 OR S40 OR S41 OR S42 OR S43 OR S44 OR S45 OR S46 OR S47 OR S48 OR S49 OR S50 OR S51

S51 (cognitive N2 behav*)

S50 (psychosocial or psycho-social)

S49 (health N5 educat*)

S48 (MH "Health Education")

S47 (motivat* N5 (intervention or interv*))

S46 (psycho-educat* or psychoeducat*)

S45 ((behavior* or behaviour*) N5 (modify or modificat* or therap* or change))

S44 (counselling or counseling)

S43 (MH "Counseling")

S42 (psycholog* N5 intervent*)

S41 psychotherap*

S40 (MH "Psychotherapy+")

S39 (self N5 (manag* or care or motivate*))

S38 (MH "Self Care")

S37 ((lifestyle or life-style) N5 (interven* or program* or treatment*))

S36 (patient* N5 educat*)

S35 (MH "Patient Education")

S34 (MH "Physical Education and Training")

S33 kinesiotherap*

S32 (MH "Rehabilitation+")

S31 ((exercise* or fitness) N3 (treatment or intervent* or program*))

S30 (train* N5 (strength* or aerobic* or exercise*))

S29 (MH "Exercise+")

S28 (physical* N5 (fit* or train* or therap* or activit*))

S27 rehabilitat*

S26 (MH "Physical Activity")

S25 (MH "Sports")

S24 (MH "Therapeutic Exercise+")

S23 S1 OR S2 OR S3 OR S4 OR S5 OR S6 OR S7 OR S8 OR S9 OR S10 OR S11 OR S12 OR S13 OR S14 OR S15 OR S16 OR S17 OR S18 OR S19 OR S20 OR S21 OR S22

S22 S1 OR S2 OR S3 OR S4 OR S5 OR S6 OR S7 OR S8 OR S9 OR S10 OR S11 OR S12 OR S13 OR S14 OR S15 OR S16 OR S17 OR S18 OR S19

S21 ACS

S20 (NSTEMI or STEMI)

S19 "acute coronary syndrom*"

S18 atherectom*

S17 (MH "Atherectomy+")

S16 pci or ptca

S15 stent*

S14 (MH "Stents+")

S13 "endoluminal repair*"

S12 ((coronary or arterial) N4 dilat*)

S11 angioplast*

S10 (MH "Angioplasty+")

S9 (percutaneous coronary N2 (interven* or revascular*))

S8 (coronary N5 (disease* or bypass or thrombo* or angioplast*))

S7 angina

S6 (heart N5 infarct*)

S5 (myocard* N5 infarct*)

S4 (MH "Coronary Artery Bypass+")

S3 (isch?emi* N5 heart)

S2 (myocard* N5 (ischaemia or ischemia))

S1 (MH "Myocardial Ischemia+")

Web of Science

#16 #15 OR #14

#15 #11 AND #10 AND #8 Indexes=SCI-EXPANDED, CPCI-S Timespan=2014-2020

#14 #13 NOT #12

#13 #11 AND #10 AND #9

#12 #11 AND #10 AND #8

#11 TS=(random* or blind* or allocat* or assign* or trial* or placebo* or crossover* or cross-over*)

#10 TS=((rehab* or educat*))

#9 #7 OR #6 OR #5 OR #4 OR #3 OR #2 OR #1

#8 #5 OR #4 OR #3 OR #2 OR #1

#7 TS=ACS

#6 TS=(NSTEMI or STEMI)

#5 TS=(PCI or percutaneous or angioplast* or "endoluminal repair*" or stent* or atherectom* or "acute coronary syndrom*")

#4 TS=((angina or cardiac* or PTCA or CABG))

#3 TS=(((heart) SAME (infarct* or isch?emia or failure or attack)))

#2 TS=(((coronary* or heart*) SAME (by?pass or disease*)))

#1 TS=(((myocard*) SAME (isch?emia or infarct* or revasculari?*)))

ClinicalTrials.gov

Condition or disease: Coronary Heart Disease

Intervention/treatment: Cardiac Rehabilitation

Study type: Interventional studies (Clinical Trials)

WHO ICTRP

Condition: Coronary Heart Disease

Intervention: Exercise OR Cardiac rehabilitation

Supplementary file 2: Complete reference list for included studies

(primary [denoted by *] and associated supplementary references for each included study)

Anderson 1981
*Andersen GS, Christiansen P, Madsen S, Schmidt G. The value of regular, supervised physical training after acute myocardial infarction [Vaerdien af regelmaessig og overvåget fysisk traening efter akut myokardieinfarkt]. *Ugeskrift for Laeger.* 1981;143(45):2952-5.

Aronov 2010
*Aronov DM, Krasnitskij VB, Bubnova MG. Efficacy of physical training and analysis of lipid-lowering therapy in patients with ischemic heart disease after acute coronary incidents. *Rational Pharmacotherapy Cardiology*. 2010;6(1):9-19. doi: 10.20996/1819-6446-2010-6-1-9-19

Aronov 2019
*Aronov D, Bubnova M, Iosseliani D, Orekhov A. Clinical efficacy of a medical centre- and home-based cardiac rehabilitation program for patients with coronary heart disease after coronary bypass graft surgery. *Arch Med Res.* 2019;50(3):122-32. doi: 10.1016/j.arcmed.2019.07.007

Back 2008
*Bäck M, Wennerblom B, Wittboldt S, Cider A. Effects of high frequency exercise in patients before and after elective percutaneous coronary intervention. *Eur J Cardiovasc Nurs.* 2008;7(4):307-13. doi: 10.1016/j.ejcnurse.2008.02.001

Belardinelli 2001
*Belardinelli R, Paolini I, Cianci G, Piva R, Georgiou D, Purcaro A. Exercise training intervention after coronary angioplasty: the ETICA Trial. *J Am Coll Cardiol.* 2001;37(7):1891-900. doi: 10.1016/s0735-1097(01)01236-0.

Bell 1998
*Bell JM. *A comparison of a multi-disciplinary home based cardiac rehabilitation programme with comprehensive conventional rehabilitation in post-myocardial infarction patients.* Thesis. University of London; 1998.

Bengtsson 1983
*Bengtsson K. Rehabilitation after myocardial infarction. *Scand J Rehabil Med.* 1983;15(1):1-9.

Bertie 1992
*Bertie J, King A, Reed N, Marshall AJ, Ricketts C. Benefits and weaknesses of a cardiac rehabilitation programme. *J R Coll Physicians Lond.* 1992;26(2):147-51.

Bethell 1990
*Bethell HJ, Mullee MA. A controlled trial of community based coronary rehabilitation. *Br Heart J.* 1990;64(6):370-5. doi: 10.1136/hrt.64.6.370.

Bettencourt 2005
*Bettencourt N, Dias C, Mateus P, et al. Impact of cardiac rehabilitation on quality of life and depression after acute coronary syndrome [Impacto da reabilitacao cardiaca na qualidade-de-vida e sintomatologia depressiva apos sindroma coronaria aguda]. *Rev Port Cardiol.* 2005;24:687-96.

Briffa 2005

*Briffa TG, Eckermann SD, Griffiths AD, et al. Cost-effectiveness of rehabilitation after an acute coronary event: a randomised controlled trial. *Med J Aust.* 2005;183:450-5. doi: 10.5694/j.1326-5377.2005.tb07121.x.

Bubnova 2019
*Bubnova MG, Aronov DM. Clinical effects of a one-year cardiac rehabilitation program using physical training after myocardial infarction in patients of working age with different rehabilitation potentials. *Cardiovascular Therapy and Prevention.* 2019;18(5):27-37. doi: 10.15829/1728-8800-2019-5-27-37.

Bubnova 2020
*Bubnova MG, Aronov DM. Physical rehabilitation after acute myocardial infarction: focus on body weight. *Russian Journal of Cardiology.* 2020;25(5):3867. doi: 10.15829/1560-4071-2020-3867.

Byrkjeland 2015
*Byrkjeland R, Njerve IU, Anderssen S, Arnesen H, Seljeflot I, Solheim S. Effects of exercise training on HbA1c and VO2peak in patients with type 2 diabetes and coronary artery disease: a randomised clinical trial. *Diabetes & Vascular Disease Research.* 2015;12(5):325-33. doi: 10.1177/1479164115590552

Byrkjeland R, Stensaeth K, Anderssen S, et al. Effects of exercise training on carotidintima-media thickness in patients with type 2 diabetes and coronary artery disease. Influence of carotid plaques. *Cardiovasc Diabetol.* 2016;15:13. doi: 10.1186/s12933-016-0336-2.

Njerve IU, Byrkjeland R, Arnesen H, Akra S, Solheim S, Seljeflot I. Effects of 12 months exercise intervention on adipose tissue expression of chemokines in patients with type 2 diabetes and stable coronary artery disease: a substudy of a randomized controlled trial (RCT). *J Thromb Haemost.* 2015;13:524. doi: 10.2147/DMSO.S96299.

Njerve IU, Byrkjeland R, Arnesen H, Solheim S, Seljeflot I. Effects of long-term exercise training on adipose tissue expression of fractalkine and MCP-1 in patients with type 2 diabetes and stable coronary artery disease: a substudy of a randomized controlled trial. *Diabetes Metab Syndr Obes.* 2016;9:55-62. doi: 10.2147/DMSO.S96299.

Zaidi H, Byrkjeland R, Njerve IU, et al. Effects of exercise training on markers of adipose tissue remodeling in patients with coronary artery disease and type 2 diabetes mellitus: sub study of the randomized controlled EXCADI trial. *Diabetol Metab Syndr.* 2019;11:109. doi: 10.1186/s13098-019-0508-9.

Campo 2020
*Campo G, Tonet E, Chiaranda G, et al. Exercise intervention improves quality of life in older adults after myocardial infarction: randomised clinical trial. *Heart.* 2020;106:1658-64. doi: 10.1136/heartjnl-2019-316349.

Tonet E, Maietti E, Chiaranda G, et al. Physical activity intervention for elderly patients with reduced physical performance after acute coronary syndrome (HULK study): rationale and design of a randomized clinical trial. *BMC Cardiovasc Disord.* 2018;18(1):98. doi: 10.1186/s12872-018-0839-8.

Carlsson 1998
*Carlsson R. Serum cholesterol, lifestyle, working capacity and quality of life in patients with coronary artery disease. Experiences from a hospital-based secondary prevention programme. *Scand Cardiovasc J.* 1998;50 Suppl:1-20. doi: 10.1080/140174398427956-1.

Carson 1982

*Carson P, Phillips R, Lloyd M, et al. Exercise after myocardial infarction: a controlled trial. *J R Coll Physicians Lond.* 1982;16(3):147-51.

Chaves 2019

*Chaves GS, Ghisi GL, Britto RR, Grace SL. Maintenance of gains, morbidity, and mortality at 1 year following cardiac rehabilitation in a middle-income country: a wait-list control crossover trial. *J Am Heart Assoc.* 2019;8(4):e011228. doi: 10.1161/JAHA.118.011228.

Chaves GS, Ghisi GL, Grace SL, Oh P, Ribeiro AL, Britto RR. Corrigendum to “Effects of comprehensive cardiac rehabilitation on functional capacity and cardiovascular risk factors in Brazilians assisted by public health care: protocol for a randomized controlled trial”. *Braz J Phys Ther.* 2018;22(3):254. doi: 10.1016/j.bjpt.2018.04.007

Chaves GS, Ghisi GL, Grace SL, Oh P, Ribeiro AL, Britto RR. Effects of comprehensive cardiac rehabilitationon functional capacity and cardiovascular risk factors in Brazilians assisted by public health care: protocol for a randomized controlled trial. *Braz J Phys Ther.* 2016;20(6):592-600. doi: 10.1590/bjpt-rbf.2014.0192

Chaves GS, Ghisi GL, Grace SL, Oh P, Ribeiro AL, Britto RR. Effects of comprehensive cardiac rehabilitation on functional capacity in a middle-income country: a randomised controlled trial. *Heart.* 2019;105:406-13. doi: 10.1136/heartjnl-2018-313632

Ghisi GL, Chaves GS, Ribeiro AL, Oh P, Britto RR, Grace SL. Comprehensive cardiac rehabilitation effectiveness in a middle-income setting. A randomized controlled trial. *J Cardiopulm Rehabil.* 2020;40(6):399-406. doi: 10.1097/HCR.0000000000000512.

DeBusk 1994

*DeBusk RF, Miller NH, Superko HR, et al. A case management system for coronary risk factor modification following acute myocardial infarction. *Ann Intern Med.* 1994;120(9):721-9. doi: 10.7326/0003-4819-120-9-199405010-00001.

Taylor CB, Miller NH, Smith PM, DeBusk RF. The effect of a home-based, case-managed, multifactorial risk-reduction program on reducing psychological distress in patients with cardiovascular disease. *J Cardiopulm Rehabil* .1997;17(3):157-62. doi: 10.1097/00008483-199705000-00002

Dorje 2019

*Dorje T, Zhao G, Tso K, et al. Smartphone and social media-based cardiac rehabilitation and secondary prevention in China (SMART-CR/SP): a parallel-group, single-blind, randomised controlled trial. *Lancet.* 2019;1(7):e363-74. doi: 10.1016/S2589-7500(19)30151-7.

Dorje T, Zhao G, Tso K, et al. Correction to Lancet Digital Health 2019; published online 10 Oct 2019. *Lancet Digit Health.* 2019. doi: 10.1016/S2589-7500(19)30162-1

Dorje T, Zhao G, Scheer A, et al. SMARTphone and social media-based Cardiac Rehabilitation and Secondary Prevention (SMART-CR/SP) for patients with coronary heart disease in China: a randomised controlled trial protocol. *BMJ Open.* 2018;8:e021908. doi: 10.1136/bmjopen-2018-021908.

SMARTphone-based Home Cardiac Rehabilitation and Secondary Prevention in Chinese Coronary Heart Disease Patients (SMART-CR/SP): a randomized controlled trial. chictr.org.cn identifier: ChiCTR-INR-16009598. www.chictr.org.cn/hvshowproject.aspx?id=12793 Updated October, 2016. Accessed December 16, 2021.

Dugmore 1999

*Dugmore LD, Tipson RJ, Phillips MH, et al. Changes in cardiorespiratory fitness, psychological wellbeing, quality of life, and vocational status following a 12 month cardiac exercise rehabilitation programme. *Heart.* 1999;81(4):359-66. doi: 10.1136/hrt.81.4.359.

Engblom 1996
*Engblom E, Korpilahti K, Hamalainen H, Puukka P, Ronnemaa T. Effects of five years of cardiac rehabilitation after coronary artery bypass grafting on coronary risk factors. *Am J Cardiol.* 1996;78:1428-31. doi: 10.1016/s0002-9149(96)00629-7

Engblom E, Hamalainen H, Lind J, et al. Quality of life during rehabilitation after coronary bypass surgery. *Qual Life Res.* 1992;1:167-75. doi: 10.1007/BF00635616.

Engblom E, Hietanen EK, Hamalainen H, Kallio V, Inberg M, Knuts L-R. Exercise habits and physical performance during comprehensive rehabilitation after coronary artery bypass surgery. *Eur Heart J.* 1992;13:1053-9. doi: 10.1093/oxfordjournals.eurheartj.a060313.

Engblom E, Korpilahti K, Hamalainen H, Ronnemaa T, Puukka P. Quality of life and return to work 5 years after coronary artery bypass surgery. *J Cardiopulm Rehabil.* 1997;17:29-36. doi: 10.1097/00008483-199701000-00004.

Engblom E, Rönnemaa T, Hämäläinen H, Kallio V, Vänttinen, Knuts LR. Coronary heart disease risk factors before and after bypass surgery: results of a controlled trial on multifactorial rehabilitation. *Eur Heart J.* 1992;13(2):232-7. doi: 10.1093/oxfordjournals.eurheartj.a060152.

Erdman 1986
*Erdman RA, Duivenvoorden HJ, Verhage F, Kazemier M, Hugenholtz PG. Predictability of beneficial effects in cardiac rehabilitation: a randomized clinical trial of psychosocial variables. *J Cardiopulm Rehabil.* 1986;6(6):206-13. doi:10.1097/00008483-198606000-00001

Fletcher 1994
*Fletcher BJ, Dunbar SB, Felner JM, et al. Exercise testing and training in physically disabled men with clinical evidence of coronary artery disease. *Am J Cardiol.* 1994;73(2):170-4. doi: 10.1016/0002-9149(94)90209-7.

Fridlund 1991
*Fridlund B, Högstedt B, Lidell E, Larsson PA. Recovery after myocardial infarction: effects of a caring rehabilitation programme. *Scand J Caring Sci.* 1991;5(1):23-32. doi: 10.1111/j.1471-6712.1991.tb00078.x.

Fridlund B, Lidell E, Larsson PA. A caring perspective on rehabilitation after myocardial infarction: a theoretical framework and a suggestion for a rehabilitation programme. *Scand J Caring Sci.* 1989;3(3):129-35. doi: 10.1111/j.1471-6712.1989.tb00385.x.

Fridlund B, Pihilgren C, Wannestig LB. A supportive - educative caring rehabilitation programme: improvements of physical health after myocardial infarction. *J Clin Nurs.* 1992;1:141-6. doi: 10.1093/ptj/pzaa159.

Lidell E, Fridlund B. Long-term effects of a comprehensive rehabilitation programme after myocardial infarction. *Scand J Caring Sci.* 1996;10:67-74. doi: 10.1111/j.1471-6712.1996.tb00314.x.

Giallauria 2008
*Giallauria F, Cirillo P, Lucci R, et al. Left ventricular remodelling in patients with moderate systolic dysfunction after myocardial infarction: favourable effects of exercise training and predictive role of N-terminal pro-brain natriuretic peptide. *Eur J Cardiovasc Prev Rehabil.* 2008;15(1):113-8. doi: 10.1097/HJR.0b013e3282f00990.

Hambrecht 2004
*Hambrecht R, Walther C, Mobius-Winkler S, et al. Percutaneous coronary angioplasty compared with exercise training in patients with stable coronary artery disease: a randomized trial. *Circulation.* 2004;109:1371-8. doi: 10.1161/01.CIR.0000121360.31954.1F

Walther C, Mobius-Winkler S, Linke A, et al. Regular exercise training compared with percutaneous intervention leads to a reduction of inflammatory markers and cardiovascular events in patients with coronary artery disease. *Eur J Cardiovasc Prev Rehabil.* 2008;15:107-12. doi: 10.1097/HJR.0b013e3282f29aa6.

Haskell 1994
*Haskell WL, Alderman EL, Fair JM, et al. Effects of intensive multiple risk factor reduction on coronary atherosclerosis and clinical cardiac events in men and women with coronary artery disease: the Stanford Coronary Risk Intervention Project (SCRIP). *Circulation.* 1994;89(3):975-90. doi: 10.1161/01.cir.89.3.975.

Hassan 2016
*Hassan AM, Nahas NG. Efficacy of cardiac rehabilitation after percutaneous coronary intervention. *International Journal of PharmTech Research.* 2016;9(4):134-41.

Hautala 2017
*Hautala AJ, Kiviniemi AM, Makikallio T, et al. Economic evaluation of exercise-based cardiac rehabilitation in patients with a recent acute coronary syndrome. *Scand J Med Sci Sports.* 2017;27(11):1395-403. doi: 10.1111/sms.12738.

Effectiveness of exercise cardiac rehabilitation (EFEX-CARE). clinicaltrials.gov identifier: NCT01916525. https://clinicaltrials.gov/ct2/show/NCT01916525 Updated August 2013. Accessed December 16, 2021.

He 2020
*He C, Zhu C, Zhu Y, Zou Z, Wang S, Zhai C, Hu H. Effect of exercise-based cardiac rehabilitation on clinical outcomes in patients with myocardial infarction in the absence of obstructive coronary artery disease (MINOCA). *Int J Cardiol.* 2020;315:9-14. doi: 10.1016/j.ijcard.2020.05.019.

Heller 1993
*Heller RF, Knapp JC, Valenti LA, Dobson AJ. Secondary prevention after acute myocardial infarction. *Am J Cardiol.* 1993;72(11):759-62. doi: 10.1016/0002-9149(93)91058-p.

Higgins 2001
*Higgins HC, Hayes RL, McKenna KT. Rehabilitation outcomes following percutaneous coronary interventions (PCI). *Patient Educ Couns.* 2001;43:219-30. doi: 10.1016/s0738-3991(00)00164-6.

Hofman-Bang 1999
*Hofman-Bang C, Lisspers J, Nordlander R, et al. Two-year results of a controlled study of residential rehabilitation for patients treated with percutaneous transluminal coronary angioplasty. A randomized study of a multifactorial programme. *Eur Heart J.* 1999;20(20):1465-74. doi: 10.1053/euhj.1999.1544.

Lisspers J, Sundin Ö, Öhman A, Hofman-Bang C, Rydén L, Nygren Å. Long-term effects of lifestyle behavior change in coronary artery disease: effects on recurrent coronary events after percutaneous coronary intervention. *Health Psychol.* 2005;24(1):41-8. doi: 10.1037/0278-6133.24.1.41.

Lisspers J, Sundin Ö, Hofman-Bang C, et al. Behavioral effects of a comprehensive multifactorial program for lifestyle change after percutaneous transluminal coronary angioplasty: a prospective randomized, controlled study. *J Psychosom Res.* 1999;46(2):143-54. doi: 10.1016/s0022-3999(98)00074-9.

Holmback 1994
*Holmbäck AM, Säwe U, Fagher B. Training after myocardial infarction: Lack of long-term effects on physical capacity and psychological variables. *Arch Phys Med Rehabil.* 1994;75(5):551-4.

Houle 2012
*Houle J, Doyon O, Vadeboncoeur N, Turbide G, Diaz A, Poirier P. Effectiveness of a pedometer-based program using a socio-cognitive intervention on physical activity and quality of life in a setting of cardiac rehabilitation. *Can J Cardiol.* 2012;28:27-32. doi: 10.1016/j.cjca.2011.09.020.

Kallio 1979
*Kallio V, Hämäläinen H, Hakkila J, Luurila OJ. Reduction in sudden deaths by a multifactorial intervention programme after acute myocardial infarction. *Lancet.* 1979;2(8152):1019-4. doi: 10.1016/s0140-6736(79)92502-9.

Kovoor 2006
*Kovoor P, Lee AK, Carrozzi F, et al. Return to full normal activities including work at two weeks after acute myocardial infarction. *Am J Cardiol.* 2006;97(7):952-8. doi: 10.1016/j.amjcard.2005.10.040.

Hall JP, Wiseman VP, King MT, et al. Economic evaluation of a randomised trial of early return to normal activities versus cardiac rehabilitation after acute myocardial infarction. *Heart Lung Circ.* 2002;11:10-8. doi: 10.1046/j.1444-2892.2002.00105.x.

La Rovere 2002
*La Rovere MT, Bersano C, Gnemmi M, Specchia G, Schwartz PJ. Exercise-induced increase in baroreflex sensitivity predicts improved prognosis after myocardial infarction. *Circulation.* 2002;106(8):945-9. doi: 10.1161/01.cir.0000027565.12764.e1.

Lear 2015
*Lear SA, Singer J, Banner-Lukaris D, et al. Improving access to cardiac rehabilitation using the internet: a randomized trial. *Stud Health Technol Inform.* 2015;209:58-66.

Lear SA, Singer J, Banner-Lukaris D, et al. Randomized trial of a virtual cardiac rehabilitation program delivered at a distance via the internet. *Circ Cardiovasc Qual Outcomes.* 2014;7:952-9. doi: 10.1161/CIRCOUTCOMES.114.001230.

Leizorovicz 1991
*Leizorovicz A, Saint-Pierre A, Vasselon C, Boissel JP. Comparison of a rehabilitation programme, a counselling programme and usual care after an acute myocardial infarction: results of a long-term randomized trial. P.RE.COR. Group. *Eur Heart J.* 1991;12(5):612-6. doi: 10.1093/oxfordjournals.eurheartj.a059948.

Lewin 1992
*Lewin B, Robertson IH, Cay EL, Irving JB, Campbell M. Effects of self-help post-myocardial infarction rehabilitation on psychological adjustment and use of health services. *Lancet.* 1992;339(8800):1036-40. doi: 10.1016/0140-6736(92)90547-g.

Ma 2020

*Ma LY, Deng L, Yu H. The effects of a comprehensive rehabilitation and intensive education program on anxiety, depression, quality of life, and major adverse cardiac and cerebrovascular events in unprotected left main coronary artery disease patients who underwent coronary artery bypass grafting. *Ir J Med Sci.* 2020;189:477-88. doi: 10.1007/s11845-019-02129-x.

Maddison 2014
*Maddison R, Pfaeffli L, Whittaker R, et al. A mobile phone intervention increases physical activity in people with cardiovascular disease: results from the HEART randomized controlled trial. *Eur J Prev Cardiol.* 2014;22(6):701-9. doi: 10.1177/2047487314535076.

Manchanda 2000
*Manchanda SC, Narang R, Reddy KS, et al. Retardation of coronary atherosclerosis with yoga lifestyle intervention. *J Assoc Physicians India.* 2000;48(7):687-94.

Marchionni 2003
*Marchionni N, Fattirolli F, Fumagalli S, et al. Improved exercise tolerance and quality of life with cardiac rehabilitation of older patients after myocardial infarction: results of a randomized, controlled trial. *Circulation.* 2003;107(17):2201-6. doi: 10.1161/01.CIR.0000066322.21016.4A.

Maroto 2005
*Maroto MJ, Artigao Ramirez R, Morales Duran MD, de Pablo Zarzosa C, Abraira V. Cardiac rehabilitation in patients with myocardial infarction: a 10-year follow-up study. *Rev Esp Cardiol.* 2005;58:1181-7.

Miller 1984
*Miller NH, Haskell WL, Berra K, DeBusk RF. Home versus group exercise training for increasing functional capacity after myocardial infarction. *Circulation.* 1984;70(4):645-9. doi: 10.1161/01.cir.70.4.645.

DeBusk RF, Haskell WL, Miller NH, et al. Medically directed at-home rehabilitation soon after clinically uncomplicated acute myocardial infarction: a new model for patient care. *Am J Cardiol.* 1985;55(4):251-7. doi: 10.1016/0002-9149(85)90355-8.

Taylor CB, Houston-Miller N, Ahn DK, Haskell WL, DeBusk RF. The effects of exercise training programs on psychosocial improvement in uncomplicated postmyocardial infarction patients. *J Psychosom Res.* 1986;30(5):581-7. doi: 10.1016/0022-3999(86)90031-0.

Taylor CB, Houston-Miller N, Haskell WL, DeBusk RF. Smoking cessation after acute myocardial infarction: the effects of exercise training. *Addict Behav.* 1988;13(4):331-5. doi: 10.1016/0306-4603(88)90039-1.

Munk 2009
*Munk PS, Staal EM, Butt N, Isaksen K, Larsen AI. High-intensity interval training may reduce in-stent restenosis following percutaneous coronary intervention with stent implantation. *Am Heart J.* 2009;158:734-41. doi: 10.1016/j.ahj.2009.08.021.

Munk PS, Breland UM, Aukrust P, Ueland T, Kvaloy JT, Larsen AI. High intensity interval training reduces systemic inflammation in post-PCI patients. *Eur J Cardiovasc Prev Rehabil.* 2011;18:850-7. doi: 10.1177/1741826710397600.

Mutwalli 2012
*Mutwalli HA, Fallows SJ, Arnous AA, Zamzami MS. Randomized controlled evaluation shows the effectiveness of a home-based cardiac rehabilitation program. *Saudi Med J.* 2012;33:152-9.

Oerkild 2012
*Oerkild B, Frederiksen M, Hansen JF, Prescott E. Home-based cardiac rehabilitation is an attractive alternative to no cardiac rehabilitation for elderly patients with coronary heart disease: results from a randomised clinical trial. *BMJ Open.* 2012;2:e001820. doi: 10.1136/bmjopen-2012-001820.

Oldridge 1991
*Oldridge N, Guyatt G, Jones N, et al. Effects on quality of life with comprehensive rehabilitation after acute myocardial infarction. *Am J Cardiol.* 1991;67(13):1084-9. doi: 10.1016/0002-9149(91)90870-q.

Oldridge N, Furlong W, Feeny D, et al. Economic evaluation of cardiac rehabilitation soon after acute myocardial infarction. *Am J Cardiol.* 1993;72:154-61. doi: 10.1016/0002-9149(93)90152-3.

Oldridge N, Streiner D, Hoffmann R, Guyatt G. Profile of mood states and cardiac rehabilitation after acute myocardial infarction. *Med Sci Sports Exerc.* 1995;27(6):900-5.

Ornish 1990
*Ornish D, Brown SE, Scherwitz LW, et al. Can lifestyle changes reverse coronary heart disease? The Lifestyle Heart Trial. *Lancet.* 1990;336(8708):129-33. doi: 10.1016/0140-6736(90)91656-u.

Ornish D, Scherwitz LW, Billings JH, et al. Intensive lifestyle changes for reversal of coronary heart disease. *JAMA.* 1998;280(23):2001-7. doi: 10.1001/jama.280.23.2001.

Pischke CR, Scherwitz L, Weidner G, Ornish D. Long-term effects of lifestyle changes on well-being and cardiac variables among coronary heart disease patients. *Health Psychol.* 2008;27(5):584-92. doi: 10.1037/0278-6133.27.5.584.

Pal 2013
*Pal A, Srivastava N, Narain VS, Agrawal GG, Rani M. Effect of yogic intervention on the autonomic nervous system in the patients with coronary artery disease: a randomized controlled trial. *East Mediterr Health J.* 2013;19(5):453-8.

Pomeshkina 2017
*Pomeshkina SA, Loktionova EB, Bezzubova VA, Arkhipova NV, Borovik IV, Barbarash OL. The comparative analysis of the influence of the supervised exercise training and home-based exercise training on the psychological status of the following coronary artery bypass grafting. *Problems of Balneology, Physiotherapy, and Exercise Therapy.* 2017;94(6):10-7. doi: 10.17116/kurort201794610-17.

Pomeshkina S, Loktionova E, Arkhipova N, Barbarash O. Home-based walking training and adherence to medical therapy in patients undergoing coronary artery bypass grafting. *Eur Heart J.* 2015;36:634. doi: 10.1371/journal.pone.0041199.

Pomeshkina 2019
*Pomeshkina SA, Barbarash OL, Pomeshkin EV. Exercise training and erectile dysfunction in patients after coronary artery bypass grafting. *Therapeutic Archive.* 2019;91(9):16-20. doi: 10.26442/00403660.2019.09.000149.

Prabhakaran 2020
*Prabhakaran D, Chandrasekaran AM, Singh K, et al. Yoga-based cardiac rehabilitation after acute myocardial infarction. *J Am Coll Cardiol.* 2020;75(13):1551-61. doi: 10.1016/j.jacc.2020.01.050.

Chandrasekaran AM, Kinra S, Ajay VS, et al. Effectiveness and cost-effectiveness of a yoga-based cardiac rehabilitation (Yoga-CaRe) program following acute myocardialinfarction:

study rationale and design of a multi-center randomized controlled trial. *Int J Cardiol.* 2019;280:14-8. doi: 10.1016/j.ijcard.2019.01.012.

Chattopadhyay K, Chandrasekaran AM, Praveen PA, et al. Development of a yoga-based cardiac rehabilitation (Yoga-CaRe) programme for secondary prevention of myocardial infarction. *Evid Based Complement Alternat Med.* 2019;2019:1-7. doi: 10.1155/2019/7470184.

A study on effectiveness of yoga based cardiac rehabilitation programme in India and United Kingdom. chictr.org.cn identifier: CTRI/2012/02/002408. http://www.ctri.nic.in/Clinicaltrials/pmaindet2.php?trialid=3992 Updated January 2021. Accessed December 16, 2021.

Reid 2012
*Reid DR, Morrin LI, Beaton LJ, et al. Randomized trial of an internet-based computer-tailored expert system for physical activity in patients with heart disease. *Eur J Prev Cardiol.* 2012;19(6):1357–64. doi: 10.1177/1741826711422988

Roman 1983
*Roman O, Gutierrez M, Luksic I, et al. Cardiac rehabilitation after acute myocardial infarction. 9-year controlled follow-up study. *Cardiology.* 1983;70:223-31. doi: 10.1159/000173598.

Sandstrom 2005
*Sandström L, Ståhle A. Rehabilitation of elderly with coronary heart disease - Improvement in quality of life at a low cost. *Adv Physiother.* 2005;7:60-6. doi:10.1080/14038190510010287

Santaularia 2017
*Santaularia N, Caminal J, Arnau A, et al. The efficacy of a supervised exercise training programme on readmission rates in patients with myocardial ischemia: results from a randomised controlled trial. *Eur J Cardiovasc Nurs.* 2017;16(3):201-12. doi: 10.1177/1474515116648801.

Santaularia N, Caminal J, Arnau A, et al. Randomized clinical trial to evaluate the effect of a supervised exercise training program on readmissions in patients with myocardial ischemia: a study protocol. *BMC Cardiovasc Disord.* 2013;13(1):32. doi: 10.1186/1471-2261-13-32.

Schuler 1992
*Schuler G, Hambrecht R, Schlierf G, et al. Regular physical exercise and low-fat diet. Effects on progression of coronary artery disease. *Circulation.* 1992;86(1):1-11. doi: 10.1161/01.cir.86.1.1.

Nikolaus T, Schlierf G, Vogel G, Schuler G, Wagner I. Treatment of coronary heart disease with diet and exercise: problems of compliance. *Ann Nutr Metab.* 1991;35:1-7. doi: 10.1159/000177615.

Niebauer J, Hambrecht R, Velich T, et al. Predictive value of lipid profile for salutary coronary angiographic changes in patients on a low-fat diet and physical exercise program. *Am J Cardiol.* 1996;78(2):163-7. doi: 10.1016/s0002-9149(96)90390-2.

Niebauer J, Hambrecht R, Velich T, et al. Attenuated progression of coronary artery disease after 6 years of multifactorial risk intervention: role of physical exercise. *Circulation.* 1997;96(8):2534-41. doi: 10.1161/01.cir.96.8.2534.

Niebauer J, Hambrecht R, Marburger C, et al. Impact of intensive physical exercise and low-fat diet on collateral vessel formation in stable angina pectoris and angiographically

confirmed coronary artery disease. *Am J Cardiol.* 1995;76(11):771-5. doi: 10.1016/s0002-9149(99)80224-0.

Hambrecht R, Niebauer J, Marburger C, et al. Various intensities of leisure time physical activity in patients with coronary artery disease: effects on cardiorespiratory fitness and progression of coronary atherosclerotic lesions. *J Am Coll Cardiol.* 1993;22(2):468-77. doi: 10.1016/0735-1097(93)90051-2.

Seki 2003
*Seki E, Watanabe Y, Sunayama S, et al. Effects of phase III cardiac rehabilitation programs on health-related quality of life in elderly patients with coronary artery disease: Juntendo Cardiac Rehabilitation Program (J-CARP). *Circ J.* 2003;67(1):73-7. doi: 10.1253/circj.67.73.

Seki 2008
*Seki E, Watanabe Y, Shimada K, et al. Effects of a phase III cardiac rehabilitation program on physical status and lipid profiles in elderly patients with coronary artery disease: Juntendo Cardiac Rehabilitation Program (J-CARP). *Circ J.* 2008;72(8):1230-4. doi: 10.1253/circj.72.1230.

Shaw 1981
*Shaw LW. Effects of a prescribed supervised exercise program on mortality and cardiovascular morbidity in patients after a myocardial infarction. The National Exercise and Heart Disease Project. *Am J Cardiol.* 1981;48(1):39-46. doi: 10.1016/0002-9149(81)90570-1.

Stern MJ, Cleary P. The National Exercise and Heart Disease Project: long-term psychosocial outcome. *Arch Intern Med.* 1982;142(6):1093-7.

132. Naughton J. The National Exercise and Heart Disease Project. The pre-randomization exercise program. Report number 2. *Cardiology.* 1978;63(6):352-67. doi: 10.1159/000169916.

Dorn J, Naughton J, Imamura D, Trevisan M. Results of a multicenter randomized clinical trial of exercise and long-term survival in myocardial infarction patients: the National Exercise and Heart Disease Project (NEHDP). *Circulation.* 1999;100:1764-9. doi: 10.1161/01.cir.100.17.1764.

Sivarajan 1982
*Sivarajan ES, Bruce RA, Lindskog BD, Almes MJ, Belanger L, Green B. Treadmill test responses to an early exercise program after myocardial infarction: a randomized study. *Circulation.* 1982;65(7):1420-8. doi: 10.1161/01.cir.65.7.1420.

Ott CR, Sivarajan ES, Newton KM, et al. A controlled randomized study of early cardiac rehabilitation: the sickness impact profile as an assessment tool. *Heart Lung.* 1983;12(2):162-70.

Sivarajan ES, Bruce RA, Almes MJ, et al. In-hospital exercise after myocardial infarction does not improve treadmill performance. *N Engl J Med.* 1981;305(7):357-62. doi: 10.1056/NEJM198108133050701.

Sivarajan ES, Newton KM, Almes MJ, Kempf TM, Mansfield LW, Bruce RA. Limited effects of outpatient teaching and counselling after myocardial infarction: A controlled study. *Heart Lung.* 1983;12(1):65-73.

Snoek 2020
*Snoek JA, Prescott EI, Van der Velde AE, et al. Effectiveness of home-based mobile guided cardiac rehabilitation as alternative strategy for nonparticipation in clinic-based cardiac

rehabilitation among elderly patients in Europe. A randomized clinical trial. *JAMA Cardiol.* 2020;6(4):463–8. doi: 10.1001/jamacardio.2020.5218.

Specchia 1996
*Specchia G, De Servi S, Scirè A, et al. Interaction between exercise training and ejection fraction in predicting prognosis after a first myocardial infarction. *Circulation.* 94(5):978-82. doi: 10.1161/01.cir.94.5.978.

Stahle 1999
*Ståhle A, Mattsson E, Rydén L, Unden AL, Nordlander R. Improved physical fitness and quality of life following training of elderly patients after acute coronary events. A 1 year follow-up randomized controlled study. *Eur Heart J.* 1999;20(20):1475-84. doi: 10.1053/euhj.1999.1581.

Hage C, Mattsson E, Ståhle A. Long term effects of exercise training on physical activity level and quality of life in elderly coronary patients - a three- to six-year follow-up. *Physiother Res Int.* 2003;8(1):13-22. doi: 10.1002/pri.268.

Ståhle A, Lindquist I, Mattsson E. Important factors for physical activity among elderly patients one year after an acute myocardial infarction. *Scand J Rehabil Med.* 2000;32(3):111-6. doi: 10.1080/003655000750045451.

Ståhle A, Nordlander R, Rydén L, Mattsson E. Effects of organized aerobic group training in elderly patients discharged after an acute coronary syndrome. A randomized controlled study. *Scand J Rehabil Med.* 1999;31(2):101-7. doi: 10.1080/003655099444614.

Tollbäck A, Ståhle A. Effects of aerobic group training on exercise capacity, muscular endurance and recovery in elderly patients with recent coronary events: a randomized, controlled study. *Adv Physiother.* 2001;3:29-37. doi: 10.1080/140381901300039305.

Stern 1983
*Stern MJ, Gorman PA, Kaslow L. The group counseling v exercise therapy study. A controlled intervention with subjects following myocardial infarction. *Arch Intern Med.* 1983;143(9):1719-25.

Sun 2016
*Sun P, Li Y, Song C, et al. Long-term effects of exercise rehabilitation on risk factors in elderly patients with stable coronary artery disease. *Chinese Journal of Geriatric Heart Brain and Vessel Diseases.* 2016;5:491-5.

Toobert 2000
*Toobert DJ, Glasgow RE, Radcliffe JL. Physiologic and related behavioral outcomes from the Women's Lifestyle Heart Trial. *Ann Behav Med.* 2000;22(1):1-9. doi: 10.1007/BF02895162.

Toobert DJ, Glasgow RE, Nettekoven LA, Brown JE. Behavioral and psychosocial effects of intensive lifestyle management for women with coronary heart disease. *Patient Educ Couns.* 1998;35(3):177-88. doi: 10.1016/s0738-3991(98)00074-3.

Uddin 2020
*Uddin J, Joshi VL, Moniruzzaman M, et al. Effect of home-based cardiac rehabilitation in a lower-middle income country: results from a controlled trial. *J Cardiopulm Rehabil Prev.* 2020;40(1):29-34. doi: 10.1097/HCR.0000000000000471.

Uddin J. Effects of cardiac rehabilitation on exercise capacity and WHO-quality of life undergoing CABG patients. A quasi-randomised controlled trial. *Eur J Heart Fail.* 2017;19:464. doi: 10.1002/ejhf.833.

Vecchio 1981
*Vecchio C, Cobelli F, Opasich C, Assandri J, Poggi G, Griffo R. Early functional evaluation and physical rehabilitation in patients with wide myocardial infarction [Valutazione funzionale precoce e riabilitazione fisica nei pazienti con infarto miocardico esteso]. *G Ital Cardiol.* 1981;11:419-29.

Vermeulen 1983
*Vermeulen A, Lie KI, Durrer D. Effects of cardiac rehabilitation after myocardial infarction: changes in coronary risk factors and long-term prognosis. *Am Heart J.* 1983;105(5):798-801. doi: 10.1016/0002-8703(83)90243-0.

VHSG 2003
*Vestfold Heartcare Study Group. Influence on lifestyle measures and five-year coronary risk by a comprehensive lifestyle intervention programme in patients with coronary heart disease. *Eur J Cardiovasc Prev Rehabil.* 2003;10(6):429-37. doi: 10.1097/01.hjr.0000107024.38316.6a.

Schumacher A, Peersen K, Sommervoll L, Seljeflot I, Arnesen H, Otterstad JE. Physical performance is associated with markers of vascular inflammation in patients with coronary heart disease. *Eur J Cardiovasc Prev Rehabil.* 2006;13(3):356-62. doi: 10.1097/01.hjr.0000188244.54287.96.

Wang 2012
*Wang W, Chair SY, Thompson DR, Twinn SF. Effects of home-based rehabilitation on health-related quality of life and psychological status in Chinese patients recovering from acute myocardial infarction. *Heart Lung.* 2012;41:15-25. doi: 10.1016/j.hrtlng.2011.05.005.

West 2012
*West RR, Jones DA, Henderson AH. Rehabilitation after myocardial infarction trial (RAMIT): multi-centre randomised controlled trial of comprehensive cardiac rehabilitation in patients following acute myocardial infarction. *Heart.* 2012;98:637-44. doi: 10.1136/heartjnl-2011-300302.

WHO 1983
*World Health Organization. *Rehabilitation and comprehensive secondary prevention after acute myocardial infarction.* EURO Reports and Studies 841983; 1983.

Wilhelmsen 1975
*Wilhelmsen L, Sanne H, Elmfeldt D, Grimby G, Tibblin G, Wedel H. A controlled trial of physical training after myocardial infarction. Effects on risk factors, nonfatal reinfarction, and death. *Prev Med.* 1975;4(4):491-508. doi: 10.1016/0091-7435(75)90035-3.

Sanne H. Exercise tolerance and physical training of non-selected patients after myocardial infarction. *Acta Med Scand.* 1973;Supplementum 551:1-124.

Xu 2017
*Xu Y, Feng Y, Su P, Li Y, Li C, Qiao J. Impact of exercise rehabilitation on cardiac function in coronary artery disease patients after percutaneous coronary intervention. *Chinese Circulation Journal.* 2017;32:326-30.

Yu 2003
*Yu CM, Li LS, Ho HH, Lau CP. Long-term changes in exercise capacity, quality of life, body anthropometry, and lipid profiles after a cardiac rehabilitation program in obese patients with coronary heart disease. *Am J Cardiol.* 2003;91(3):321-5. doi: 10.1016/s0002-9149(02)03159-4.

Yu 2004
*Yu CM, Lau CP, Chau J, et al. A short course of cardiac rehabilitation program is highly cost effective in improving long-term quality of life in patients with recent myocardial infarction or percutaneous coronary intervention. *Arch Phys Med Rehabil.* 2004;85(12):1915-22. doi: 10.1016/j.apmr.2004.05.010.

Yu C, Li L, Lam M, Siu D, Miu R, Lau C. Effect of a cardiac rehabilitation program on left ventricular diastolic function and its relationship to exercise capacity in patients with coronary heart disease: experience from a randomized, controlled study. *Am Heart J.* 2004;147(5):e24. doi: 10.1016/j.ahj.2003.12.004.

Zhang 2018
*Zhang Y, Cao H, Jiang P, Tang H. Cardiac rehabilitation in acute myocardial infarction patients after percutaneous coronary intervention: a community-based study. *Medicine.* 2018;97(8):1-5. doi: 10.1097/MD.0000000000009785.

Zwisler 2008
*Zwisler AD, Soja AM, Rasmussen S, et al. Hospital-based comprehensive cardiac rehabilitation versus usual care among patients with congestive heart failure, ischemic heart disease, or high risk of ischemic heart disease: 12-month results of a randomized clinical trial. *Am Heart J.* 2008;155(6):1106-13. doi: 10.1016/j.ahj.2007.12.033.

Kruse M, Hochstrasser S, Zwisler AD, Kjellberg J. Comprehensive cardiac rehabilitation: a cost assessment based on a randomized clinical trial. *Int J Technol Assess Health Care.* 2006;22(4):478-83. doi: 10.1017/S0266462306051403.

Supplementary file 3:

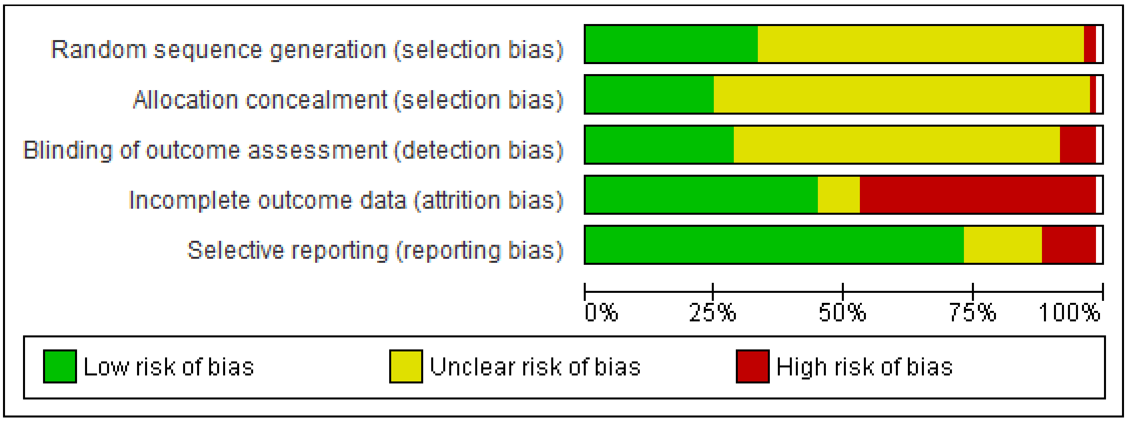

Supplementary Figure 1: Risk of bias assessment

Supplementary file 4:

Supplementary table 1: Summary of health-related quality of life (HRQoL) scores at follow-up

Measure of HRQoL	Mean (SD) outcome values at follow-up		P value	Difference between groups
	**Exercise**	**Control**		
**Aronov 2019**				
**Quality of life questionnaire developed by authors (Aronov 2002) % change of mean score at 6 months**				
	**% change**	**% change**		
	30.4	"no change"	NR	
**Bell 1998**				
**Nottingham health profile at 10.5 months follow-up:**				
Energy	17.6 (27.1)	18.3 (29.8)	0.87**	Exercise = Control
Pain	2.8 (8.8)	4.82 (11.9)	<0.05	Exercise > Control
Emotional reactions	6.4 (17.0)	12.2 (19.9)	<0.001	Exercise > Control
Sleep	7.5 (18.4)	20.5 (27.8)	<0.001	Exercise > Control
Social isolation	2.3 (10.6)	4.0 (13.3)	0.37*	Exercise = Control
Physical mobility	8.4 (11.1)	8.9 (14.5)	0.82**	Exercise = Control
**Belardinelli 2001**				
**SF-36 at 6 months follow-up:**				
Physical functioning	78 (19)	55 (20)	0.001	Exercise > Control
Physical performance	75 (13)	65 (14)	0.01	Exercise > Control
Bodily pain	4 (9)	22 (10)	0.001	Exercise > Control
General health	68 (14)	50 (19)	0.001	Exercise > Control
Vitality	NR	NR		
Social functioning	66 (10)	69 (12)	0.14*	Exercise = Control
Emotional performance	NR	NR		
Mental health	65 (12)	48 (15)	0.01	Exercise > Control
**SF-36 at 12 months follow-up:**				
Physical functioning	82 (18)	54 (20)	0.001	Exercise > Control
Physical performance	76 (9)	58 (14)	0.01	Exercise > Control
Bodily pain	4 (9)	32 (12)	0.001	Exercise > Control
General health	70 (14)	50 (18)	0.001	Exercise > Control
Vitality	NR	NR		
Social functioning	68 (11)	68 (12)	1.00*	Exercise = Control
Emotional performance	NR	NR		
Mental health	70 (14)	45 (15)	0.001	Exercise > Control
**Bettencourt 2005**				
**SF-36 at 1 year follow-up:**				
Physical functioning	70	62	NS*	Exercise = Control
Physical performance	66	57	NS*	Exercise = Control
Bodily pain	73	65	NS*	Exercise = Control
General health	57	46	< 0.02	Exercise > Control
Vitality	62	47	< 0.02	Exercise > Control
Social functioning	73	66	NS*	Exercise = Control
Emotional performance	65	58	NS*	Exercise = Control
Mental health	87	75	NS*	Exercise = Control
Mental component	71	57	0.02	Exercise > Control
Physical component	63	57	NS*	Exercise = Control
**Briffa 2005**				
**SF-36 at 6 months follow-up:**				
	**Δ (95% CI)**	**Δ (95% CI)**		
Physical functioning	15.9 (−8 to 23)	7.1 (1 to 13)	NS*	Exercise = Control

Physical performance	75 (0 to 100)	75 (0 to 100)	NS*	Exercise = Control
Bodily pain	26.6 (18 to 35)	19.2 (11 to 27)	NS*	Exercise = Control
General health	0.1 (−6 to 6)	− 0.6 (-5 to 4)	NS*	Exercise = Control
Vitality	7.1 (1 to 13)	3.7 (-2 to 9)	NS*	Exercise = Control
Social functioning	19.6 (10 to 29)	14.1 (7 to 21)	NS*	Exercise = Control
Emotional performance	33.3 (0 to 100)	33.3 (33 to 100)	NS*	Exercise = Control
Mental health	0.5 (−4 to 5)	1.4 (-3 to 5)	NS*	Exercise = Control
**SF-36 at 1 year follow-up:**				
	**Δ (95% CI)**	**Δ (95% CI)**		
Physical functioning	17.6 (10 to 25)	6.8 (−1 to 14)	0.04	Exercise > Control
Physical performance	100 (0 to 100)	75 (12 to 30)	NS*	Exercise = Control
Bodily pain	30.2 (23 to 37)	20.9 (−2 to 7)	NS*	Exercise = Control
General health	2.7 (−3 to 5)	2.2 (−2 to 7)	NS*	Exercise = Control
Vitality	11.9 (6 to 18)	6.9 (1 to 12)	NS*	Exercise = Control
Social functioning	23.6 (14 to 33)	16.4 (9 to 23)	NS*	Exercise = Control
Emotional performance	33.3 (33 to 100)	33.3 (33 to 100)	NS*	Exercise = Control
Mental health	3.6 (−1 to 9)	3.9 (0 to 8)	NS*	Exercise = Control
**Bubnova 2019**				
**Quality of life questionnaire developed by authors (Aronov 2002) mean (sd) score after 12 months:**				
Low rehabilitation potential subgroup	-4.9 (4.5)	-7.8 (3.1)	<0.05	Exercise > Control
Average rehabilitation potential subgroup	-5 (3.2)	-7.4 (4.3)	<0.05	Exercise > Control
High rehabilitation potential subgroup	-4.3 (3.9)	-5.6 (4.3)	<0.05	Exercise > Control
**Bubnova 2020**				
**Quality of life questionnaire developed by authors (Aronov 2002) mean (%) score change at 12 months:**				
	**Δ (%)**	**Δ (%)**		
BMI <30 kg/m^2 group	42 (6%)	10 (2%)	<0.01	Exercise > Control
BMI ≥30 kg/m^2 group	27 (5%)	8 (2%)	<0.001	Exercise > Control
**Campo 2020**				
**EuroQol at 6 months follow up:**				
	**Median (IQR)**	**Median (IQR)**		
**VAS (visual analogue scale)**	80 (70-90)	70 (50-80)	<0.001	Exercise > Control
**5 domains**	**N (%)**	**N (%)**		
**Pain/discomfort:**			0.03	Exercise > Control
No	103 (89)	89 (77)		
Moderate	10 (9)	24 (21)		
Extreme	3 (3)	3 (3)		
**Anxiety/depression:**			0.001	Exercise > Control
No	92 (79)	67 (58)		
Moderate	21 (18)	36 (31)		
Extreme	3 (3)	12 (10)		
**Mobility:**			<0.001	Exercise > Control
No problems	104 (90)	80 (70)		
Some problems	12 (10)	34 (30)		
Confined to bed	0 (0)	1 (1)		
**Self-care:**			0.6	Exercise = Control
No problems	114 (98)	87 (76)		
Some problems	2 (2)	25 (22)		
Unable	0 (0)	1 (1)		
**Usual activities:**			0.04	Exercise > Control
No problems	101 (87)	87 (76)		

Some problems	14 (12)	25 (22)		
Unable	1 (1)	3 (3)		
**EuroQol at 12 months follow up:**				
	**Median (IQR)**	**Median (IQR)**		
**VAS (visual analogue scale)**	75 (70-87)	65 (50-80)	<0.001	Exercise > Control
**5 domains**	N (%)	N (%)		
**Pain/discomfort:**			0.04	Exercise > Control
No	86 (77)	72 (65)		
Moderate	24 (21)	29 (26)		
Extreme	2 (2)	9 (8)		
**Anxiety/depression:**			0.03	Exercise > Control
No	83 (74)	58 (53)		
Moderate	23 (21)	37 (34)		
Extreme	6 (5)	15 (14)		
**Mobility:**			0.008	Exercise > Control
No problems	95 (85)	74 (67)		
Some problems	16 (14)	22 (20)		
Confined to bed	1 (1)	3 (3)		
**Self-care:**			0.8	Exercise = Control
No problems	101 (91)	100 (91)		
Some problems	6 (5)	5 (5)		
Unable	3 (3)	5 (5)		
**Usual activities:**			0.004	Exercise > Control
No problems	99 (88)	80 (73)		
Some problems	11 (10)	24 (22)		
Unable	2 (1)	6 (5)		
**Dorje 2019**				
**SF-12 at 6 months follow up:**				
Physical health score	46.8 (6.9)	45.2 (6.5)	0.22**	Exercise = Control
Mental health score	51.5 (9.3)	50 (8.6)	0.28**	Exercise = Control
**Engblom 1992**				
**Nottingham health profile at 5 years follow-up:**				
Energy	18	25	0.08	Exercise = Control
Pain	12	18	0.07	Exercise = Control
Emotional reactions	14	21	0.27	Exercise = Control
Sleep	24	29	0.42	Exercise = Control
Social isolation	7	9	0.42	Exercise = Control
Physical mobility	6	14	0.005	Exercise > Control
**Hassan 2016**				
**SF-36 8 domains at 12 months follow up**				
Physical functioning	83.5 (6.5)	76.7 (10.6)	0.01	Exercise > Control
Role limitations physical	62.5 (23.4)	50.8 (20.2)	0.04	Exercise > Control
Role limitations emotional	61.1 (21.6)	49.9 (19.1)	0.04	Exercise > Control
Energy/fatigue	66 (11.1)	57.7 (11.7)	0.01	Exercise > Control
Emotional wellbeing	69.5 (2.6)	61.5 (7.5)	0.000	Exercise > Control
Social functioning	67.5 (19)	56.3 (16.3)	0.02	Exercise > Control
Pain	79.6 (18.4)	67.9 (15.9)	0.01	Exercise > Control
General health	43 (7.9)	38.5 (8.8)	0.04	Exercise > Control
**Hautala 2017**				
**15D Quality of life measure at 6 months follow up:**				
	0.915 (0.07)	0.876 (0.084)	0.0004*	Exercise > Control
**15D Quality of life measure at 12 months follow up:**				
	0.922 (0.072)	0.886 (0.088)	<0.0015*	Exercise > Control
**He 2020**				
**SF-36 at 12 months:**				
Physical functioning	85 (22)	74 (19)	<0.01	Exercise > Control

Role-physical	80 (21)	77 (22)	0.362	Exercise = Control
Bodily pain	71 (32)	68 (30)	0.348	Exercise = Control
General health	79 (23)	72 (19)	<0.01	Exercise > Control
Vitality	81 (17)	73 (25)	<0.01	Exercise > Control
Social functioning	75 (22)	74 (19)	0.902	Exercise = Control
Role-emotional	65 (34)	65 (33)	0.976	Exercise = Control
Mental health	72 (23)	71 (23)	0.825	Exercise = Control
Physical health score	79 (29)	73 (29)	<0.01	Exercise > Control
Mental health score	73 (28)	71 (27)	0.102	Exercise = Control
**Heller 1993**				
**QLMI at 6 months follow-up:**				
Emotional	5.4 (1.1)	5.2 (1.2)	0.04	Exercise > Control
Physical	5.4 (1.2)	5.2 (1.3)	0.17*	Exercise = Control
Social	5.9 (1.1)	5.8 (1.1)	0.35*	Exercise = Control
**Hofman-Bang 1999**				
**AP-QLQ at 12 months follow-up:**				
Physical activity	4.9	4.3	<0.05	Exercise > Control
Somatic symptoms	NR	NR	NS	Exercise = Control
Emotional distress	NR	NR	NS	Exercise = Control
Life satisfaction	NR	NR	NS	Exercise = Control
**Houle 2012**				
**Quality of Life Index-cardiac version III at 6 months follow-up:**				
Health and functional score	26 (5.1)	24.5 (5.3)	0.048	Exercise > Control
Psychological/spiritual score	25.6 (5.8)	25.5 (3.8)	0.383	Exercise = Control
Social and economic score	25.7 (3.8)	25.4 (4.7)	0.392	Exercise = Control
Family score	28.1 (2.5)	26.7 (4.3)	0.048	Exercise > Control
Overall	26.2 (4.3)	25.8 (4.1)	0.057	Exercise = Control
**Quality of Life Index-cardiac version III at 12 months follow-up:**				
Health and functional score	27.8 (2.0)	25.3 (4.6)	0.036	Exercise > Control
Psychological/spiritual score	27.4 (2.5)	26.2 (4.0)	0.336	Exercise = Control
Social and economic score	27.2 (3.0)	25.9 (5.2)	0.638	Exercise = Control
Family score	28 (2.6)	26.8 (5.0)	0.092	Exercise = Control
Overall	27.7 (2.1)	25.7 (4.2)	0.048	Exercise > Control
**Ma 2020**				
**SF-12 change at 12 months follow up:**				
	**Δ (SD)**	**Δ (SD)**		
Physical component	13.3 (6)	9.9 (5.9)	<0.001	Exercise > Control
Mental component	12.4 (5.4)	9 (6.2)	<0.001	Exercise > Control
**Maddison 2014**				
**EQ-5D at 24 weeks follow-up:**				
	0.86	0.83	0.23	Exercise = Control
**SF-36 at 24 weeks follow-up:**				
Physical functioning	52.9	51.9	0.20	Exercise = Control
Role physical	52.6	50.8	0.08	Exercise = Control
Bodily pain	52.4	51.9	0.71	Exercise = Control
General health	55.3	53.2	0.03	Exercise > Control
Vitality	55.7	55.9	0.79	Exercise = Control
Social Functioning	53.3	52.4	0.42	Exercise = Control
Role emotional	51.4	51.6	0.81	Exercise = Control
Mental health	54.6	54.0	0.61	Exercise = Control
**Mutwalli 2012**				
**SF-36 Health status score at 6 months follow-up:**				
	90.14 (4.83)	60.55 (16.21)	0.000	Exercise > Control

**Oerkild 2012**				
**SF-36 at 12 months follow-up:**				
	**Δ (95% CI)**	**Δ (95% CI)**		
SF 12 PCS	-1.1 (-5.3 to 3.1)	-1.4 (-5.2 to 2.3)	NS*	Exercise = Control
SF 12 MCS	-1.4 (-6.1 to 3.3)	-0.3 (-4.6 to 4.0)	NS*	Exercise = Control
**Oldridge 1991**				
**QLMI at 4 months follow-up:**				
Limitations	54	54	NS	Exercise = Control
Emotions	103	101	NS	Exercise = Control
**QLMI at 8 months follow-up:**				
Limitations	54	54	NS	Exercise = Control
Emotions	103	103	NS	Exercise = Control
**QLMI at 12 months follow-up:**				
Limitations	54	55	NS	Exercise = Control
Emotions	105	102	NS	Exercise = Control
**Reid 2012**				
**MacNew at 6 months follow-up:**				
Global score	5.8 (0.6)	5.6 (0.8)	0.112	Exercise = Control
Emotional subscale	5.6 (0.6)	5.4 (0.7)	0.038	Exercise > Control
Social subscale	6.3 (0.8)	6.0 (1.0)	0.162	Exercise = Control
Physical subscale	6.0 (0.8)	5.8 (1.0)	0.031	Exercise > Control
**Sandstrom 2005**				
**Time Trade Off (TTO) at 12 months follow-up:**				
	0.86 (0.23)	0.85 (0.21)	NS*	Exercise = Control
**EuroQol Part one at 12 months follow-up:**				
	0.87 (0.15)	0.86 (0.16)	NS*	Exercise = Control
**EuroQol Part two at 12 months follow-up:**				
	7.6 (1.46)	7.43 (1.46)	NS*	Exercise = Control
**Santaularia 2017**				
**EuroQol-5D at 12 months follow up:**				
	N (%)	N(%)		
**Mobility**			0.019	Exercise > Control
No problems	33 (84.6)	33 (75)		
Problems	6 (15.4)	11 (25)		
**Self-care**			0.172	Exercise = Control
No problems	38 (97.4)	43 (97.7)		
Problems	1 (2.6)	1 (2.3)		
**Usual activities**			0.803	Exercise = Control
No problems	32 (82)	31 (70.5)		
Problems	7 (18)	13 (29.5)		
**Pain/discomfort**			0.528	Exercise = Control
No problems	28 (71.8)	26 (59.1)		
Problems	11 (28.2)	18 (40.9)		
**Anxiety/depression**			0.429	Exercise = Control
No problems	22 (56.4)	26 (59.1)		
Problems	17 (43.6)	18 (40.9)		
**Snoek 2020**				
**SF-36 summary scores at 6 months:**				
Physical	50.2 (7.2)	48.3 (7.5)	0.086*	Exercise = Control
Mental	54.0 (8.4)	52.7 (9.1)	0.322*	Exercise = Control
**SF-36 summary scores at 12 months:**				
Physical	50.6 (7.2)	49 (8.2)	0.167*	Exercise = Control
Mental	53.2 (8.8)	52.5 (9.2)	0.604*	Exercise = Control
**Stahle 1999**				
**Karolinska Questionnaire at 12 months follow-up:**				
Chest pain	0.6 (1.2)	0.4 (1.3)	NS	Exercise = Control
Shortness of breath	0.4 (1.1)	0.2 (1.0)	NS	Exercise = Control
Dizziness	-0.1 (1.1)	0.2 (0.9)	NS	Exercise = Control
Palpitation	-0.1 (1.0)	0.1 (0.9)	NS	Exercise = Control

Cognitive ability	-0.1 (0.6)	0.0 (0.7)	NS	Exercise = Control
Alertness	0.0 (0.9)	0.1 (0.8)	NS	Exercise = Control
Quality of sleep	0.0 (0.5)	0.1 (0.5)	NS	Exercise = Control
Physical ability	0.2 (0.7)	0.1 (0.4)	NS	Exercise = Control
Daily activity	0.3 (0.5)	0.1 (0.5)	NS	Exercise = Control
Depression	0.1 (0.3)	0.1 (0.2)	NS	Exercise = Control
Self-perceived health	0.5 (1.3)	0.3 (1.0)	NS	Exercise = Control
"Ladder of Life" present	1.2 (1.2)	0.9 (1.8)	NS	Exercise = Control
"Ladder of Life" future	0.8 (2.7)	0.4 (2.3)	NS	Exercise = Control
Fitness	0.6 (1.4)	0.4 (1.0)	NS	Exercise = Control
Physical ability	0.7 (1.0)	0.4 (1.1)	NS	Exercise = Control
**Toobert 2000**				
**SF-36 at 24 months follow-up:**				
Physical functioning	NR	NR	NS	Exercise = Control
Physical performance	NR	NR	NS	Exercise = Control
Bodily pain	NR	NR	NS	Exercise = Control
General health	NR	NR	<0.05	Exercise > Control
Vitality	NR	NR	NS	Exercise = Control
Social functioning	NR	NR	<0.05	Exercise > Control
Emotional performance	NR	NR	NS	Exercise = Control
Mental health	NR	NR	NS	Exercise = Control
**Uddin 2020**				
**WHOQoL-BREF at 12 months follow up**				
Overall perception of HRQoL	4.03 (0.49)	3.2 (0.82)	<0.01	Exercise > Control
Overall perception of health	4.06 (0.4)	3.17 (0.38)	<0.01	Exercise > Control
Physical domain	26.9 (2.88)	21.17 (3.35)	<0.01	Exercise > Control
Psychological domain	23.42 (2.84)	17.87 (3.19)	<0.01	Exercise > Control
Social relationship domain	11.83 (1.5)	10.75 (0.89)	<0.01	Exercise > Control
Environmental domain	28.8 (4.24)	21.77 (5.31)	0.03	Exercise > Control
**Wang 2012**				
**SF-36 at 6 months follow-up:**				
Physical functioning	80.8 (13.7)	73.2 (13.0)	<0.001	Exercise > Control
Physical performance	68.2 (17.3)	56.2 (46.8)	0.015	Exercise > Control
Bodily pain	68.2 (17.3)	63.5 (14.6)	0.012	Exercise > Control
General health	57.4 (20.3)	49.0 (16.2)	0.017	Exercise > Control
Vitality	66.3 (17.3)	56.4 (21.7)	0.002	Exercise > Control
Social functioning	71.3 (21.4)	65.8 (18.0)	0.031	Exercise > Control
Emotional performance	80.8 (37.9)	75.9 (39.7)	0.12	Exercise = Control
Mental health	73.5 (17.1)	65.4 (20.7)	0.011	Exercise > Control
**MIDAS at 6 months follow up:**				
Physical Activity	37.7 (11.2)	42.6 (12.3)	<0.001	Exercise > Control
Insecurity	28.7 (9.7)	33.4 (13.8)	<0.001	Exercise > Control
Emotional reaction	30.4 (12.8)	34.8 (14.4)	0.008	Exercise > Control
Dependency	27.6 (9.4)	31.8 (16.6)	0.001	Exercise > Control
Diet	36.8 (15.4)	43.6 (20.7)	0.40	Exercise = Control
Concerns over meds	29.4 (12.6)	37.7 (18.0)	<0.001	Exercise > Control
Side Effects	28.2 (11.1)	30.8 (14.3)	0.30	Exercise > Control
**West 2012**				
**SF-36 at 12 months follow-up:**				
Physical function	65 (29)	64 (30)	NS*	Exercise = Control
Role physical	69 (31)	67 (33)	NS*	Exercise = Control
Role emotional	85 (23)	85 (25)	NS*	Exercise = Control
Social function	81 (28)	79 (29)	NS*	Exercise = Control

Mental health	76 (13)	76 (13)	NS*	Exercise = Control
Energy /vitality	65 (24)	65 (24)	NS*	Exercise = Control
Pain	69 (28)	68 (29)	NS*	Exercise = Control
Health Perception	58 (25)	57 (25)	NS*	Exercise = Control
**Yu 2003**				
**SF-36 at 8 months follow-up:**				
Physical functioning	88 (12)	82 (17)	0.03*	Exercise > Control
Physical performance	75 (33)	66 (35)	0.18*	Exercise = Control
Bodily pain	80 (25)	80 (25)	1.00*	Exercise = Control
General health	64 (26)	60 (28)	0.45*	Exercise = Control
Vitality	79 (18)	65 (17)	0.0001	Exercise > Control
Social functioning	89 (27)	82 (28)	0.15	Exercise = Control
Emotional performance	93 (18)	83 (35)	0.05	Exercise = Control
Mental health	84 (16)	80 (15)	0.2	Exercise = Control
**SF-36 at 24 months follow-up:**				
Physical functioning	88 (13)	87 (9)	0.67*	Exercise = Control
Physical performance	80 (32)	79 (30)	0.87*	Exercise = Control
Bodily pain	81 (21)	85 (20)	0.33*	Exercise = Control
General health	64 (20)	61 (18)	0.43*	Exercise = Control
Vitality	73 (21)	73 (17)	1.00*	Exercise = Control
Social functioning	79 (30)	90 (18)	0.04*	Exercise > Control
Emotional performance	89 (25)	93 (25)	0.42*	Exercise = Control
Mental health	85 (14)	85 (12)	1.00*	Exercise = Control
**Zwisler 2008**				
**SF-36 at 12 months follow-up:**				
Physical Component Score	45.2 (9.8)	46.4 (9.8)	0.39*	Exercise = Control
Mental Component Score	50.6 (10.8)	48.4 (11.5)	0.16*	Exercise = Control

Footnotes

AP-QLQ: Angina Pectoris-Quality of Life questionnaire; BMI: body mass index; EQ-5D: five-dimension EuroQol scale; EuroQoL: European Quality of Life Scale; IQR: interquartile range; MIDAS: Myocardial Infarction Dimensional Assessment Scale; NR: not reported; NS: not significant; QLMI: Quality of Life After Myocardial Infarction questionnaire; SD: standard deviation; SF-36: Short Form 36-item questionnaire; WHOQoL-BREF: World Health Organization Quality of Life abbreviated instrument

* Calculated by authors of this report based on independent two group t test.

** Adjusted for baseline difference between groups.

Exercise = Control: no statistically significant difference (P > 0.05) between exercise and Control groups at follow up

Exercise > Control: statistically significant difference (P < 0.05) between exercise and Control groups at follow up

NS*: The authors of this review have inferred a P value of > 0.05 based either on the 95% CI, or from narrative in the paper, rather than from directly observing the P-value.

Supplementary file 5:

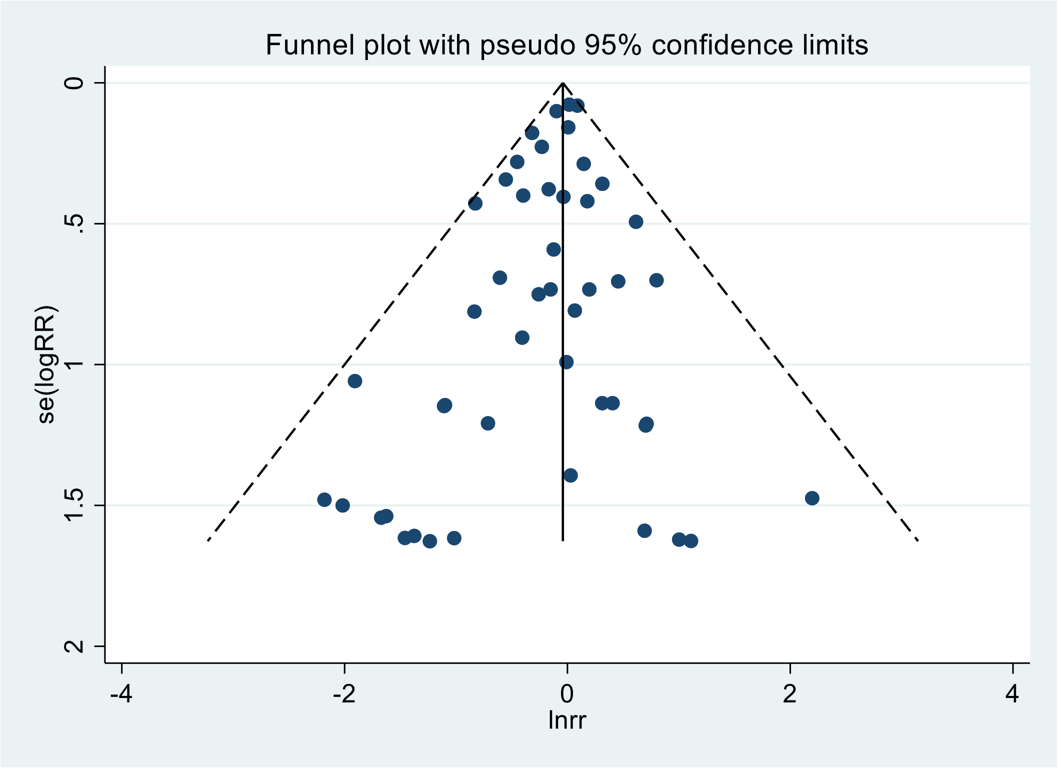

Supplementary Figure 2: Funnel plot – exercise-based CR vs control for all-cause mortality

Supplementary file 6:

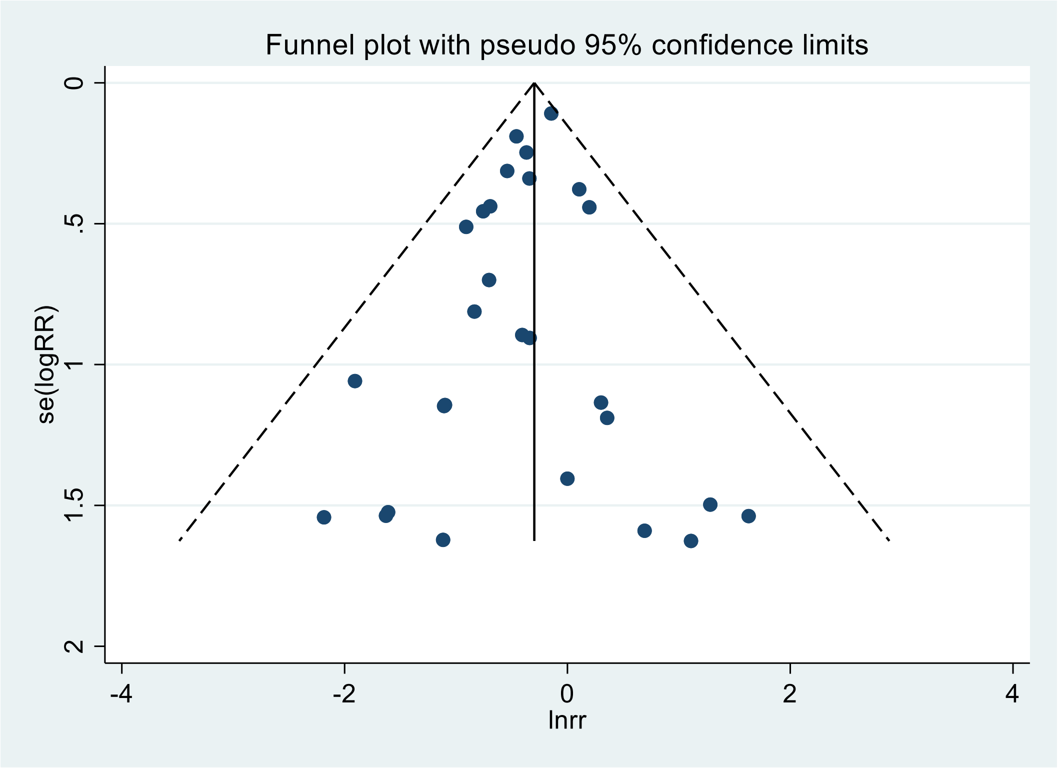

Supplementary Figure 3: Funnel plot – exercise-based CR vs control for cardiovascular mortality

Supplementary file 7:

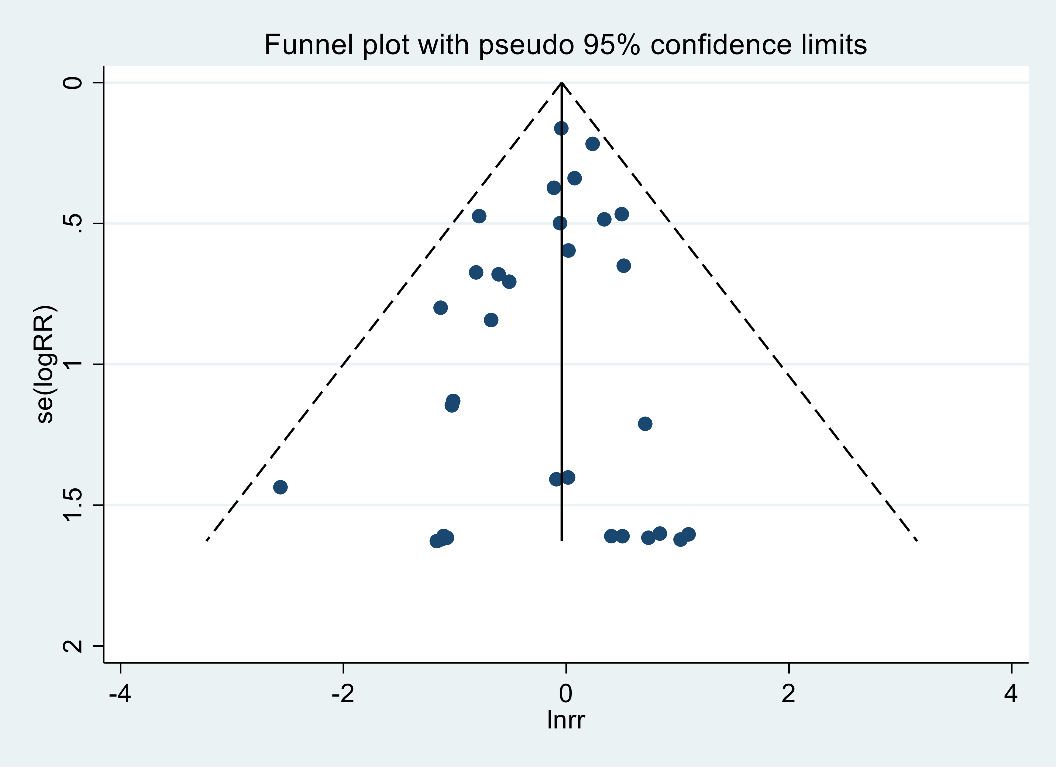

Supplementary Figure 4: Funnel plot – exercise-based CR vs control for CABG

Supplementary file 8:

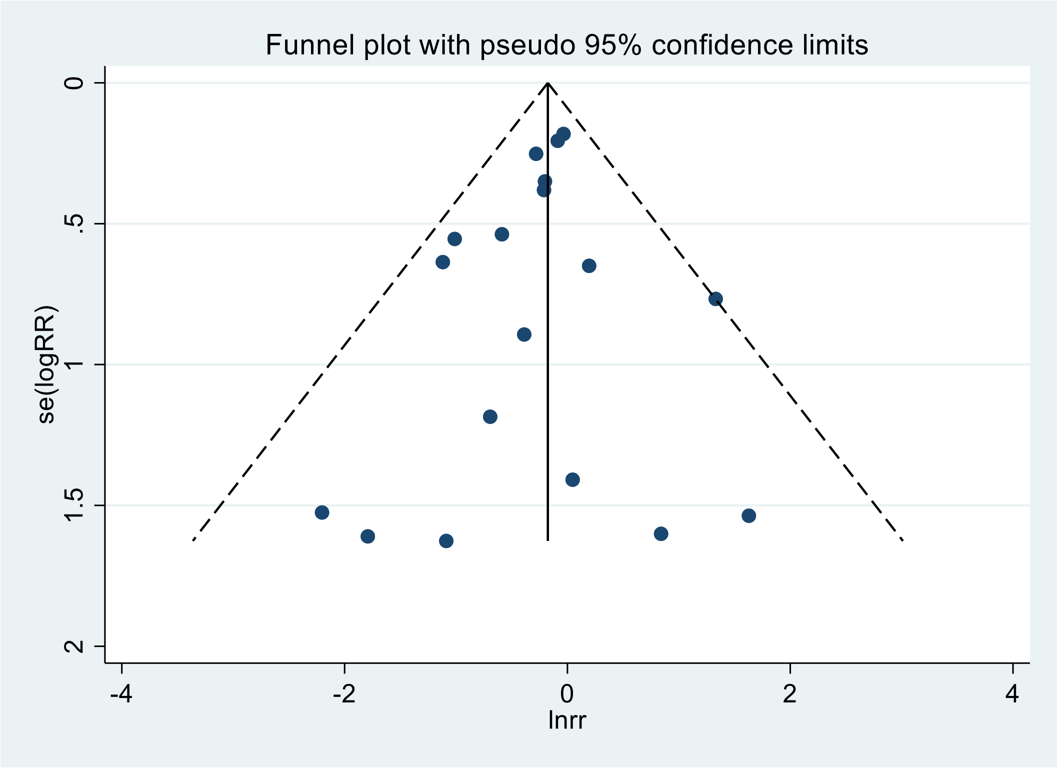

Supplementary Figure 5: Funnel plot – exercise-based CR vs control for PCI

Supplementary file 9:

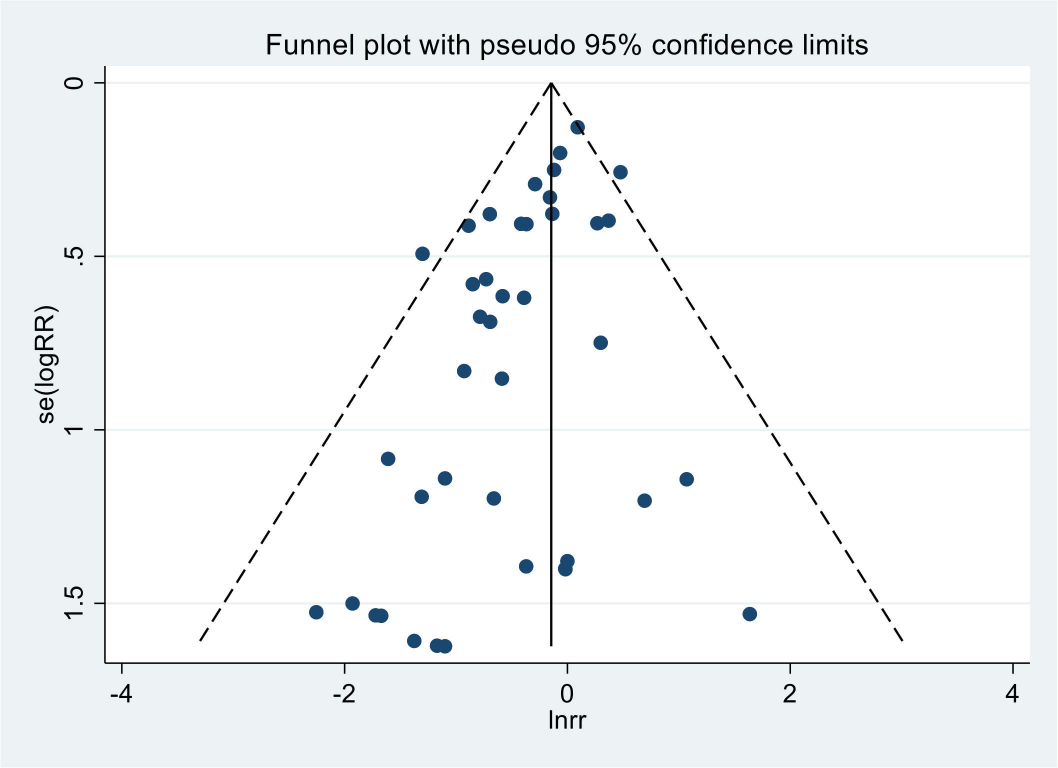

Supplementary Figure 6: Funnel plot – exercise-based CR vs control for MI

Supplementary file 10:

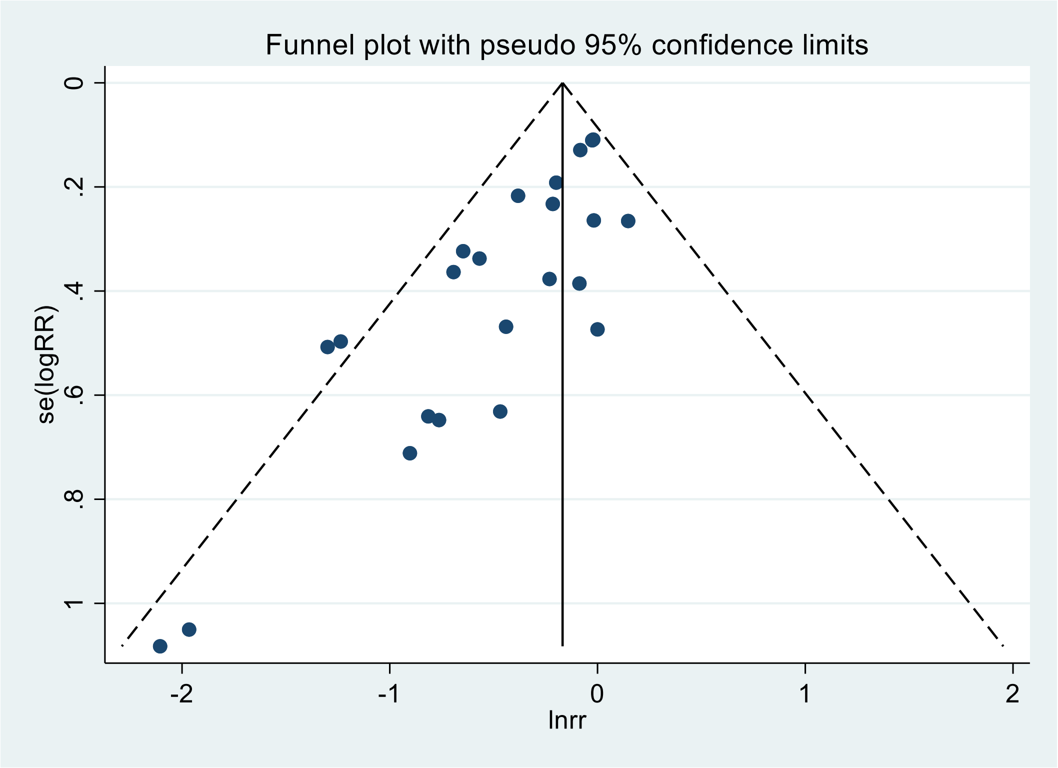

Supplementary Figure 7: Funnel plot – exercise-based CR vs control for all-cause hospitalisation

Supplementary file 11:

Supplementary Table 2: Results for univariate meta-regression for primary clinical outcomes

RR (95% CI)

Explanatory variable	All-cause mortality	CV mortality	MI	CABG	PCI	All-cause hospitalisation
Case mix (% MI patients)	1.00 (1.00 to 1.00)	1.00 (0.99 to 1.01)	1.00 (0.99 to 1.00)	1.01 (1.00 to 1.02)	1.00 (1.00 to 1.01)	1.00 (1.00 to 1.01)
Dose of exercise†	1.00 (1.00 to 1.00)	1.00 (1.00 to 1.00)	1.00 (1.00 to 1.00)	1.00 (1.00 to 1.00)	1.00 (1.00 to 1.00)	1.00 (1.00 to 1.00)
Duration of follow up	1.00 (1.00 to 1.00)	0.99 (0.99 to 1.00)	1.00 (0.99 to 1.01)	1.00 (0.99 to 1.01)	1.00 (0.99 to 1.01)	1.01 (1.00 to 1.01)
Type of CR‡	1.04 (0.83 to 1.30)	0.83 (0.62 to 1.10)	0.85 (0.58 to 1.25)	1.04 (0.67 to 1.61)	0.78 (0.38 to 1.59)	0.93 (0.65 to 1.33)
Year of publication§	0.84 (0.70 to 0.99)	1.37 (0.89 to 2.13)	1.36 (0.94 to 1.97)	0.88 (0.56 to 1.41)	0.95 (0.46 to 1.95)	1.12 (0.80 to 1.57)
CR setting#	1.01 (0.82 to 1.24)	1.05 (0.88 to 1.24)	0.80 (0.67 to 0.95)	1.07 (0.87 to 1.33)	0.91 (0.72 to 1.15)	0.94 (0.83 to 1.06)
Risk of bias	1.02 (0.94 to 1.09)	0.90 (0.73 to 0.11)	1.39 (0.85 to 2.26)	0.94 (0.64 to 1.38)	1.09 (0.72 to 1.66)	1.00 (0.71 to 1.40)
Continent	1.01 (0.86 to 1.19)	1.02 (0.75 to 1.39)	0.71 (0.49 to 1.05)	1.19 (0.83 to 1.71)	0.81 (0.53 to 1.23)	0.86 (0.69 to 1.08)
LMIC	1.00 (0.70 to 1.45)	0.69 (0.22 to 2.19)	0.65 (0.33 to 1.61)	0.51 (0.08 to 3.18)	0.29 (0.05 to 1.63)	1.06 (0.72 to 1.55)
Sample size¶	1.19 (0.73 to 1.93)	1.28 (0.69 to 2.37)	1.69 (1.05 to 2.72)	1.31 (0.82 to 2.09)	1.19 (0.70 to 2.01)	1.45 (1.08 to 1.96)

CV, cardiovascular; MI, myocardial infarction; CABG, coronary artery bypass graft; PCI, percutaneous coronary intervention; CR, cardiac rehabilitation; LMIC, low-middle income country.
Values are relative risk (95% confidence interval). *P<0.005; † dose (units) = number of weeks of exercise training x average number of sessions/week x average duration of session in minutes; ‡ exercise only vs comprehensive CR; § pre 1995 vs post 1995; # home vs centre; ¶ ≤150 vs >150 participants

Supplementary file 12:

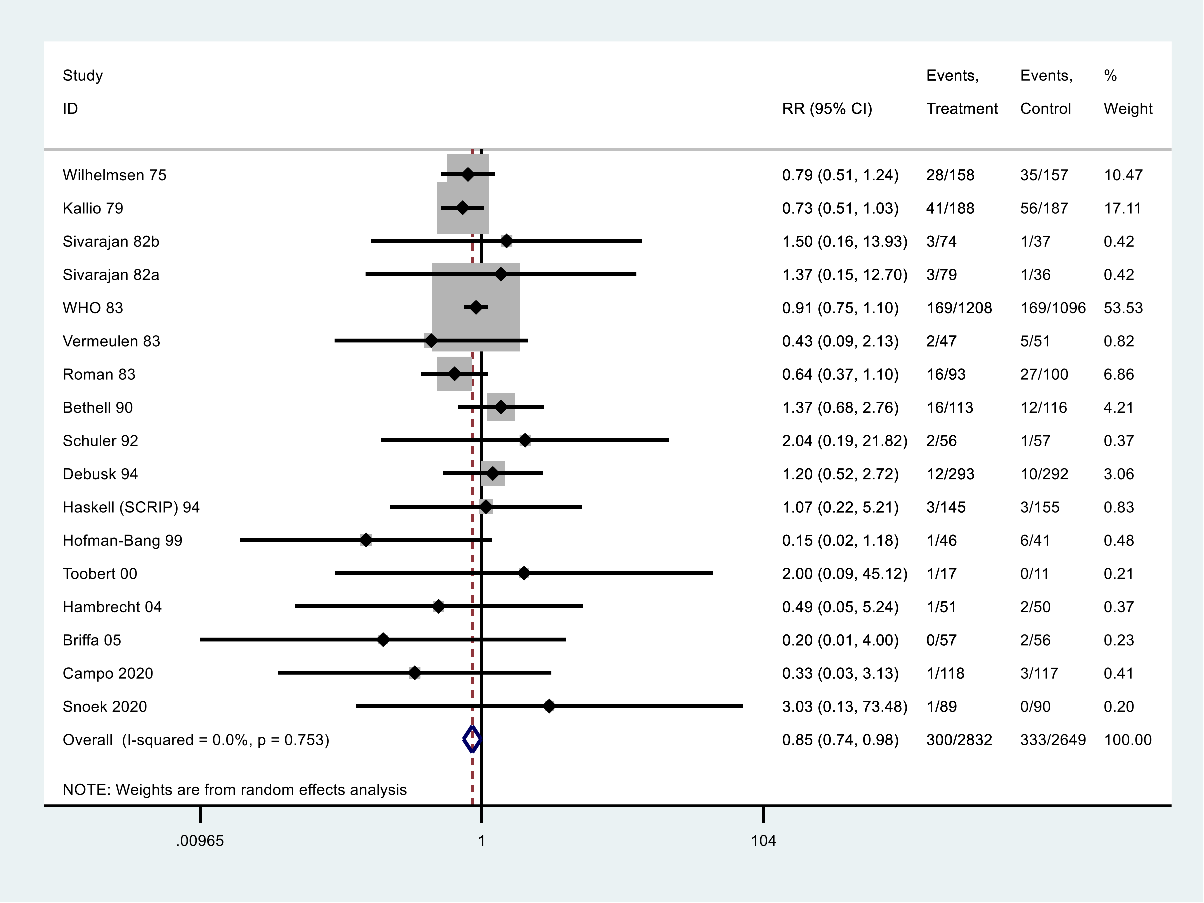

Supplementary Figure 8: Forest plot – exercise-based CR vs control for all-cause mortality in trials that report both all-cause mortality and cardiovascular mortality data

Supplementary file 13:

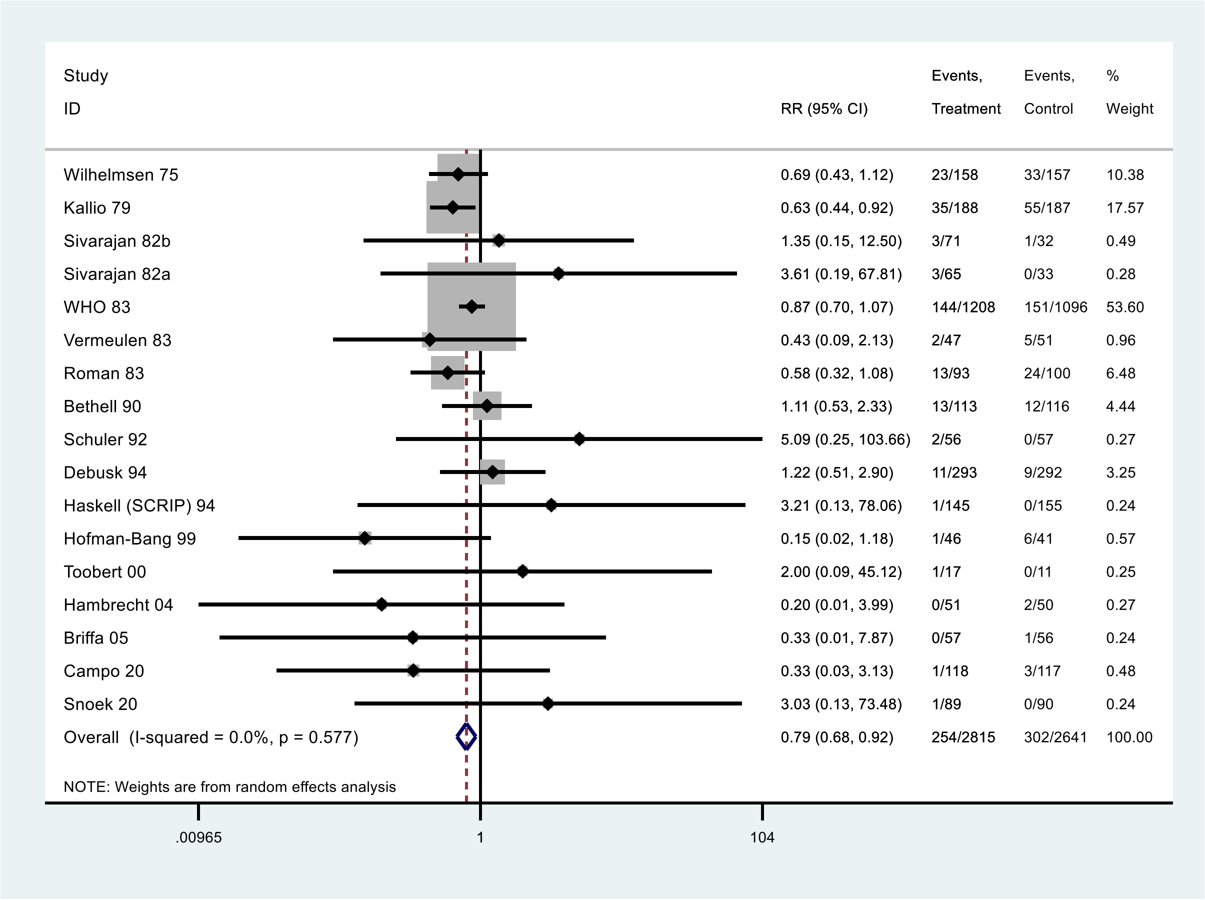

Supplementary Figure 9: Forest plot – exercise-based CR vs control for CV mortality in trials that report both all-cause mortality and cardiovascular mortality data
